# Gene Prioritization by Compressive Data Fusion and Chaining

**DOI:** 10.1371/journal.pcbi.1004552

**Published:** 2015-10-14

**Authors:** Marinka Žitnik, Edward A. Nam, Christopher Dinh, Adam Kuspa, Gad Shaulsky, Blaž Zupan

**Affiliations:** 1 Faculty of Computer and Information Science, University of Ljubljana, Ljubljana, Slovenia; 2 Department of Molecular and Human Genetics, Baylor College of Medicine, Houston, Texas, United States of America; 3 Department of Biochemistry and Molecular Biology, Baylor College of Medicine, Houston, Texas, United States of America; ETH Zurich, SWITZERLAND

## Abstract

Data integration procedures combine heterogeneous data sets into predictive models, but they are limited to data explicitly related to the target object type, such as genes. Collage is a new data fusion approach to gene prioritization. It considers data sets of various association levels with the prediction task, utilizes collective matrix factorization to compress the data, and chaining to relate different object types contained in a data compendium. Collage prioritizes genes based on their similarity to several seed genes. We tested Collage by prioritizing bacterial response genes in *Dictyostelium* as a novel model system for prokaryote-eukaryote interactions. Using 4 seed genes and 14 data sets, only one of which was directly related to the bacterial response, Collage proposed 8 candidate genes that were readily validated as necessary for the response of *Dictyostelium* to Gram-negative bacteria. These findings establish Collage as a method for inferring biological knowledge from the integration of heterogeneous and coarsely related data sets.

## Author Summary

In everyday life, we make decisions by considering all the available information, and often find that inclusion of even seemingly circumstantial evidence provides an advantage. Our new computational method Collage prioritizes genes from a large collection of heterogeneous data. In a case study on social amoeba *Dictyostelium*, we started from four bacterial response genes and 14 different data sets ranging from gene expression to pathway and literature information. Collage proposed eight candidate genes that were tested in the wet laboratory. Mutations in all eight candidates reduced the ability of the amoebae to grow on Gram-negative bacteria. Furthermore, five out of the eight candidate genes were required for growth on Gram-negative bacteria but had no discernible effect on growth on Gram-positive bacteria. This is a remarkably accurate result since only about a hundred of the 12,000 *Dictyostelium* genes are estimated to be responsible for bacterial response.

## Introduction

In the natural sciences, incorporating all the data, especially circumstantial information, can be conceptually and computationally challenging. The difficulty stems from the heterogeneity and abundance of data sets. Consider a typical data analysis task in molecular biology: besides experimental data, such as levels of gene expression, there are plenty of other data sets at our disposal, such as protein-protein binding sites, genetic and metabolic pathways, functional annotations, genetic interactions, phenotype ontologies, diseases, drugs and their side effects. Intuitively, collective mining of all available information sources should improve accuracy of predictive modeling. However, the challenges are to integrate seemingly unrelated concepts from heterogeneous data sets [1] and fuse various data sets into a single predictive model.

Here we present a method called Collage that can consider a large number of potentially indirectly related data sets and use them for gene prioritization. Computational prediction of gene function is a formidable challenge. Given a small set of seed genes that are known to be responsible for a particular function, gene prioritization [2] aims to identify the most promising candidates for further studies. Present data integration approaches for gene prioritization can be divided into four groups: methods that consecutively filter one data set at a time [3]; methods that stitch together gene profiles from different data sources and then treat the stitched parts equally [4]; methods that use each data set separately to estimate the similarity of candidates to the seed genes and then fuse similarity scores through weighting [5–8, 8–12]; and methods that construct gene correlation networks independently from each data set and find genes that are similar to the seed genes in the composite network [13–17].

These approaches are limited to data that *explicitly* refer to genes. They cannot readily treat data that are relevant for gene prioritization but are provided in a non-gene data space, such as disease ontologies, phenotype classifications, drug interactions and annotations of small chemicals. A labor-intensive approach to consider data from non-gene space is feature engineering, which transforms circumstantial data into gene profiles. However, feature engineering is neither standardized nor effortless and is a bottleneck that prevents the implementation of truly large-scale data fusion for gene prioritization. As an alternative to gene-centric approaches, Collage represents a major advancement in (i) the breadth of data it can incorporate, (ii) the ease of data integration without complex feature engineering, (iii) the high prediction accuracy, (iv) the ability to retain the relational structure both within and between data sets during model inference and (v) the capacity to incorporate knowledge of data structure in model design.

We used Collage to solve a problem in an exciting and relatively new field of interest − the use of *Dictyostelium* as a model system to explore the interaction between eukaryotes and prokaryotes. *D. discoideum* is a free-living soil amoeba that feeds on bacteria. The amoebae eat both Gram-negative and Gram-positive bacteria, but they respond differently to bacteria from these two groups. Early studies have shown that mutations can impair the ability of the amoebae to grow on either Gram-positive or on Gram-negative bacteria [18]. Other studies have shown that the amoebae can serve as a model for the interaction between eukaryotes and prokaryotes, including pathogenesis [19–21]. This system is an important addition to the field because *Dictyostelium* is a very convenient model organism that offers a variety of experimental tools, including classical genetics and modern genomic approaches.

The interaction between *D. discoideum* and several Gram-positive and Gram-negative bacteria has recently been explored with genetic and genomic methods [22]. These studies revealed transcriptome-level responses to the two bacterial groups and discovered a handful of genes that are essential for growth of amoebae on bacteria. The genetic analysis suggested that one in a hundred of the 12,000 genes in the *D. discoideum* genome is required for bacterial discrimination [22]. Identifying and characterizing these genes is a laborious task that requires several months of work per gene. We hypothesized that Collage could simplify this task by prioritizing genes and suggesting which ones should be tested by direct experiments.

## Results

### Compressive data fusion

**Fig 1:**
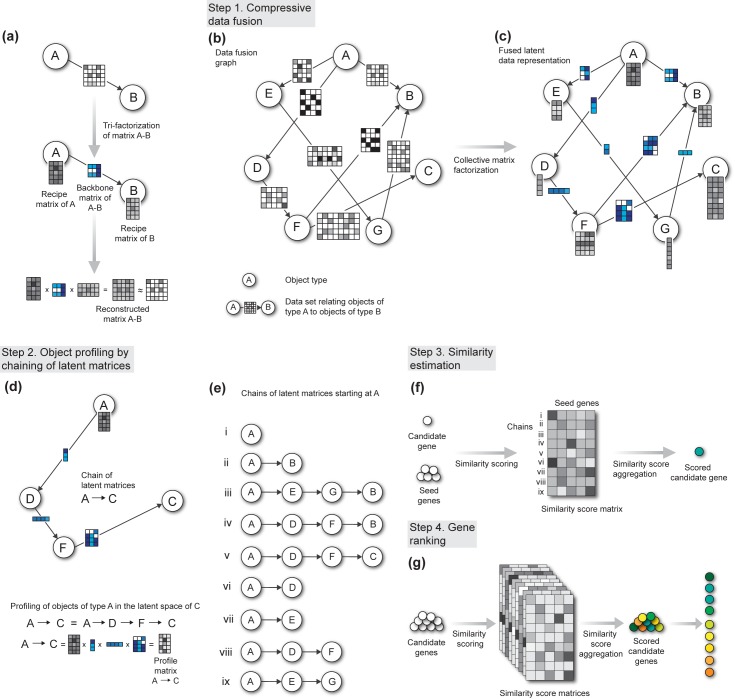
Overview of the Collage prioritization algorithm. **(a)** A data matrix in Collage relates two object types. We graphically represent this relation such that nodes A and B represent object types, and the directed edge A-B connects the two nodes with an associated data matrix. The matrix has objects of type A (e.g., genes) in the rows and objects of type B (e.g., experimental conditions) in the columns, as indicated by the edge directionality. Grey cells in the matrix represent quantitative measurements (e.g., mRNA transcript abundance), or binary memberships that relate objects in rows to objects in columns. Empty cells denote missing values. **(a, bottom)**. To model this relation, tri-factorization decomposes the data matrix into three smaller, low-dimensional latent matrices, whose product should well reconstruct the original matrix. Two latent “recipe matrices” map objects A and B into the latent space, and the remaining “backbone matrix” describes the relations in the latent space. In essence, the backbone matrix is a compressed version of the original data matrix. **(b)** Collage collectively models many data matrices that share object types. We organize the matrices in a data fusion graph. Object types are denoted as nodes (A to G), which may correspond to genes, ontology terms, diseases and patients, etc. **(c)** Instead of separately tri-factorizing each data matrix, Collage collectively factorizes all the matrices to a set of backbone matrices (edges, matrices in blue, one for each original data matrix) and recipe matrices (nodes, one for each object type), where the recipe matrices are shared across data sets that report on a common object type. **(d)** Collage chains latent matrices of the resulting factorized model to profile target objects (e.g., genes) in the latent space of any other object type. For example, the profiling of objects A in the latent space C is constructed by chaining that starts at node A and traverses the graph to node C through D and F. **(d, bottom)** Chaining multiplies the recipe matrix A by the backbone matrices along the traversed path. **(e)** The A-to-C path in **(d)** is one of nine chains through which we can profile objects A in our exemplar data fusion graph. **(f)** The number of profile vectors for each object of type A corresponds to the number of chains. Collage compares the profiles of candidate genes to the profiles of the seed genes. Given a candidate gene, Collage records its rank correlation-based similarities in a similarity score matrix with seed genes in the columns and chained profiles in the rows. The final score estimates the similarity of a candidate to a set of seed genes and is obtained by summarizing the similarity score matrix with a single value (green circle) computed by a median-based L-estimator. **(g)** The similarity score of a gene is a proxy for its degree of involvement in the phenotype characterized by the set of seed genes. Hence the prioritization is defined by ranking the candidates according to their seed-similarity scores.

Collage starts with a collection of data sets and can consider any kind of information (data tables, ontologies, associations, networks) that can be encoded in a matrix (S2 Fig). Each data set is viewed as a relation between two object types. For example, gene expression data relate gene names (columns) to experimental conditions (rows), where the entries represent transcript abundance. Literature annotation data relate research papers and their contents to annotation terms, where the entries are Boolean. Such data sets are abundant in the field of molecular biology and they report on dyadic relations that can be encoded in matrices. Matrix data representation is suitable for a wide range of data types, including tables, associations, ontologies and networks (S1 Fig). Whenever data sets share object types, we can connect them in a data fusion graph with object types as nodes and data matrices as edges. In the simplest data fusion graph shown in Fig 1a (top), node A may represent known genes in a certain genome and node B may denote various experimental conditions. A gene from A could be related to an experimental condition in B through a level of its mRNA abundance. Relationships between all genes and experimental conditions are represented in a data matrix that is placed on the edge A-B.

We model the system of data sets (Fig 1b) through data fusion by collective matrix factorization [23] (see also the tutorial provided in the S1 Text). Matrix factorization compresses the data matrices to a latent space and infers recipes to convert the latent representation back to the original data domain. Each data matrix is decomposed into a product of three low-dimensional latent matrices (S1 Fig): a “backbone matrix” encodes the relations between the latent components, and two “recipe matrices” transform the backbone matrix to the original space of the object types (Fig 1a). Data sets that are directly related and share a node in the fusion graph report on a common object type and hence use a common recipe matrix in their decomposition. Importantly, decomposition of any data set in the system depends on all other data sets according to a design of the fusion graph (Fig 1c). Sharing of recipe matrices ensures data fusion and allows Collage to incorporate knowledge about the relations between data sets.

### Chaining of latent matrices

Collage profiles objects in the latent space of any other object type based on the connectivity in the data fusion graph. In the simplest scenario, where object types are adjacent, such as A and D in Fig 1c, Collage profiles objects of type A in the latent space of D by multiplying the recipe matrix of A by the backbone matrix A-D. The resulting profile matrix has objects of type A in rows and the latent components of type D in columns. The advantage of Collage over other gene prioritization tools is its ability to profile objects whose types are not direct neighbors in the fusion graph, such as A and C in Fig 1c. To profile objects of A in the latent space of C Collage starts with the recipe matrix of A and multiplies it by backbone matrices A-D, D-F and F-C on the path from A to C (Fig 1d). If A represented genes, D literature, F literature annotations and C chemical compounds, this procedure would yield profiles of genes in the latent space of chemical compounds. We refer to this technique as latent matrix chaining. It constructs dense profiles that include the most informative features obtained by collectively compressing data via matrix factorization. Intuitively, chaining is able to establish links between genes and chemical compounds even though relationships between these object types are not available in input data in Fig 1b.

### Gene prioritization

Collage prioritizes objects of the target object type (e.g., genes, node A in Fig 1) based on a small set of seed objects (previously characterized genes). For each target object, it constructs a set of profile matrices by considering all possible chains of latent matrices that start in the target node and end in any node that is reachable in data fusion graph (Fig 1e). A profile matrix corresponds to a particular latent matrix chain and encodes the latent space of the chain’s last node. Each profile matrix is used to estimate the similarity between any two targets (genes) by comparing their respective profiles. Collages estimates the overall similarity between a candidate gene and the seed genes by aggregating similarity scores of the candidate gene across all profile matrices (Fig 1f). As a final step, Collage ranks all the genes based on their overall similarity with the seed genes (Fig 1g).

### Bacterial response gene prioritization in *Dictyostelium*

**Fig 2:**
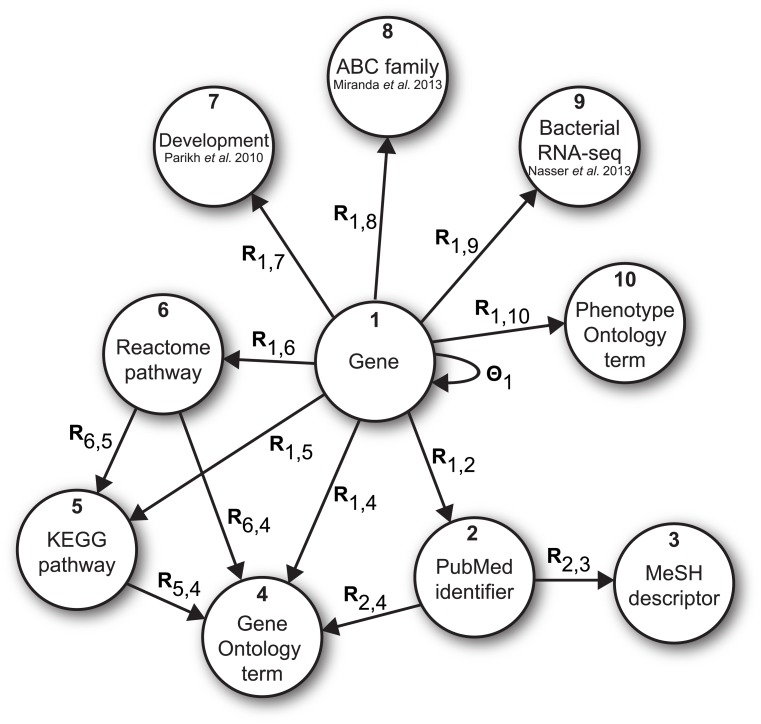
Data fusion graph for bacterial response gene prioritization in *Dictyostelium*. The graph shows the configuration of data sets, wherein nodes correspond to object types and edges denote data sets, each describing a relation between objects of two types. Collage considered 14 data sets (edges, represented by arrows) in this study describing the relations between 10 object types (nodes, represented by circles). The data sets included three whole-genome *D. discoideum* RNA-seq experiments [22, 24, 25] (**R**_1,7_, **R**_1,8_, **R**_1,9_), protein-protein interactions from the STRING database [26] (**Θ**_1_), gene mentions in research articles (**R**_1,2_) and their Medical Subject Headings (MeSH) annotations (**R**_2,3_), pathway memberships from the Kyoto Encyclopedia of Genes and Genomes (KEGG) [27] and Reactome [28] databases (**R**_1,6_, **R**_1,5_, **R**_6,5_), associations of genes to phenotypes from Phenotype Ontology [29] (**R**_1,10_), gene functions in Gene Ontology [30] (**R**_1,4_) and interrelatedness of Reactome and KEGG pathways and research literature with Gene Ontology terms (**R**_6,4_, **R**_5,4_, **R**_2,4_). See S4 Table for a detailed overview of considered data sets.

Collage is agnostic to data types it can consider and can be applied to any collection of data sets and any phenotype of interest. We used Collage to find genes that affect *D. discoideum* growth on the Gram-negative bacteria *Klebsiella pneumoniae*. We started with four seed genes that have been previously identified in a genetic screen for *D. discoideum* mutants that fail to grow on Gram-negative bacteria (Table 1). We fused 14 publicly available data sets that were considered relevant to the problem. Collectively, these data sets describe relations between 10 object types (see data fusion graph in Fig 2). Our prioritization task was particularly challenging since there is not a lot of information about *Dictyostelium* in the literature and in public databases and only one of the data sets (Fig 2, Bacterial RNA-seq, node 9) was directly related to bacterial response in *Dictyostelium*. Furthermore, the four seed genes, which were available to us at the beginning of this study, differ substantially in their data representation across data sets (S7 Fig). Collage ranked ∼ 12,000 genes from the *Dictyostelium* genome (S1 Table). The prioritized gene list was then filtered by the reported availability of *D. discoideum* gene knockout strains in the Dicty Stock Center (http://dictybase.org/StockCenter/StockCenter.html). We selected eight genes listed in Table 2 from the 30 top-ranked candidates (S2 and S3 Tables) for direct testing.

**Table 1:** Seed *D. discoideum* genes used for Gram-negative bacterial response gene prioritization. Seed genes used for prioritization by Collage were selected based on the experiments published in [22].

Gene	DictyBase ID	Description
*nip7*	DDBG0295477	Ortholog of the conserved NIP7 nucleolar protein that is required for 60S ribosome subunit biogenesis; contains a PUA domain.
*clkB*	DDBG0278487	Similar to the cell division cycle 2-related protein kinase 7 (CRK7) and other cell division cycle 2-like protein kinases; belongs to the CMGC group of protein kinases.
*spc3*	DDBG0290851	Ortholog of the conserved microsomal signal peptidase 23 kDa subunit; the signal peptidase complex is a membrane-bound endoproteinase that removes signal peptides from nascent proteins as they are translocated into the lumen of the endoplasmic reticulum; contains a putative signal peptide.
*alyL*	DDBG0286229	Amoeba lysozyme family protein (aly), but divergent compared to *alyA-D*.

**Table 2:** Top-ranked candidate *D. discoideum* genes tested for Gram-negative bacterial response. The name of the candidate gene, its identifier and description from DictyBase are shown, together with the rank (out of all *D. discoideum* gene knockout strains available in the Dicty Stock Center) at which the candidate was prioritized. Collage prioritized genes by fusing data sets from the fusion graph shown in Fig 2.

Gene	DictyBase ID	Description	Rank position
*cf50-1*	DDBG0273175	Component of the counting factor complex, which includes CF60, CF50, CF45-1, and CtnA (countin).	1
*smlA*	DDBG0287587	Cytosolic protein present in vegetative and developing cells.	2
*acbA*	DDBG0270658	Precursor of SDF-2; similar to diazepam binding inhibitor; enriched in prespore cells.	3
*abpC*	DDBG0269100	120 kDa F-actin binding protein also often called filamin; involved in actin cytoskeleton organization, motility, sand development; enriched in prestalk cells.	6
*pikB*	DDBG0283081	Phosphatidylinositol kinase.	9
*pikA*	DDBG0278727	Phosphatidylinositol kinase.	11
*pten*	DDBG0286557	Phosphatase and tensin homolog.	15
*modA*	DDBG0269154	Protein post-translational modification mutant.	23

### Validation of top ranked candidate genes

**Fig 3:**
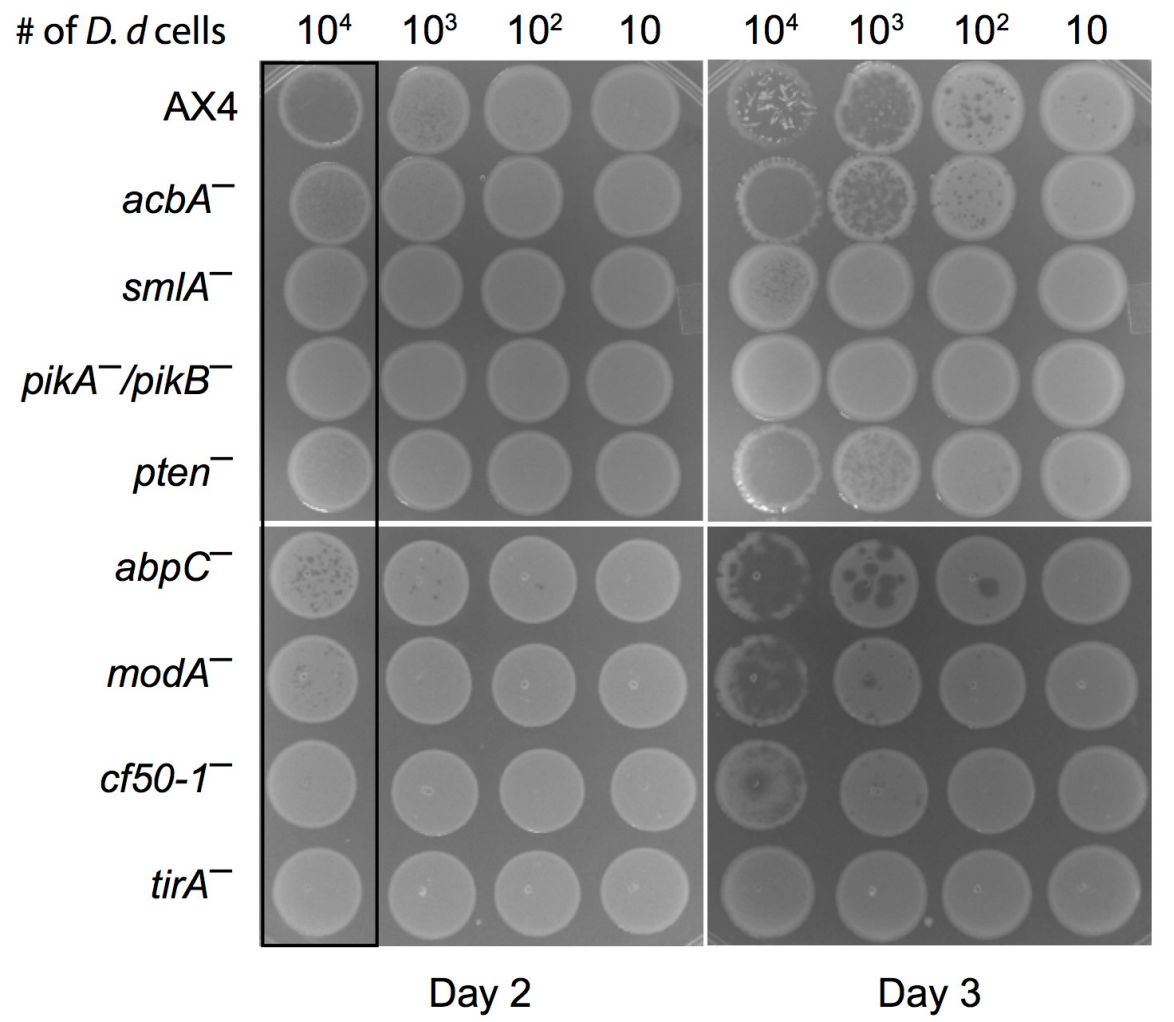
Experimental validation of top ranked candidate genes. Co-cultures of *D. discoideum* (*D.d.*) and bacteria were generated by serial dilutions of axenically grown *D. discoideum* amoebae with a large excess of *K. pneumoniae* bacteria such that the number of amoebae plated in each spot was between 10 and 10^4^ as indicated above each column. The relevant genotypes of the amoebae strains are indicated on the left of each row. The co-cultures were plated on SM agar plates and incubated in a humid chamber at 22°C. Images were taken at 2 and 3 days after plating to show the progression of amoebae growth in time. The larger white opaque spots are lawns of the *K. pneumoniae* bacteria. Growth of the *D. discoideum* amoebae results in the formation of plaques within the opaque spots in cases of low amoebae cell density or clearing of much or all of the opaque spots in cases of high amoebae cell density. Upon complete clearing of the bacteria, the amoebae starve and begin to develop, producing white protruding multicellular structures within the lawn (e.g. AX4, 10^4^ cells, day 3), which have no significance in this assay. Growth of the Collage-predicted knockout strains was compared to the wild type (AX4, top row) and to the most severe mutant available (*tirA–*, bottom row). Each experiment was performed in duplicate. Representative images of three independent experiments are shown.

To validate the selected candidate genes, we assessed growth of the *D. discoideum* knockout strains by making serial dilutions of the amoebae and co-culturing the cells with *K. pneumoniae* bacteria on nutrient agar. We observed a significant difference in the growth of all the mutants compared to the wild type AX4 (Fig 3). In this system, the bacteria grow faster than the amoebae so the first observation is the appearance of a thick opaque lawn of bacteria on the surface of the agar plate within 24 hours (not shown). Later on, as the amoebae eat the bacteria, they clear parts or all of the bacterial lawn, depending on their density and growth rate. When there are numerous, fast growing amoebae, we observe a cleared lawn (e.g. Fig 3, AX4, 10^4^ cells, Day 2). When there are very few amoebae, we observe distinct plaques that appear as darker spots in the bacterial lawn (e.g. Fig 3, AX4 Day 3, 10^2^ cells). When the bacteria are consumed, the amoebae starve, aggregate, and form developmental structures (Fig 3, AX4 Day 3, 10^4^ cells). Cells that carry an inactivating mutation in the *tirA* gene (*tirA^−^* cells) exhibit impaired growth on *K. pneumoniae* [31]. We used these cells as a control in our assay and indeed they exhibited no clearing of the bacterial lawn when plated at the same initial density as the wild type cells (Fig 3, AX4 vs. *tirA^−^*, Day 2, 10^4^ cells). We note that *tirA^−^* cells can grow to some extent on *K. pneumoniae* bacteria under certain conditions, indicating that the growth phenotype is continuous even though many researchers tend to describe it as Boolean.

We tested the predictions made by Collage on eight genes−*acbA*, *smlA*, *pikA*, *pikB*, *pten*, *abpC*, *modA* and *cf50-1* (Table 2). In the case of *pikA* and *pikB* we used a double knockout strain because of previously reported overlap in the functions of these two genes [32]. Strikingly, when we assessed the ability of the mutant cells to grow on bacteria, they all exhibited varying degrees of growth defects compared to the equivalent wild type (AX4) control (Fig 3). Comparing only one condition, disruption of *acbA*, *abpC* and *modA* resulted in small individual plaques in the bacterial lawn but not complete clearing as observed in AX4 (Fig 3, black box, Day 2, 10^4^ cells). In contrast, mutations in *smlA*, *pikA/pikB*, *pten*, and *cf50-1* caused phenotypes as severe as the loss of *tirA* with no clearing on Day 2 (Fig 3, black box, Day 2, 10^4^ cells). Further distinction in the ability to grow on bacteria was revealed when the mutant cells were observed for an additional day. For example on Day 2, *pikA^−^/pikB^−^* and *pten^−^* cells exhibited similar growth defects, but by Day 3, the loss of *pten* did not hinder growth on bacteria as much as the loss of *pikA* and *pikB* (Fig 3).

The seed genes we selected are required for growth on Gram-negative bacteria but dispensable for growth on Gram-positive bacteria [22]. This information was not included explicitly in our Collage analysis, but it was interesting to test the effect of the eight validated genes on growth on Gram-positive bacteria as well. We therefore plated the mutant strains on *Bacillus subtilis* bacteria and tested their growth. The wild type (AX4) control grew well, as did the *tirA* mutant, thus validating the assay. Disruption of *acbA*, *smlA*, *pten*, *abpC* and *modA* had no discernible effect on growth on *Bacillus subtilis* but mutations in *pikA/pikB* and in *cf50-1* caused severe growth defects that were comparable to those seen on *K. pneumoniae*.

## Discussion

The results indicate that Collage is capable of prioritizing genes in a reliable manner and identifying genes with various effects on the tested phenotype. This allows the analysis of a broad spectrum of genes in a given biological pathway. Application of the method to this specific question required only a few days of computational work and the validation step required a few more days of work. Considering the low yield of standard genetic screens, it would have taken about a year to identify eight new genes in the bacterial response pathway.

Three out of the five validated bacterial growth genes–*abpC*, *smlA* and *pten*, are involved in actin polymerization and cell motility [33–36]. One explanation for the enrichment of these genes is that the availability of preexisting knockout strains may be enriched with cell motility genes. This is because *D. discoideum* has been used extensively as a model system for chemotaxis, and many genes involved in cell motility have been disrupted and made available to the community. Nonetheless, the importance of actin in the consumption of bacteria may have been previously oversimplified, and the enrichment of these genes could be due to an essential role for actin in bacterial consumption. Proper regulation of actin is required for cell motility, phagocytosis and intracellular trafficking of phagosomes to lysosomes [33–36]. Each of these processes could be important in hunting, consuming and digesting bacteria.

We identified the sugar modifying alpha-glucosidase II enzyme, ModA [37]. Complex sugar modifications are important for biogenesis and intracellular trafficking of proteins. Others have shown that disruption of *modA* results in a lack of anionic N-glycan, which is associated with lysosomal enzymes [38]. While it may not be surprising to identify genes that regulate actin and lysosomes in a direct genetic screen, it is important to see that Collage did so too (S8 Fig).

We also identified one gene, *acbA*, with a less salient relationship to bacterial consumption. Gene *acbA* encodes an Acyl-CoA Binding protein, which is similar to the mammalian diazepam binding inhibitor. Acyl-CoA Binding protein is secreted during *D. discoideum* development and cleaved to form the SDF-2 peptide (Spore Differentiation Factor-2) [39, 40]. The role of Acyl-CoA Binding protein and SDF-2 in growth on bacteria is unclear. It is unlikely to be due to disruption of a general cellular growth pathway, since *acbA^−^* cells grow normally in axenic medium and it is unclear whether the SDF-2 peptide is secreted during growth because the system that produces it is developmentally-regulated. The identification of *acbA* suggests that novel gene functions can be discovered with our gene prioritization method.

The ranking of candidate genes depends on a particular collection of data sets we consider for gene prioritization. Removal of data sets from the data fusion graph (S3 Fig) changes the prioritization. When fewer data sets are considered, the validated genes from our study become ranked lower, below the top 30 (S3 Table). This is an intuitive dependence, less information should result in reduced prioritization accuracy, which we validated by simulations (S9 Fig). For every considered data compendium, Collage achieved a higher area under the ROC (AUC) statistic for known bacterial response genes than for randomly selected genes. However, not surprisingly, the suitability of a data compendium to rank genes depended on the number of data points in the compendium as well as on the usefulness of individual data sets. Our previous computational studies in data fusion with collective matrix factorization bear additional evidence that exclusion of data sets gradually reduces the quality of the predictions [41, 42]. We can attribute our success in identification of genes that participate in Gram-negative response pathways to the proposed approach and the appropriate choice of 14 relevant data sets. In the absence of a much larger set of known genes for this pathway, we cannot claim that this particular selection of data sets is optimal.

Collage builds upon our recently developed data fusion method by collective matrix factorization [23], and extends it with post-processing by latent matrix chaining and gene profiling. Collective matrix factorization has already provided accurate predictions of gene functions in *Dictyostelium* and yeast [43] and drug toxicity in mouse and human [42], where the accuracy was higher than that of other methods including random forests and approaches based on multiple kernel learning. Another utility of collective matrix factorization was also found in the study of disease interactions [41]. In these studies, collective learning enabled excellent accuracy and effortless integration of a range of very diverse data sets. Collective learning hence provides means for Collage to constitute a useful complement to large-scale ranking of genes in various organisms and to ranking of other objects contained in the fusion graph, such as drugs, diseases and pathways.

Our previous experiments with collective matrix factorization demonstrate that collective matrix factorization applies to diverse range of data sets, learning tasks and organisms. Through latent matrix chaining, Collage adapts collective factorization to prioritization, and thus Collage inherits the general applicability and robustness of collective factorization. Rather than in part extending our previous *in silico* studies we here report on the ability of Collage to make novel and highly accurate predictions.

## Materials and Methods

### Data sets

A total of 14 data sets and 10 object types were considered for Gram-negative bacterial response gene prioritization. Data sets were organized in a data fusion graph (Fig 2). We used RPKM-normalized RNA-seq transcriptional profiles of 35 abc-transporter mutant strains and wild-type AX4 strain in two biological replicates and at four different time points during development [24] (**R**_1,8_), normalized gene expression profiles analyzed by RNA-seq and measured at 4-hour intervals during the 24-hour development of *D. discoideum* in two biological replicates [25] (**R**_1,7_), and normalized abundances of gene transcripts in two replicates and four different bacterial growth conditions analyzed with RNA-seq [22] (**R**_1,9_). We also included the following publicly available data sets: Phenotype Ontology [29] annotations (**R**_1,10_) downloaded from the DictyBase data portal in March 2014, protein-protein interactions from the STRING v.9 database [26] (**Θ**_1_), membership of *D. discoideum* genes in pathways from the Reactome database [28] (**R**_1,6_) downloaded in August 2013, Kyoto Encyclopedia of genes and genomes (KEGG) pathway memberships [27] (**R**_1,5_), and annotations of genes in Gene Ontology [30] (**R**_1,4_). Additionally, we cross-referenced Reactome and KEGG pathways (**R**_6,5_), Gene Ontology terms and Reactome pathways (**R**_6,4_), and KEGG orthology groups and Gene Ontology terms (**R**_5,4_). Literature data included associations of genes to research articles from PubMed (**R**_1,2_) accessed in August 2013 through DictyBase, mapping of research articles to Gene Ontology terms (**R**_2,4_) and their Medical Subject Headings (MeSH) (**R**_2,3_). As a final step before data analysis, we normalized all relation data matrices such that the Frobenius norm of every row profile was equal to one. S4 Table summarizes the number of objects of each type and the data sets considered in our analysis.

### Data fusion by collective matrix factorization

A total of 14 data sets and 10 object types were considered for Gram-negative bacterial response gene prioritization (Fig 2). Data sets are viewed as dyadic relations and are encoded in relation and constraint matrices. A relation matrix **R**_*i*,*j*_ is a *n*_*i*_ × *n*_*j*_ real-valued matrix, in which rows correspond to objects of type *i*, columns to objects of type *j* and the element **R**_*i*,*j*_(*k*, *l*) represents the relationship between objects *k* and *l*. A constraint matrix **Θ**_*i*_ is a *n*_*i*_ × *n*_*i*_ matrix that relates objects of type *i* to themselves. It contains pairwise constraints indicating the dissimilarity/similarity between objects. Larger positive elements in **Θ**_*i*_ direct data fusion algorithm to infer a latent model in which the corresponding objects have fewer similar latent profiles (i.e., positive elements in the constraint matrices specify cannot-link constraints). Larger negative elements indicate greater similarity of latent profiles (i.e., negative elements in the constraint matrices specify must-link constraints). Constraint matrices are used for regularization and are not factorized (S4 Fig). Given a collection of relation matrices 𝓡 (**R**_*i*,*j*_ for different choices of *i* and *j*) and a collection of constraint matrices 𝓒 (${\text{Θ}\;}_{i}^{\left(l\right)}$ for different choices of *i*, where *l* enumerates constraint matrices available for object type *i*), collective matrix factorization simultaneously decomposes all the relation matrices in 𝓡 while regularizing the inferred latent model with the constraints in 𝓒. This is accomplished by minimizing our previously proposed loss function [23]:

$$\begin{array}{c} \min_{G_{i}\geq 0,S_{i,j}}\sum_{R_{i,j}\in R}{∥R_{i,j}-G_{i}S_{i,j}G_{j}^{T}∥}_{\text{Fro}}^{2}+\sum_{\Theta_{i}\in C}\sum_{l=1}^{l_{i}}\text{tr}\left(G_{i}^{T}\Theta_{i}^{\left(l\right)}G_{i}\right). \end{array}$$

The objective function aims at good reconstruction of the observed elements in the data matrices and penalizes violated constraints. The inferred low-dimensional matrix factors **G**_*i*_ and **S**_*i*,*j*_ form decompositions of the relation matrices such that ${\text{R}}_{i,j}\approx {\text{G}}_{i}{\text{S}}_{i,j}{\text{G}}_{j}^{T}$ for all *i* and *j*. Here, **G**_*i*_ is a *n*_*i*_ × *c*_*i*_ nonnegative latent matrix (a “recipe matrix”) containing latent profiles of objects of type *i* in the rows, **G**_*j*_ is a *n*_*j*_ × *c*_*j*_ nonnegative latent matrix with profiles of objects of type *j* in the rows, and **S**_*i*,*j*_ is a *c*_*i*_ × *c*_*j*_ latent matrix (a “backbone matrix”) that models interactions between latent components in the (*i*, *j*)-th data set. Latent profile of an object of type *i* is given by its corresponding row vector in **G**_*i*_ and encodes membership of the object to *c*_*i*_ latent components.

The key principle of data fusion is sharing of latent matrices among decompositions of related matrices. Latent matrix **G**_*i*_ is utilized for decomposition of any relation matrix that describes objects of type *i*, that is, **G**_*i*_ is used in factorizations of matrices **R**_*i*,*j*_ and **R**_*j*,*i*_ for any object type *j*. While latent matrix **G**_*i*_ is shared, latent matrix **S**_*i*,*j*_ is specific to the relation **R**_*i*,*j*_. The inferred latent model thus consists of object type-specific latent matrices (**G**_*i*_) and latent matrices specific to individual data sets (**S**_*i*,*j*_).

The algorithm for inference of fused latent models is accompanied by previously reported proofs of correctness and convergence [23]. Briefly, it is an iterative algorithm that starts by randomly initializing latent matrices **G**_*i*_ and then alternates between updating matrices **G**_*i*_ and **S**_*i*,*j*_ until convergence. To ensure robust prioritization, the algorithm was run 20 times with different initializations of latent matrices. The algorithm was run for a maximum of 200 iterations or was terminated early if the total reconstruction error between consecutive iterations changed by less than 0.01. Parameters of the algorithm are factorization ranks, *c*_*i*_, for every object type *i* in the data fusion system. Our prioritization of *D. discoideum* genes included 10 types of objects; we have selected latent dimensionality of object types through a single parameter representing the fraction of the original data dimensionality such that (*c*_1_, *c*_2_, …, *c*_10_) = (*kn*_1_, *kn*_2_, …, *kn*_10_). The value of *k* was obtained by observing kinks in a diagram of total reconstruction error when varying *k* from 0.05 to 0.5. We selected *k* = 0.1 where a maximum kink was attained. S5 Fig summarizes the procedure and the resulting latent data dimensionality of each object type used in our analysis.

### Gene profiling by chaining of latent data matrices

We assembled gene profiles by relying on the latent data matrices inferred by collective matrix factorization. Each gene was characterized through a collection of profiles determined by the topology of data fusion graph. Collage constructed gene profiles by starting at the gene node and its corresponding recipe matrix (**G**_1_). The method traversed along edges of data fusion graph and multiplied the edge-associated backbone matrices. In the bacterial response gene prioritization study there were 15 chains of latent matrices (Fig 2), and consequently 15 distinct profile matrices containing gene profiles of every considered gene: **G**_1_, **G**_1_ **S**_1,7_, **G**_1_ **S**_1,8_, **G**_1_ **S**_1,9_, **G**_1_ **S**_1,10_, **G**_1_ **S**_1,2_, **G**_1_ **S**_1,6_, **G**_1_ **S**_1,5_, **G**_1_ **S**_1,4_, **G**_1_ **S**_1,2_ **S**_2,3_, **G**_1_ **S**_1,6_ **S**_6,5_, **G**_1_ **S**_1,6_ **S**_6,4_, **G**_1_ **S**_1,2_ **S**_2,4_, **G**_1_ **S**_1,5_ **S**_5,4_ and **G**_1_ **S**_1,6_ **S**_6,5_ **S**_5,4_. It should be noted that latent matrix chains may vary in length and that precise number of chains including a particular backbone matrix is decided by the structure of data fusion graph. For example, in our study, the backbone matrix **S**_1,6_ was contained in four chains whereas matrix **S**_2,3_ participated in a single chain. Since each resulting profile matrix is determined by a path through object types, adding further away object types increases the weight of intermediate backbone matrices. It therefore can occur that matrices (i.e., data sets), which are present in many chains, have greater influence on prioritization than matrices (i.e., data sets), which appear in fewer chains. However, we would like to note that an intermediate backbone matrix with large latent dimensionality does not necessarily dominate construction of the profile matrix as can be seen from similarity score matrices in S6 Fig. Because Collage operates on matrix chains, it gives a natural approach for incorporating relevance of data sets. Collage assumes that a more relevant object type is the object type that is closer to target type (e.g., genes) in terms of the number of links needed to connect it with the target node. Consequently, in gene prioritization, this means that data sets, which are closely related to genes might have a stronger effect on prioritization than distant non-gene related data sets.

### Gene prioritization

The inputs to gene prioritization were candidate genes, seed genes and the set of profile matrices. Collage aims to find genes whose profiles are similar to the profiles of seed genes. The approach estimates the similarities independently for each profile matrix, and then aggregates the resulting scores to obtain the final prioritization. Each row in a profile matrix corresponds to a profile of a gene. Collage assesses similarity between a candidate gene and a seed gene by computing Spearman rank correlation of two respective row vectors. In this study, this procedure yielded a 15 × ∣seed genes∣ similarity score matrix of rank correlations for each candidate gene (S6 Fig). Similarity score matrices are aggregated in a two-step median value computation along score matrix dimensions to produce a single rank value per gene. Collage reports on empirical P-values obtained by randomizing seed set of genes. Randomization of seed genes was repeated 500 times. A nominal P-value of a candidate rank was estimated as (*h*+1)/(*n*+1), where *n* is the number of replicate seed sets that have been simulated and *h* is the number of these replicates that produced aggregated score greater than or equal to that calculated for the actual seed set.

As a gene profile similarity measure, Collage uses Spearman rank correlation due to the correspondence of rank correlation with assignments of genes to the latent components of inferred matrices. A promising candidate gene should have a latent profile similar to the profile of a seed gene. Given a profile matrix **X**, candidate gene *g* and seed gene *s*, gene *g* is considered promising if its latent component with the largest membership is the same as that of seed gene *s*. We formalize this intuition by measuring whether arg max_*j*_ **X**(*g*, *j*) = arg max_*j*_ **X**(*s*, *j*). The same should hold for the latent component of the second largest, third largest, and all remaining value-ordered gene memberships. Quantitatively, the described procedure corresponds to rank correlations between candidate and seed genes.

The implementation of Collage for bacterial response gene prioritization in *Dictyostelium* is available online (http://github.com/marinkaz/collage). Readers are invited to browse, use and contribute to the software.

### Generalization performance of Collage on data subcompendia

To study the sensitivity of gene prioritization to the number of data sets in the data fusion graph, we observed how the rankings of the validated candidate genes changed when the overall prioritization was obtained by fusing different subsets of data sets from our initial collection. In addition to the original model that contained 14 data sets we applied Collage to four independent gene prioritization data scenarios (S3 Table). The scenarios considered seven, four, three and two data sets, where each model was applied to a different subset of entire data collection (S3 Fig). The selection of data sets was in part determined by the data fusion graph. In particular, for data fusion to take place, the associated graph has to be connected such that information can be shared between data matrices. To evaluate the usefulness of Collage to fuse data matrices in a non-gene data space we performed leave-one-out cross-validation. In each validation run, one seed gene was excluded from a set of seed genes and added to test set consisting of *D. discoideum* genes whose knockout strains were available in the Dicty Stock Center. Collage then determined the ranking of this gene for each data scenario separately. From the overall prioritization on a given data compendium, we calculated sensitivity and specificity values of Collage and reported the receiver operating characteristic (ROC) curve and the AUC statistic based on ranks of left-out genes (S9 Fig). As a negative control for prioritization, we applied Collage to randomly selected seed sets of genes using all considered data sets.

### Experimental analysis of *Dictyostelium* mutants

*D. discoideum* strains were obtained from the Dicty Stock Center and grown axenically in HL-5 at 22°C [22]. *K. pneumoniae* was maintained in SM broth at 22°C. To assess the ability of *D. discoideum* to grow on bacteria, *D. discoideum* cells were collected from axenic cultures during logarithmic growth and washed once with Sorensen’s buffer [22]. *D. discoideum* cells were serially diluted with bacteria (OD_600_ = 1.0) and spotted onto SM agar plates. The plates were incubated in a humid chamber at 22°C, and images of plates were taken every 24 hours.

## Supporting Information

S1 Text A friendly tutorial to Collage. The tutorial provides a step-by-step explanation of the mathematical and computational concepts considered by Collage.
(PDF)

S1 Fig A schematic overview of matrix tri-factorization. The figure illustrates the decomposition of the *m* × *n* gene-to-phenotype data matrix **R** into a product of three low-rank latent matrices, **F**, **S** and **G**. The goal of tri-factorization is to approximate the large-scale gene-to-phenotype matrix with a product of much smaller latent matrices such that the approximation is as good as possible. The original *m* × *n* gene-to-phenotype data matrix **R** is compressed by factorization into a much smaller *c*_1_ × *c*_2_ matrix **S** of latent (meta) genes in rows and latent (meta) phenotypes in columns. Matrix **S** is asymmetric and models the interactions between the latent components. To map this compressed representation back to the original domain space, we need two additional matrices, **F** and **G**. The *m* × *c*_1_ nonnegative matrix **F** maps the space of the meta genes to the space of genes. In each of the *m* rows, matrix **F** contains the memberships of a respective gene in each of the *c*_1_ latent components (meta genes). Similarly, each column of the *c*_2_ × *n* nonnegative matrix **G** contains the memberships of a respective phenotype in each of the *c*_2_ latent components (meta phenotypes).
(PDF)

S2 Fig Representation of information sources with data matrices. Matrices in Collage describe relationships between objects of two types. Matrix rows correspond to objects of one type, columns correspond to objects of the other type and matrix elements express the degree of a relationship between the corresponding objects. The figure illustrates matrix representation of six distinct data sets. **(a)** Degrees of protein-protein interactions from the STRING database are represented in a gene-to-gene matrix. **(b)** Membership of genes in pathways are represented in binary matrices, one column for each pathway. Binary matrices are also used to associate **(c)** pathways with gene ontology terms and **(d)** research articles with Medical Subject Headings. **(e)** The structure of Gene Ontology can be represented with a real- valued matrix, whose elements report on distance or semantic similarity between the corresponding ontological terms. **(f)** Levels of gene expression, an experimental data set, are represented by a matrix of stacked gene expression profile vectors.
(PDF)

S3 Fig Data fusion graphs for the study of model sensitivity to data set selection. Besides the full collection of data sets (data fusion graph in Fig 2), we have considered data collections with a smaller number of data matrices and studied the impact of this reduction on gene prioritization (S3 Table). We ran gene prioritization analyses by considering subsets of **(a)** seven, **(b)** four, **(c)** three and **(d)** two data sets that were included in our original study.
(PDF)

S4 Fig A schematic overview of penalized matrix tri-factorization. A prominent approach to approximate a matrix with a system of latent matrices is singular value decomposition (SVD). Factorized models inferred by SVD are prone to overfitting, they cannot guarantee conservation of the desired structural properties of the latent matrices, such as nonnegativity, and they are hard to interpret. These shortcomings of SVD and its variants have spurred the development of regularized learning approaches to matrix factorization. Penalized matrix tri-factorization introduces regularization to tri-factorized latent model. In the figure, the input data matrix is accompanied by two constraint matrices that express degrees of similarity between genes (matrix in yellow and orange) or phenotypes (matrix in blue and green). Constraint matrices guide the inference of latent matrices. In our implementation, elements of constraint matrices that have greater negative values represent must-link constraints, i.e., the corresponding genes (or phenotypes) should have more similar latent profiles. Elements with positive values have the opposite effect—they represent cannot-link constraints by penalizing the latent data model if the corresponding genes (or phenotypes) have similar latent profiles. The matrix factorization algorithm balances between good approximation and adherence to the constraints.
(PDF)

S5 FigReconstruction error as a function of factorization rank.Collective matrix factorization requires specification of latent data dimensionality, that is, a factorization rank for each modeled object type. Factorization rank determines the degree of compression of relation matrices: compression is higher with latent matrices of lower dimensionality. The study of bacterial response gene prioritization in *D. discoideum* considered data sets describing relationships between objects of 10 different types. Factorization ranks were set through a single parameter *k*, where the factorization rank was set to *kn*_*i*_ for each object type *i* with *n*_*i*_ objects. The value of *k* was selected by observing the change of the “total reconstruction error” (black line), $\sum_{{\text{R}}_{i,j}\in 𝓡}\|{\text{R}}_{i,j}-{\hat{\text{R}}}_{i,j}{\|}_{\text{Fro}}$, when varying *k* between 0.05 and 0.5 (x-axis, “fraction of original data dimensionality”). The reconstruction error was estimated by 50 repetitions of collective matrix factorization, where each repetition was run with a different random initialization of latent matrices. The bars show reconstruction errors of individual data matrices, $\left\|{\text{R}}_{i,j}-{\hat{\text{R}}}_{i,j}\right\|$ (“relation reconstruction error”). See Fig 2 in the main text for description of the data matrices. We selected *k* = 0.1 where the maximum kink was attained. This choice resulted in latent data dimensionality (*c*_1_, *c*_2_, *c*_3_, *c*_4_, *c*_5_, *c*_6_, *c*_7_, *c*_8_, *c*_9_, *c*_10_ = (1287, 342, 280, 308, 9, 9, 5, 28, 5, 50) with a limitation on minimum factorization rank set to 5.(PDF)

S6 Fig Visualization of similarity score matrices for candidate genes validated in the wet laboratory. Collage profiles genes through chaining of latent matrices. For a given candidate gene, the profiling procedure yields as many gene profiles (i.e., data vectors corresponding the gene) as there are different chains of latent matrices. Collage then assesses similarity between the candidate gene and a particular seed gene by computing Spearman rank correlation between the respective gene profiles. The figure shows the resulting 15 × 4 (i.e., there were 15 chains and 4 seed genes in our study) similarity score matrix containing rank correlations for each candidate *Dictyostelium* gene that was validated in the wet laboratory.
(PDF)

S7 Fig Heterogeneity of seed genes. We assessed whether and to what degree the data on seed genes *spc3*, *clkB*, *nip7* and *alyL* that were considered for bacterial response prioritization in *Dictyostelium* vary across individual seed gene. To determine how a given seed gene is different from other seeds, we randomized seed set 400 times and in each randomization used Collage to calculate the similarity score between the given gene and the random set of seed genes (shown in light green). For a given gene we also show its true score as estimated by Collage (e.g., see the vertical line corresponding to the value for *spc3* in top left panel) when only the remaining three seed genes (e.g., *clkB*, *nip7* and *alyL* in top left panel) were considered for scoring. One possible explanation for the substantial amount of variation across seed genes is that these genes were previously identified to be involved in bacterial response pathways using various genetic and genomic methods [22]. They might therefore participate in different aspects of bacterial recognition. Large heterogeneity of seed genes also indicates the difficulty of prioritization task considered here and suggests that consideration of all four seed genes for prioritization is important.
(PDF)

S8 Fig Homogeneity of candidate genes validated in wet laboratory. We assessed how alike are candidate genes that were validated in wet laboratory on the basis of their latent data representation estimated by Collage. To determine how a given candidate gene is different from other candidates, we randomized set of candidates considered for experimental validation 400 times and in each randomization used Collage to calculate the similarity score between the given gene and the random set of genes (shown in light green). For a given gene we also show its true score as estimated by Collage (e.g., see the vertical line corresponding to the value for *cf50-1* in top left panel) when the remaining seven genes (e.g., *abpC*, *pikA*, *pten*, *modA*, *acbA*, *pikB* and *smlaA* in top left panel) were considered for scoring.
(PDF)

S9 Fig Generalization performance of Collage. To estimate generalization performance of Collage for bacterial response prioritization in *Dictyostelium*, we performed cross-validation on seed genes in order to obtain sensitivity and specificity of our model. For this task, the leave-one-out cross-validation fitted well. **(Left; a, b)** We applied Collage once for each seed gene using all other seed genes as training genes and the left-out gene as a test gene (positive control). For the negative controls, we considered genes, whose mutants are available in the Dicty Stock Center (727 genes from S2 Table). Shown are the **(a)** ROC curves with the area under the ROC curve statistics, and **(b)** precision-recall curves with the area under the precision-recall curve statistics based on ranks of left-out genes. The removal of non-gene related data matrices decreased sensitivity and specificity of Collage, suggesting the important ability of Collage to link non-gene related data matrices. The data sources used to construct every performance curve are indicated in S3 Fig. **(Right; a, b)** **(a)** Rank ROC curves and **(b)** precision-recall curves obtained for the leave-one-out cross-validation performed on eight top ranked candidate genes, which were used for testing Collage and proven to be involved in bacterial response pathways. Notice that higher accuracy of results shown in the right panel relative to results in the left panel was expected as the eight top ranked candidate genes have all been predicted using the same seed set (S8 Fig). We would hence like to warn readers about possible confounding effects present in the experiment whose results are shown in the right panel. In both figures, the control ROC curve (black dashed line) was obtained after prioritization with randomly constructed seed sets and by using all data sources.
(PDF)

S1 Table Whole-genome prioritization list. Prioritized list of *D. Dictyostelium* genes with the associated empirical P-values and the aggregated prioritization scores as estimated by Collage.
(XLSX)

S2 Table Gene prioritization list for a subset of genes from the DictyBase available in the Dicty Stock Center. Prioritized list of *D. Dictyostelium* genes with the associated empirical P-values and the aggregated prioritization scores as estimated by Collage. This is the sublist of S1 Table, where only genes that were available in the Dicty Stock Center for direct testing are included.
(XLSX)

S3 Table The impact of modeling circumstantial data on the overall *D. discoideum* bacterial response gene prioritization. The table lists the top-30 candidate genes obtained by prioritization by data fusion of 14, 7, 4, 3 and 2 data sets from the data fusion graphs in S3 Fig. Genes in bold are the ones selected for the experimental study.
(PDF)

S4 Table Summary of data sets considered for bacterial response gene prioritization in *D. discoideum*. The notation of the data sets (“Data matrix” column) is the same as in the data fusion graph (Fig 2). All relation data matrices were normalized before data analysis such that the Frobenius norm of every row profile was equal to 1. This type of data normalization was also considered in our previous studies with collective matrix factorization. Preprocessed data sets are provided with the project related code and are available from GitHub repository (http://github.com/marinkaz/collage).
(PDF)
