# Supplementary material for: Gene Prioritization by Compressive Data Fusion and Chaining
Source: PLoS Comput Biol. 2015 Oct 14;11(10):e1004552. doi: 10.1371/journal.pcbi.1004552 (PMC4605714; doi:10.1371/journal.pcbi.1004552)
Supplement: S1 Text — The tutorial provides a step-by-step explanation of the mathematical and computational concepts considered by Collage. (PDF) [file pcbi.1004552.s001.pdf]

Gene prioritization by compressive data fusion and chaining

A friendly tutorial to Collage

Supplementary note

Marinka Žitnik[1], Edward A. Nam[2,#], Christopher Dinh[3], Adam Kuspa[2,3],
Gad Shaulsky[2] & Blaž Zupan[1,2,*]

[1] *Faculty of Computer and Information Science, University of Ljubljana, Ljubljana, Slovenia*
[2] *Department of Molecular and Human Genetics, Baylor College of Medicine, Houston, TX, USA*
[3] *Department of Biochemistry and Molecular Biology, Baylor College of Medicine, Houston, TX, USA*

[#] Current address: University of St. Thomas, Houston, TX, USA
[*] blaz.zupan@fri.uni-lj.si

1 An overview

We propose an algorithm for gene prioritization that is able to collectively consider a large array of diverse data sets. Gene prioritization assumes that we are given a small set of seed genes and we are looking for other genes that would give a similar phenotype when mutated. A distinguishing feature of Collage, our gene prioritization algorithm, is its ability to carry out the search for candidate genes by incorporating large number of data sets that might be rather distantly related to the problem. For example, classification of diseases on its own does not directly provide information about genes, but may be useful if jointly considered with information on drug targets. Genes that are targeted with "similar" drugs may share a function. Humans are capable of inferring new associations that span different kinds of information, and our aim was to construct an algorithm that mimics this behavior.

Collage starts with a collection of data sets, each represented in a matrix. We begin our tutorial with an example of a matrix that associates genes and functions (Section 2). We proceed by explaining basic linear algebra operations, such as matrix multiplication, that are necessary for understanding the prioritization algorithm (Section 3). An essential part of the algorithm is matrix tri-factorization (Section 4) - an operation that considers a large input data matrix and compresses it to three smaller matrices. The product of the three matrices is a good approximation of the original matrix. In this way, the algorithm infers

essential patterns that govern the data. Most importantly, we have devised the method such that some small matrices (factors) are shared between the compressions of a set of input matrices (Section 5). Collage can handle large collections of diverse data sets from sources that may include tables, ontologies, networks and more, as long as they can be represented with matrices (Section 6). For the joint consideration, Collage organizes the matrices in a fusion graph (Section 7) and applies collective matrix tri-factorization (Section 8) to obtain a compact model of the data.

Collage profiles the genes with feature vectors that relate genes to any of the data sources included in the data fusion graph. Gene profiles are constructed by chaining of matrices (Section 9 and Section 10). Intuitively, matrix chaining may correspond to associative reasoning by which we, humans, can find relations between seemingly unrelated concepts. Genes with similar profiles assembled from the data fusion graph are assumed to have similar functions or to cause similar phenotypes when mutated. As the final step of our method, Collage ranks the genes according to their profile-based similarity with the seed genes (Section 11).

2 Matrix representation of a data set

Suppose we are given information about the functional annotation of a few genes. One way of representing these data is a matrix in which the rows correspond to genes and the columns represent gene functions. Collage would represent this hypothetical data set on five genes and six gene functions in a matrix as is done in Figure 1. In this example, the highlighted value 1 indicates that *fooB* is involved in phagocytosis and the highlighted value 0 indicates no known association of the gene *fgD* with the function 'regulation of phagocytosis'.

Gene Ontology terms

Genes	rRNA binding	Cell morphogenesis	Phagocytosis, recognition	Phagocytosis	Immune system process	Regulation of phagocytosis
zbljA	0	0	1	1	0	0
fooB	0	1	0	1	0	0
barC	0	1	1	0	0	0
fgD	1	0	0	1	1	0
hgE	0	1	1	1	0	1

Figure 1 A matrix of gene annotations.

The following concept is key to understanding our system. Collage considers this matrix as an edge in a special type of graph, which we call *a data fusion graph* (this name will make more sense when we add other data sets). In example in Figure 2, the edge describes relationships between two object types – genes and gene functions. Hence, the graph includes two nodes, one for each type of object, and an edge with an associated data matrix. The directionality of the edge indicates, which objects are in rows and which are in columns. In our example the edge points from “Genes” to “Gene Ontology terms” because the rows represent genes (*i.e. zbljA* and *fooB*) and the columns represent biological processes (*i.e.* rRNA binding and cell morphogenesis).

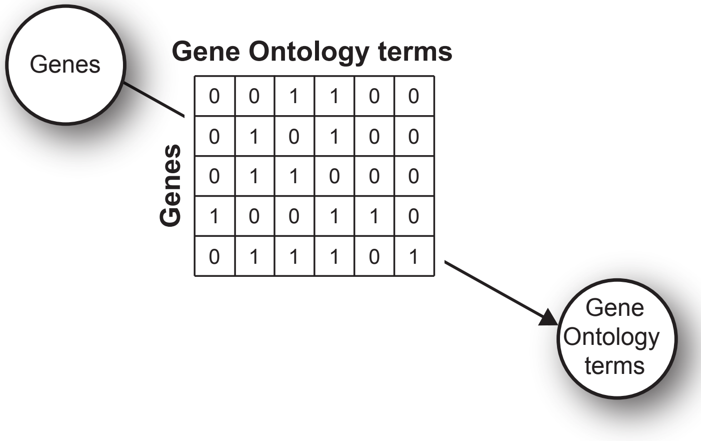

Figure 2 The simplest data fusion graph consists of a single data matrix.

3 A brief introduction to linear algebra operations

Matrix multiplication and transposition are two fundamental mathematical operations. Let us consider the two matrices in Figure 3, $\mathbf{A}$ is a 2 x 3 matrix and $\mathbf{B}$ is a 3 x 3 matrix. If we multiply them, we will get matrix $\mathbf{P}$, which is a 2 x 3 matrix. To populate the upper left element in matrix $\mathbf{P}$, we have to multiply the elements in the first row of matrix $\mathbf{A}$ by the elements of the first column of matrix $\mathbf{B}$ in the order they appear and add them up: 3 x 1 + 2 x 1 + 1 x 0 = 5. The middle upper element in $\mathbf{P}$ is the sum of the products of the elements in the first row of matrix $\mathbf{A}$ by the elements of the second column of matrix $\mathbf{B}$ in the order they appear: 3 x (-1) + 2 x 0 + 1 x 2 = -1. The right upper element in $\mathbf{P}$ is the sum of the products of the elements in the first row of matrix $\mathbf{A}$ by the elements of the third column of matrix $\mathbf{B}$ in the order they appear: 3 x 4 + 2 x 3 + 1 x 5 = 23. Likewise, the bottom row of matrix $\mathbf{P}$ will be populated by multiplying the elements in the second row of matrix $\mathbf{A}$ by the respective elements in the three columns of matrix $\mathbf{B}$.

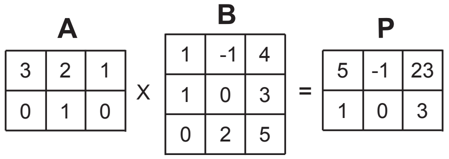

Figure 3 Matrix multiplication.

A more formal way to describe what we did is the following. Matrix multiplication takes two matrices as its input, an $n \times k$ matrix $\mathbf{A}$ and a $k \times m$ matrix $\mathbf{B}$ and outputs their product, which is an $n \times m$ matrix $\mathbf{P}$. Matrices $\mathbf{A}$ and $\mathbf{B}$ can be multiplied only if they match in their inner dimension, k. In that case the resulting matrix $\mathbf{P} = \mathbf{AB}$ has the same number of rows as $\mathbf{A}$ and the same number of columns as $\mathbf{B}$. The element in the ith row and the jth column of $\mathbf{P}$ is computed as the dot product of the ith row in $\mathbf{A}$ and the jth column in $\mathbf{B}$, $\mathbf{P}_{ij} = \sum_{c=1}^{k} \mathbf{A}_{ic}\mathbf{B}_{cj}$.

Matrix multiplication is associative. If three matrices $\mathbf{A}$, $\mathbf{B}$ and $\mathbf{D}$ are respectively $n \times k$, $k \times s$ and $s \times m$ matrices, we obtain their product $\mathbf{ABD}$ by grouping them in any of the two ways without changing their order, $\mathbf{ABD} = (\mathbf{AB})\mathbf{D} = \mathbf{A}(\mathbf{BD})$.

Matrix transposition is a relatively simple operation that flips a matrix on its side. Transposition takes an $n \times m$ matrix $\mathbf{A}$ and outputs the transposed matrix $\mathbf{A}^T$, which has dimension $m \times n$ and whose elements are defined as $\mathbf{A}^T_{ij} = \mathbf{A}_{ji}$. One can see in the example in Figure 4 that the element in the second row and third colum of matrix $\mathbf{A}$, namely $\mathbf{A}_{23} = 0$, becomes the element in the third row and second column of the transposed matrix ($\mathbf{A}^T_{32} = \mathbf{A}_{23}$):

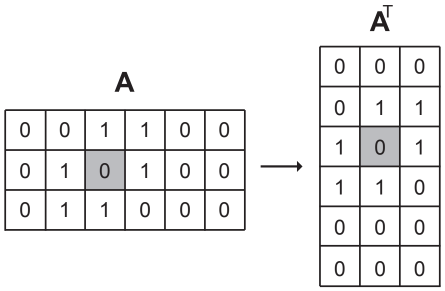

Figure 4 Matrix transposition.

4 Tri-factorization of a data matrix

The fundamental building block of Collage is matrix tri-factorization. Matrix tri-factorization assumes that there is some redundancy in the data, and that a large matrix can be ade-

quately represented as the product of three smaller matrices. From a biological perspective, we say that if two genes have similar properties (*i.e.* their respective rows in the data matrix are similar), then information about the first gene function might give us some insight into the function of the second gene.

The example in Figure 5 illustrates tri-factorization of the 5×6 gene-by-function data matrix we discussed above, converting it into three matrices: a 5×2 Gene recipe matrix, a 2×2 Backbone matrix and a 6×2 Function recipe matrix.

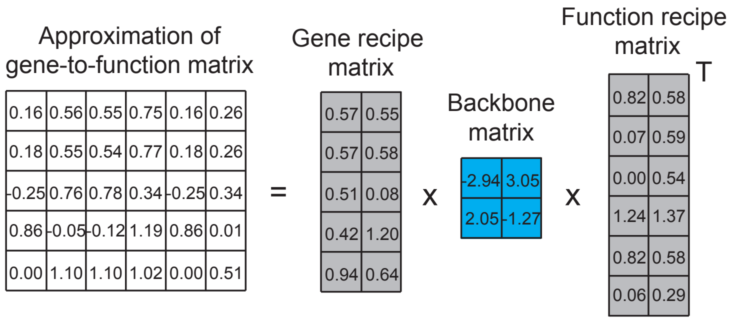

Figure 5 Tri-factorization of gene annotation data matrix.

We call the 2×2 Collage-inferred matrix (in blue) a *backbone matrix*, as it is a compressed version of the original matrix. In the backbone matrix, the rows correspond to “meta-genes”, and the columns to “meta functions”. Our backbone matrix models the interplay between meta genes and meta functions. To decompress the backbone matrix back to the original space, tri-factorization provides two other matrices (in grey) that we refer to as *recipe matrices*, as they provide a map from the compressed to the decompressed space. The product of the three matrices provides a good approximation of the original data set as is denoted by $\approx$ sign in Figure 6.

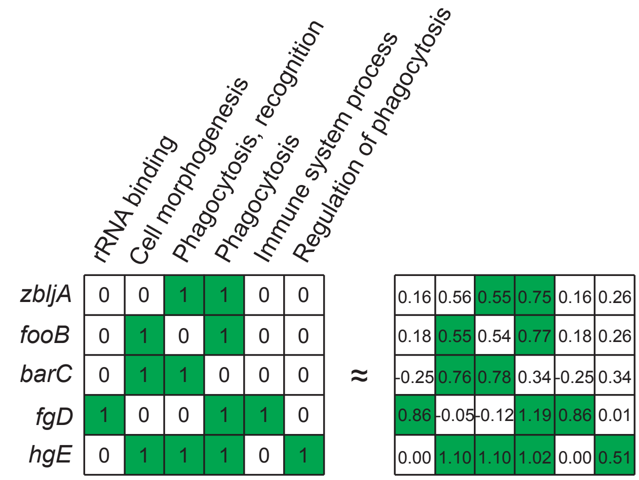

Figure 6 Reconstruction of gene annotation data matrix.

Figure 6 shows the original matrix on the left and its reconstruction as inferred by matrix tri-factorization on the right. In the reconstructed matrix we see that known gene annotations (elements in green that scored '1' in the original matrix) have substantially higher scores than the rest of the matrix elements, as should be expected of a well-performing factorization algorithm. This observation suggests that we did not lose a lot of information in the process. More importantly, many of the elements that contained '0' before have higher values now - a direct result of the inference process we performed. For example, our analysis suggests that *zbljA* might be a promising candidate gene for cell morphogenesis in our hypothetical example.

For the more mathematically oriented reader: matrix factorization is all about latent components, that is, patterns hidden in the original matrix but revealed in its compressed version. In our example we have two gene latent components represented by the columns of the gene recipe matrix and also two gene function latent components given in the columns of the gene function recipe matrix. Latent components should be understood as abstract groups of genes and groups of gene functions. Unlike our example, each object type can have a different number of latent components, that is, a different number of columns for its recipe matrix. The number of latent components is commonly referred to as the factorization rank and is a parameter of the Collage algorithm. Recipe matrices encode memberships of genes and gene functions, respectively, to latent components.

In the latent space, the gene-by-function backbone matrix thus relates latent components of genes and functions, elements that we have earlier referred to as meta-genes and meta-functions. For example, the backbone matrix cell of 3.05 (first row, second column in the blue matrix) indicates a high degree of similarity between the first gene latent component and the second gene function latent component.

The attentive reader may have noticed that gene function recipe matrix had to be transposed prior to the multiplication. Why this is so should become clear in the next section, but let us finish with a hint: latent factors can be reused when collectively factorizing a system of multiple matrices.

5 Collective tri-factorization of two data matrices

So far we have dealt with a single data matrix, the gene-by-function matrix. Data fusion, our core data integration algorithm, can simultaneously handle a much larger number of data matrices. For simplicity, let us consider the collective factorization of two data matrices, but the principle can be extended to a larger number. Suppose we have obtained additional information on the phenotypes of mutant strains in which the specific genes were inactivated and the phenotypes were recorded. We gathered these data in a matrix where the rows correspond to genes and the columns represent the mutant phenotypes as ontological concepts from Phenotype Ontology. As can be seen from the graphic below, *fooB*$^-$ exhibits decreased pinocytosis and *hgE*$^-$ has abolished culmination. We now have two matrices, our original 5 x 6 gene-by-function matrix and a new 5 x 4 gene-by-phenotype matrix as shown in Figure 7.

Gene Ontology terms

Genes	rRNA binding	Cell morphogenesis	Phagocytosis, recognition	Phagocytosis	Immune system process	Regulation of phagocytosis
zbljA	0	0	1	1	0	0
fooB	0	1	0	1	0	0
barC	0	1	1	0	0	0
fgD	1	0	0	1	1	0
hgE	0	1	1	1	0	1

Phenotype Ontology terms

Genes	Abolished culmination	Delayed aggregation	Increased cAMP binding	Decreased pinocytosis
zbljA	1	0	1	0
fooB	0	0	0	1
barC	1	0	0	0
fgD	0	1	0	0
hgE	1	0	1	1

Figure 7 Matrices of gene functions and mutant phenotypes.

Since we now consider three types of objects we add an additional "Phenotypes" node to our data fusion graph and a directed edge from "Genes" to "Phenotypes" to obtain the graph shown in Figure 8.

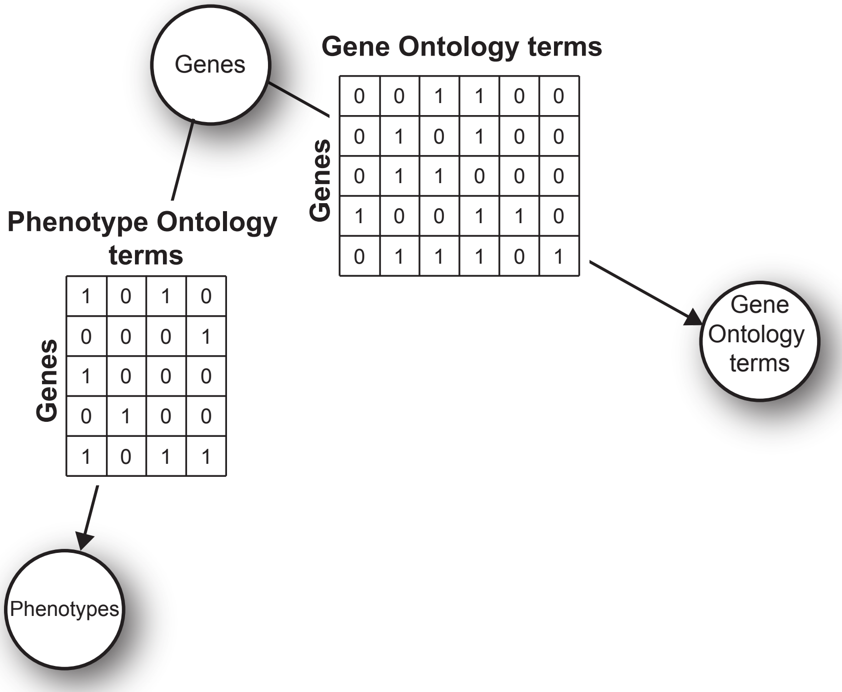

Figure 8 Data fusion graph shows the organization of two data matrices.

We now simultaneously decompose both data matrices as we did in Section 4 and we represent each data matrix as the product of three smaller matrices. In this case, the two data matrices share a common object type – the genes. We can therefore reuse the gene recipe matrix in both decompositions. This sharing of a recipe matrix is the cornerstone of our data fusion approach because it provides a mechanism to relate the information stored in heterogeneous data sets. Collage infers a gene recipe matrix (shown in grey in Figure 9) that is part of the tri-factorization of gene-by-function data matrix.

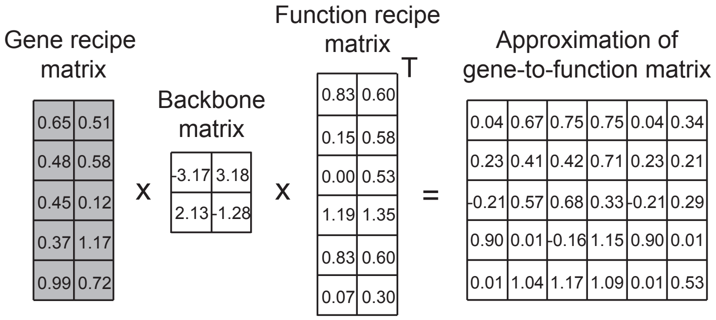

Figure 9 Collective factorization of gene function data matrix.
Highlighted is gene recipe matrix that gets shared among related data sets.

Importantly, the gene recipe matrix from Figure 9 is also part of tri-factorization of gene-by-phenotype matrix as can be seen from Figure 10.

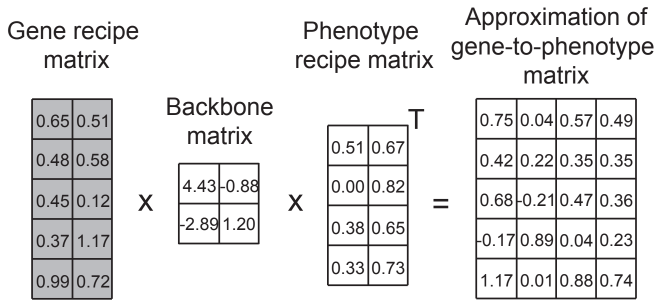

Figure 10 Factorization of mutant phenotype data matrix reuses the gene recipe matrix obtained by collective factorization of two data matrices.

Note that the decomposition of the gene-by-function matrix into the respective recipe and backbone matrices is not the same as in Section 4. This is because this decomposition was aimed at reconstructing both input matrices at the same time, whereas the task in the previous Section was concerned only with one data matrix. Using the two data matrices provided additional infomration that was utilized by Collage to improve its predictive ability.

As in Section 4 we can multiply the latent matrices and get a reconstructed gene-by-function data matrix (Figure 11)

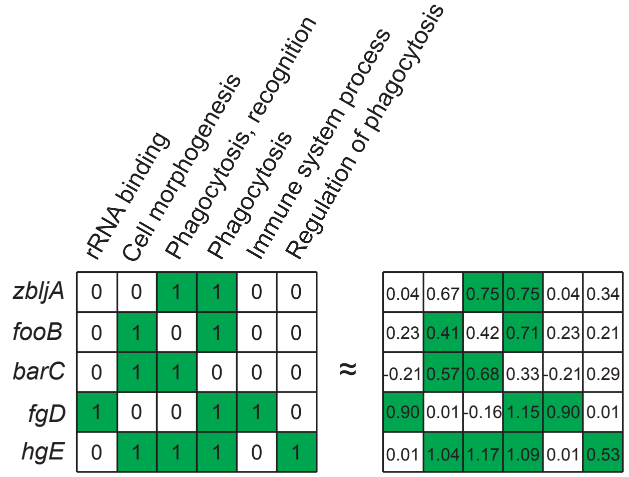

Figure 11 Reconstruction of gene function data matrix.

and a reconstructed gene-by-phenotype matrix (Figure 12).

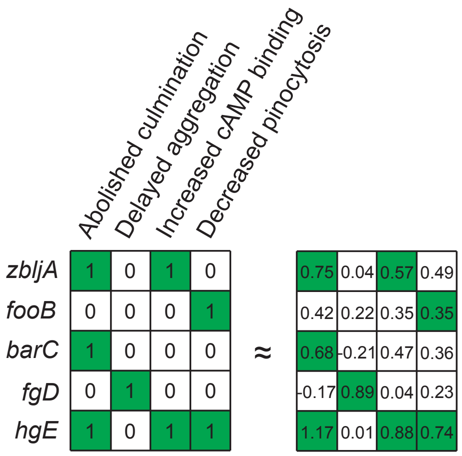

Figure 12 Reconstruction of mutant phenotype data matrix.

Importantly, since tri-factorization of the gene-by-function matrix was able to integrate additional information from the mutant phenotypes, it increased the confidence of *zbljA*, suggesting that it has a previously unknown function in cell morphogenesis. Its score was raised from 0.56 (first row, second column of reconstructed gene-by-function matrix) when only the gene-by-function data set was considered, to 0.67 when the factorization considered the gene-by-phenotype data as well.

Throughout this document we overlay Collage-inferred factorized matrices on the data fusion graph. In Figure 13 we place the backbone matrices on the edges, and the recipe matrices next to the nodes of the corresponding object types.

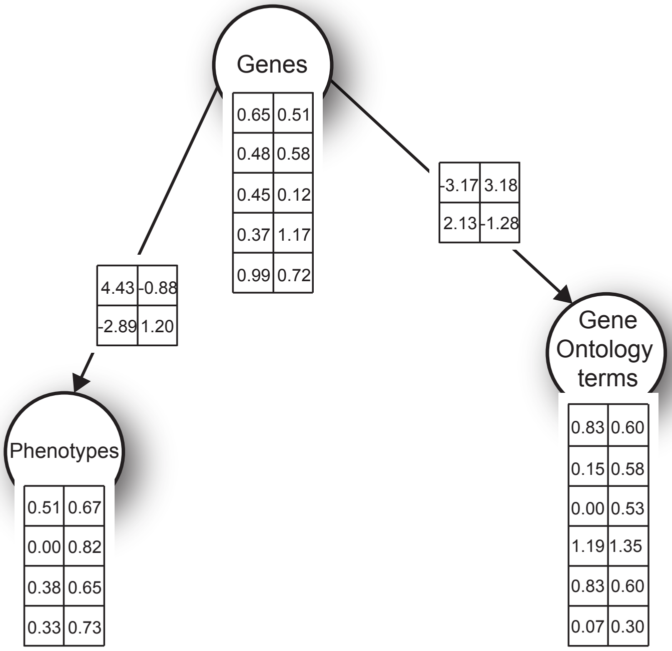

Figure 13 Data fusion graph with overlaid latent matrices inferred by Collage.

6 Data matrices for tables, ontologies and networks

We often have access to data that were generated by different technologies that captured relationships between objects from various aspects and at different levels of granularity. These data sets could be represented as ontologies, feature-based data tables, networks and associations, among others. However, almost all can be viewed as matrices describing dyadic relationships, that is, relationships between two object types. For example, gene-gene interactions might be given as a gene-by-gene matrix in Figure 14.

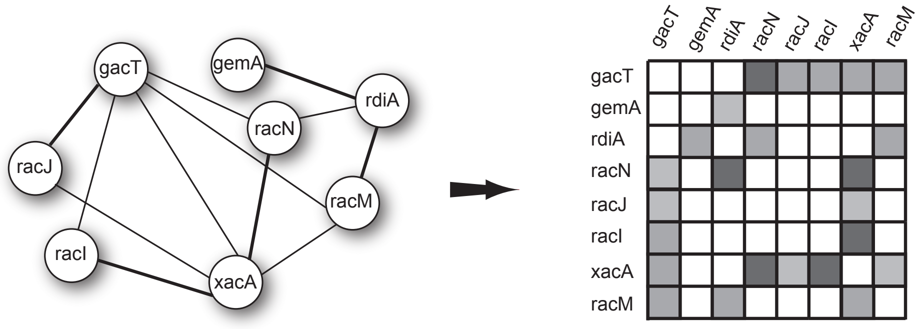

Figure 14 Gene-gene interaction data.

Shades of gray in Figure 14 denote the strength of the interactions. Note that some cells are white (empty), indicating no interaction has been observed for the corresponding gene pair, or the interaction has not been tested yet.

Participation of genes in biochemical and metabolic pathways can be arranged in a gene-by-pathway matrix (Figure 15). In this case, pathway participation is a binary relation, so this matrix is black-and-white, with no shades.

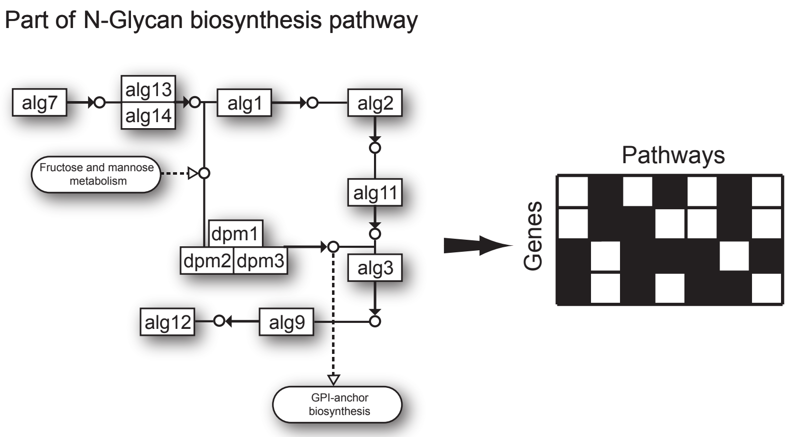

Figure 15 Molecular pathway data.

Finally, Figure 16 shows gene expressions measured at different time-points encoded in a gene-by-timepoint expression matrix.

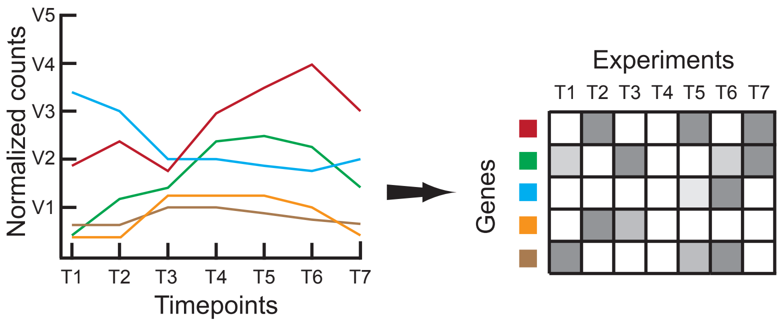

Figure 16 Gene expression measurements.

The data sets mentioned so far directly relate genes to some other type of objects. However, in biomedicine there is an abundance of circumstantial data sets that are only indirectly related to genes, but may still be useful for prioritization. For Collage, there is no

need to transform such data sets to a gene data space, thus avoiding the need for tedious and sometimes complicated data processing. An example of such data are annotations of Medical Subject Headings (MeSH) terms in published literature (Figure 17),

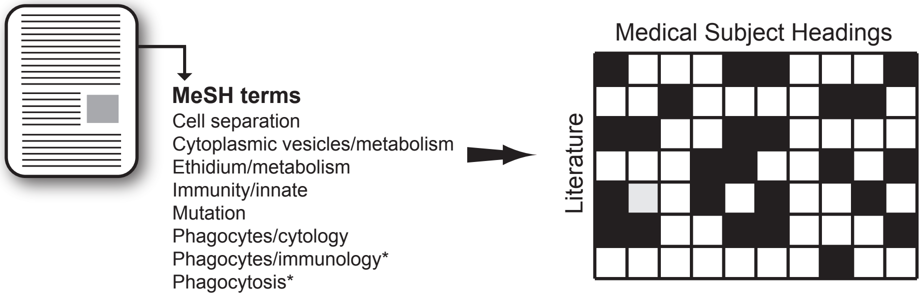

Figure 17 Annotations of research articles with terms from the Medical Subject Headings data base.

relatedness between Gene Ontology terms as given by the structure of ontological directed acyclic graph (Figure 18),

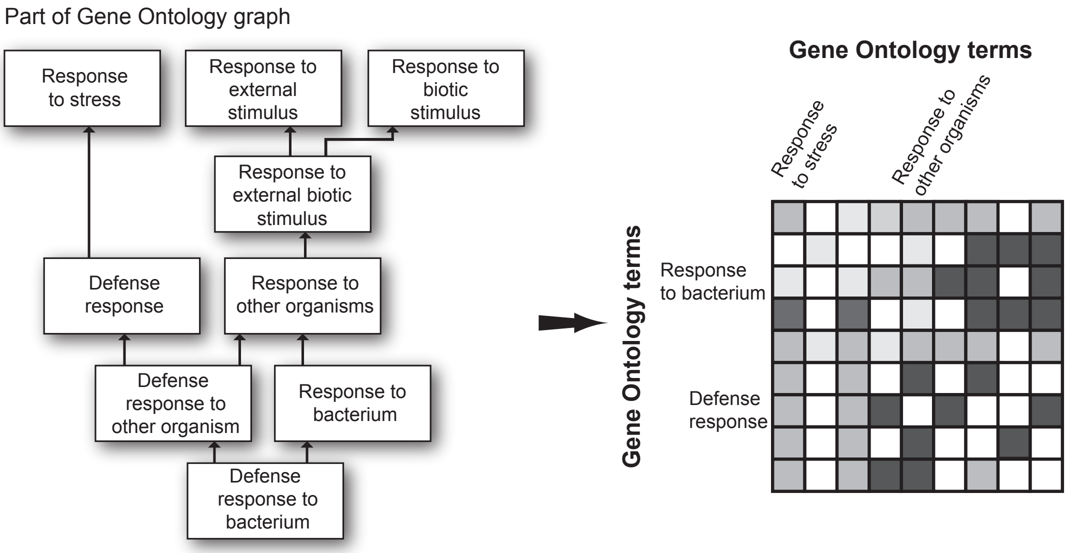

Figure 18 The connectivity of Gene Ontology graph.

and associations between metabolic pathways and Gene Ontology terms through the Kyoto Encyclopedia of Genes and Genomes (KEGG) orthology groups and Reactome database (Figure 19).

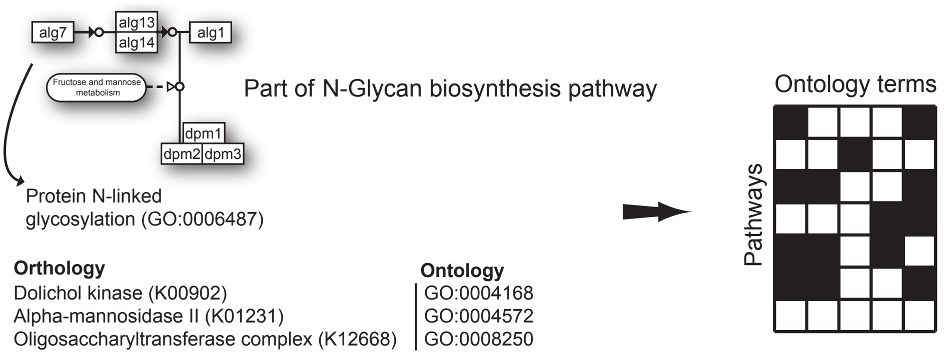

Figure 19 Relationships between metabolic pathways and gene functions.

With such an abundance of data sets, there is a need to organize the data in a structure that would help us comprehend the complexity of the problem domain and for Collage to provide information about their relatedness. We do so by constructing a data fusion graph.

7 Data fusion graph

As seen in Section 6, there are many potentially beneficial data sets that could contribute to improving the prediction strength of our test case. So far, in this tutorial, we modeled at most two data sets represented by gene-by-function and gene-by-phenotype matrices. We now continue with a more general data setup and include new data sets to demonstrate the full scope of Collage. For our test case, we assemble a data fusion graph consisting of 13 data sets and describing 8 types of objects and present it in Figure 20.

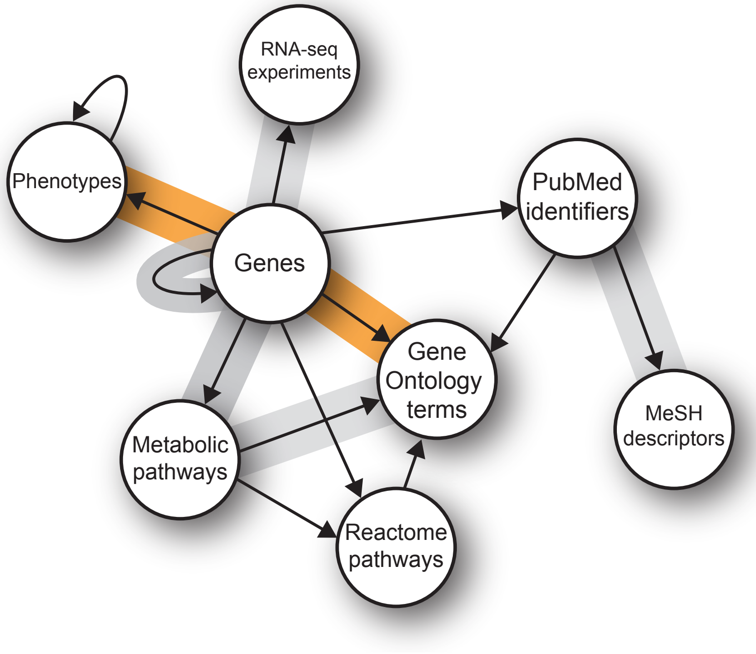

Figure 20 Data fusion graph with 13 data sets describing relationships between 8 types of objects.

The edges (data sets) shown in grey represent the data sets discussed in Section 6 and the edges in orange denote the data sets considered in Section 5. Additionally, we added data matrices of gene occurrences in the biomedical literature ("Genes" $\rightarrow$ "PubMed identifiers"), associations between research articles and Gene Ontology terms ("Pubmed identifiers" $\rightarrow$ "Gene Ontology terms"), metabolic links ("Genes" $\rightarrow$ "Reactome pathways", "Metabolic pathways" $\rightarrow$ "Reactome pathways"), cross-references of ontological terms and Reactome pathways ("Reactome pathways" $\rightarrow$ "Gene Ontology terms") and similarities between mutant phenotypes as defined by the topology of the Phenotype Ontology directed acyclic graph ("Phenotypes" $\rightarrow$ "Phenotypes").

Figure 21 shows the entire data fusion graph of our system with data matrices placed along the edges. Note that although the data were made up for this example, the fusion graph is rather complex and may come close to what we would use to solve a real biomedical problem. This particular data configuration is related to our bacterial response gene study in *Dictyostelium*.

Let us consider, for a moment, a few details of our data fusion graph. The "PubMed identifiers" node is associated with three edges. The data matrices on the two outgoing edges have the same number of rows (six rows) as they both relate research articles to other object types. On the other side, these research articles are given in the columns of the data matrix that is placed on the incoming edge from "Genes". As expected, the gene-

by-PubMed matrix also has six columns. Similar observations on the number of rows and columns apply to all nodes of the data fusion graph.

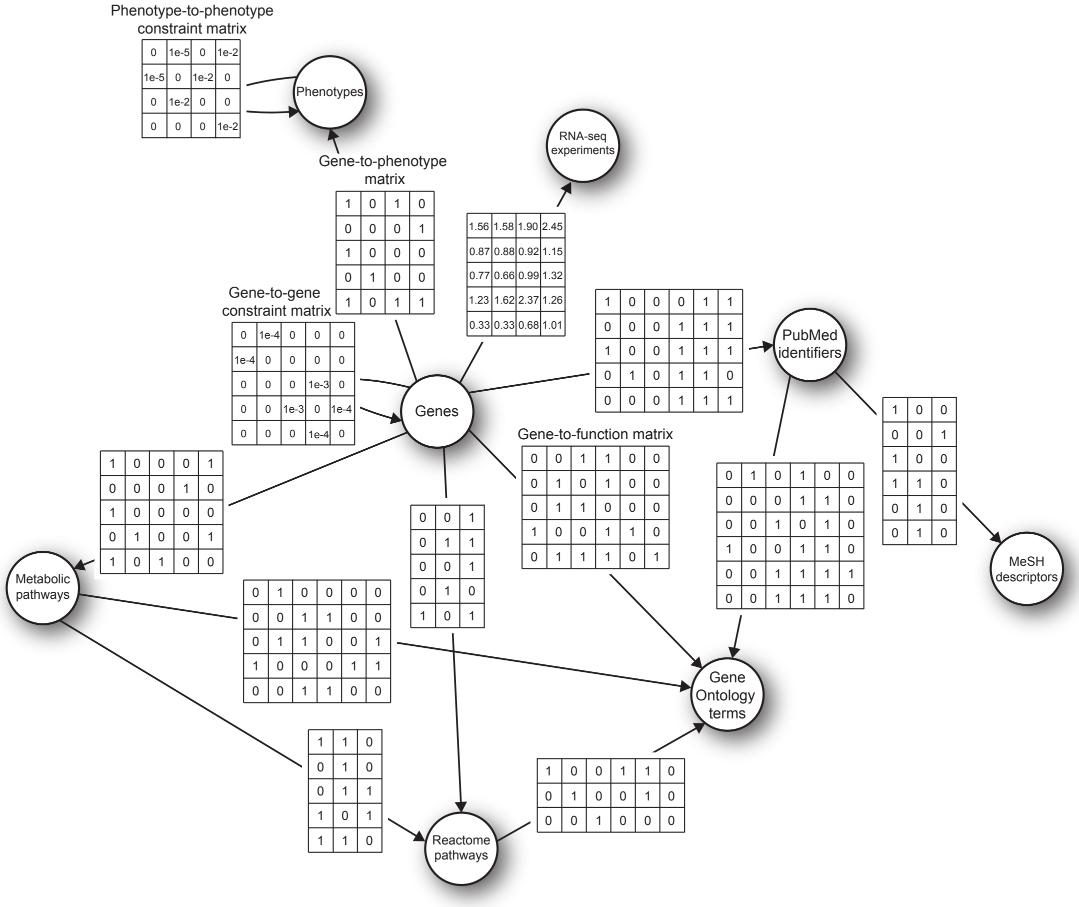

Figure 21 Fusion graph with overlaid data matrices.

8 Collective matrix tri-factorization

We now apply collective matrix tri-factorization, implemented in Collage, to simultaneously tri-factorize the data matrices in our system. Inferred matrices that approximate the original data sets are shown in Figure 22, where blue matrices denote the backbone matrices, *i.e.* compressed versions of the input data matrices, and the recipe matrices contain latent profiles of objects of different types.

There are two matrices in our system above (Figure 21), the phenotype-by-phenotype matrix and the gene-by-gene matrix of genetic interactions, for which the corresponding backbone matrices seem to be missing in the latent data representation shown in Figure 22.

These two matrices are “special” because they relate objects of the same type. Their purpose in Collage is to constrain the inference of phenotype and gene recipe matrices. Say, if we know that two genes interact somehow, then we would like their inferred rows in the gene recipe matrix to be more similar than if we have no knowledge about their interaction. On the contrary, if we know that two genes do not interact, we might reward the inference which would construct their recipe rows such that it shows less similarity. In our example, *zbljA* and *fooB* do not interact (very low interaction score of $1\mathrm{e}{-4}$, see first row and second column of the gene-by-gene matrix in Figure 21) and this piece of evidence rewards Collage’s to infer recipe rows that are more dissimilar than the average interaction. Indeed, *zbljA* and *fooB* have quite different memberships in the latent components ($[0.80, 0.84]$ versus the values $[0.57, 0.48]$ found in the first rows of the gene recipe matrix in Figure 22). Therefore, matrices, such as gene-gene interaction matrix in Figure 21, that relate objects of the same type are called *constraint matrices.*

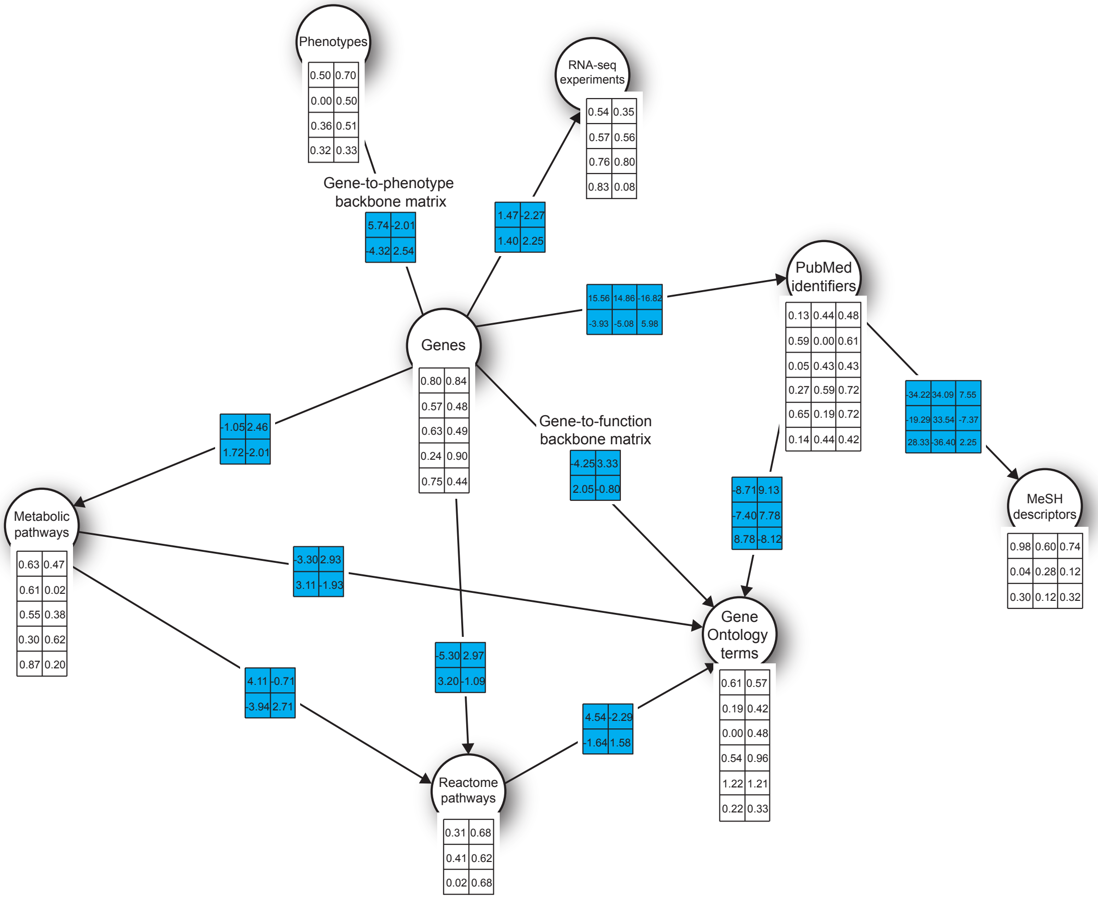

Figure 22 Collage jointly estimates latent representation of entire compendium.

The interested reader might now multiply the recipe matrices and backbone matrices shown in the figure below as we did in Section 5. Their products should match closely to the corresponding original data matrices. Data fusion is about compression, but it also tries to infer a model that well captures the input data sets.

9 Gene profiling

Until now we have been concerned with the Collage inference of a compressed system of data matrices. We will use this system to construct gene profiles that will be used for prioritization. We first present *chaining*, a procedure that allows us to characterize genes in the latent component space of any other type of objects that is included in the data fusion graph. That is, we propose an algorithm that can profile genes in the space of metabolic pathways, Gene Ontology terms, MeSH descriptors and others types of objects from the data fusion graph.

Let us say that we are interested in relating genes to MeSH descriptors and would like to construct the corresponding gene profiles (*i.e.* one vector per gene). A data set that would directly relate genes to MeSH descriptors does not exist, however, in our data fusion graph genes are related to MeSH descriptors indirectly through research literature (Figure 23).

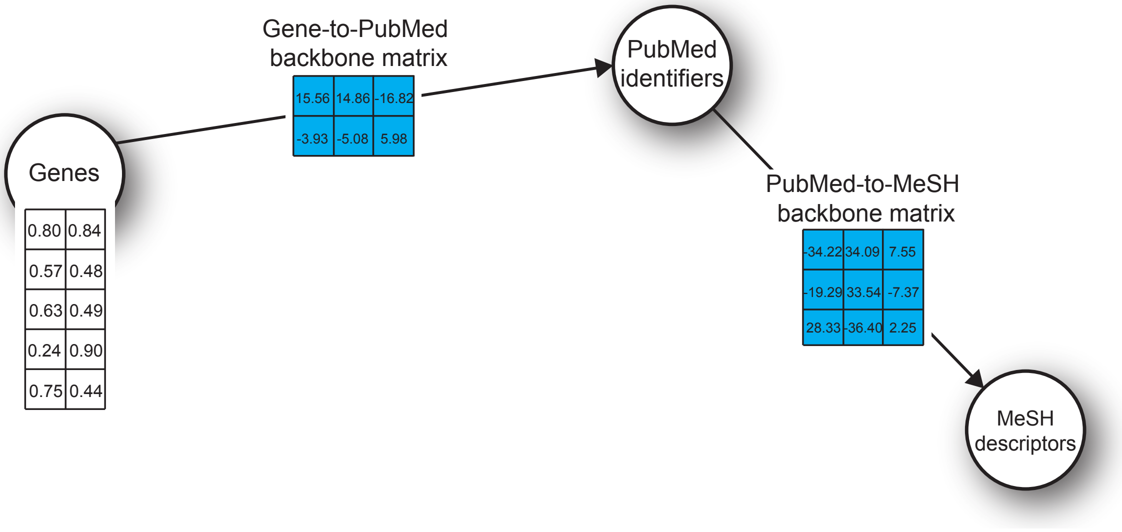

Figure 23 Part of data fusion graph that relates genes to the latent space of Medical Subject Heading descriptors.

Hence, we formulate a chain consisting of gene recipe matrix and two backbone matrices, gene-by-PubMed and PubMed-by-MeSH backbone matrices, and form a gene profile

matrix by multiplying the matrices along the chain (Figure 24). Notice that the chain in Figure 23 does not contain the recipe matrix corresponding to MeSH terms. This means that our compiled profile matrix profiles gene in the latent MeSH term space rather than in the original MeSH term space. The resulting matrix therefore has genes in rows and latent components of the MeSH space in columns. This way, we are later able to effectively compute similarities between candidate genes and seed genes in the smooth and condensed latent space, which has far fewer dimensions than original data.

There are, however, tasks other than gene prioritization, where exact association strengths between objects from the start and the end of a chain are important. For example, if predicting gene-disease associations, where diseases are represented with a respective part of the MeSH tree, we might want to know, which diseases are most likely associated with a particular gene instead of which groups of diseases (*i.e.* latent components of diseases) are connected with the gene. In such a scenario we would use latent data representation of Collage and assemble gene profiles in the decompressed MeSH term space.

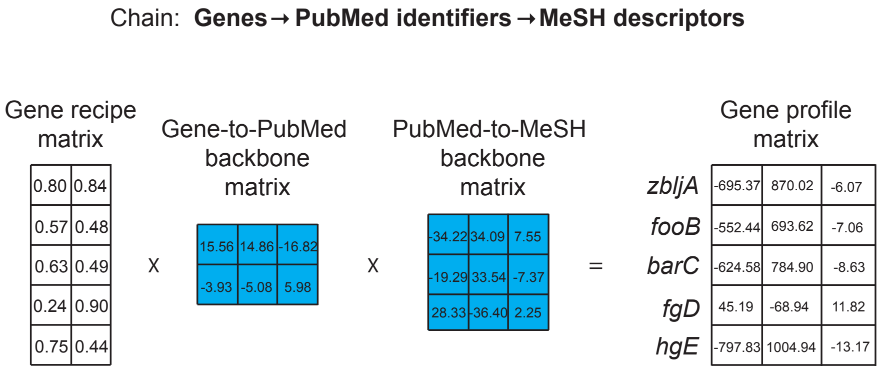

Figure 24 A chain of latent matrices connecting genes and Medical Subject Heading descriptors.

The resulting matrix (Figure 24, right) describes genes in the latent space of MeSH descriptors. Each row in this resulting matrix is a gene profile in the latent MeSH space.

Using chaining we can easily profile any object type in the space of another object type from the data fusion graph. For example, we can profile phenotypes in the context of metabolic pathways, or Gene Ontology terms in the context of RNA-seq experiments. Chaining, as proposed in our work, is a generally applicable procedure that can model relations between any two object types for which there is a path in the data fusion graph.

In order to perform gene prioritization we need seed genes. These are genes known to be relevant to the phenotype of interest. The goal of prioritization is to find other genes with this phenotype, and the approach is to do so based on the similarity of the unknown genes

with the seed genes. Similarity is computed from the gene profiles, which are obtained by Collage from the fusion graph using chains of latent matrices.

To proceed with our example, suppose there is a phenotype that is characteristic of mutant strains in which the mutated genes are *zbljA*, *fooB*, or *barC*. We would like to prioritize other genes from our data sets, that is, genes *fgD* and *hgE*, and estimate which one is the most promising candidate gene that should be the focus of subsequent experimental analysis. Note that in the real world gene prioritization would typically consider thousands of genes instead of just two.

Collage uses chaining of latent matrices (Section 9) to construct gene profiles in the latent space. In our example, the data fusion graph includes seven object types besides "Genes" (Figure 20). For a given target object type we consider all possible chains that start with "Genes" and end at the node of a given target. For example, "Reactome pathways" can be reached from "Genes" via two distinct chains: directly as "Genes" $\rightarrow$ "Reactome pathways" and via an intermediate "Metabolic pathways", "Genes" $\rightarrow$ "Metabolic pathways" $\rightarrow$ "Reactome pathways". We identify a total of 13 chains, that is, 13 paths from the node "Genes" to all other nodes in the graph (Figure 25).

Genes

Genes → Gene Ontology terms
Genes → Reactome pathways → Gene Ontology terms
Genes → Metabolic pathways → Gene Ontology terms
Genes → PubMed identifiers → Gene Ontology terms
Genes → Metabolic pathways → Reactome pathways → Gene Ontology terms

Genes → PubMed identifiers

Genes → PubMed identifiers → MeSH descriptors

Genes → Phenotypes

Genes → Reactome pathways
Genes → Metabolic pathways → Reactome pathways

Genes → RNA-seq experiments

Genes → Metabolic pathways

Figure 25 A list of 13 latent matrix chains that start with "Genes" and end with any other node in the fusion graph.

Chaining thus returns 13 different gene profile matrices. Each row in every profile matrix corresponds to one of five studied genes. Figure 26 provides five of the 13 profile matrices as examples.

Chain: **Genes → Metabolic pathways → Gene Ontology terms**

	Ontology latent component 1	Ontology latent component 2
zbljA	-1.12	1.22
fooB	0.70	-0.25
barC	1.25	-0.63
fgD	-8.03	6.12
hgE	3.12	-1.97

Chain: **Genes → PubMed identifiers**

	PubMed latent component 1	PubMed latent component 2	PubMed latent component 3
zbljA	9.10	7.58	-8.39
fooB	7.06	6.11	-6.81
barC	7.93	6.94	-7.74
fgD	0.27	-0.94	1.28
hgE	9.97	8.94	-10.03

Chain: **Genes → RNA-seq experiments**

	RNA-seq latent component 1	RNA-seq latent component 2
zbljA	2.35	0.07
fooB	1.52	-0.23
barC	1.61	-0.35
fgD	1.63	1.47
hgE	1.73	-0.72

Chain: **Genes → Metabolic pathways → Reactome pathways → Gene Ontology terms**

	Ontology latent component 1	Ontology latent component 2
zbljA	5.70	-2.63
fooB	-5.87	3.77
barC	-9.65	5.96
fgD	52.68	-29.75
hgE	-22.28	13.23

Chain: **Genes → Metabolic pathways → Reactome pathways**

	Reactome latent component 1	Reactome latent component 2
zbljA	1.37	0.32
fooB	-0.90	1.07
barC	-1.60	1.45
fgD	10.07	-4.19
hgE	-3.95	2.63

Figure 26 Examples of derived gene profile matrices.

10 Profile-based similarity scoring

Collage now assesses the similarity between the candidates, *fgD* and *hgE*, and every seed gene by computing the Spearman rank correlation of the corresponding gene profiles (row vectors) from every profile matrix. For our 13 profile matrices and 3 seed genes, this procedure yields a score matrix of size 13×3 of rank correlations for every candidate gene (Figure 27).

Chains	Score matrix for *fgD* — Seed genes: *fooB*	*zbljA*	*barC*	Score matrix for *hgE* — Seed genes: *fooB*	*zbljA*	*barC*
Genes	-1.00	1.00	-1.00	1.00	-1.00	1.00
Genes → Gene Ontology terms	-1.00	-1.00	-1.00	1.00	1.00	1.00
Genes → Reactome pathways → Gene Ontology terms	-1.00	-1.00	-1.00	1.00	1.00	1.00
Genes → Metabolic pathways → Gene Ontology terms	-1.00	1.00	-1.00	1.00	-1.00	1.00
Genes → PubMed identifiers → Gene Ontology terms	-1.00	-1.00	-1.00	1.00	1.00	1.00
Genes → Metabolic pathways → Reactome pathways → Gene Ontology terms	-1.00	1.00	-1.00	1.00	-1.00	1.00
Genes → Phenotypes	-1.00	-1.00	-1.00	1.00	1.00	1.00
Genes → Reactome pathways	-1.00	-1.00	-1.00	1.00	1.00	1.00
Genes → Metabolic pathways → Reactome pathways	-1.00	1.00	-1.00	1.00	-1.00	1.00
Genes → RNA-seq experiments	1.00	1.00	1.00	1.00	1.00	1.00
Genes → Metabolic pathways	-1.00	1.00	-1.00	1.00	-1.00	1.00
Genes → PubMed identifiers	-0.50	-0.50	-0.50	1.00	1.00	1.00
Genes → PubMed identifiers → MeSH descriptors	-1.00	-1.00	-1.00	1.00	1.00	1.00

Figure 27 Similarity scoring between candidate genes, *fgD* and *hgE*, and seed genes, *fooB*, *zbljA* and *barC*.

The highlighted value of -1.00 in the *fgD* score matrix (Figure 27) indicates that the Spearman rank correlation between the profile of the test gene *fgD* and the profile of the seed gene *zbljA* in the profile matrix "Genes" $\rightarrow$ PubMed identifiers $\rightarrow$ MeSH descriptors is -1.00. Intuitively, this means that the membership strengths of *fgD* and *zbljA* to the MeSH descriptors latent components are in a completely reversed order. This suggests that *fgD* and *zbljA* are different with respect to the selected chain. On the other hand, there is a perfect match between the profiles of *hgE* and *barC* in the chain "Genes" $\rightarrow$ "Metabolic pathways". It should be noted that in the real-world, score matrices contain a spectrum of different values as the input data matrices are much larger than in this hypothetical example.

Instead of Spearman rank correlation, we could use other profile-based similarity measures. But it turns out that rank correlation is the most suitable scoring method for our profiling technique. A gene profile measures the strength of the membership of a given gene in the latent components. We are not interested in the absolute value of this strength, but rather in the order of the latent components to which a gene can belong. Hence, rank correlation, where only the order matters, better assesses gene similarity than other correlation-based measures.

11 Overall prioritization

The final step of Collage is to construct a prioritized list of genes based on the score matrices of candidates genes from Section 10. Recall from previous sections that our aim is to assemble a list of genes in which the position of a gene would indicate how "good" this gene would be in further studies of the phenotype of interest. That is, in the prioritized gene list, the most promising candidate genes should be placed near the top and the least promising genes near the bottom of the list.

Collage uses a two-step aggregation scheme of score matrices via medians to estimate gene positions in the list (Figure 28). A two-step aggregation scheme is needed as every candidate gene is associated with a score matrix, *i.e.* a two-dimensional table, as shown in Figure 27. To rank genes, we would like to obtain one score per gene, hence every gene score matrix has to be summarized with a single number. Collage performs aggregation of a score matrix by first computing the median of every column in the matrix (*i.e.* a median candidate gene score across all chains and a selected seed gene) and second, it determines a median of column-wise medians obtained in the first step. We experimented with various L-statistics to summarize similarity score matrices and concluded that order statistics, *e.g.*, the median, produce robust prioritization, are easy to calculate and are often resistant to outliers (data not shown).

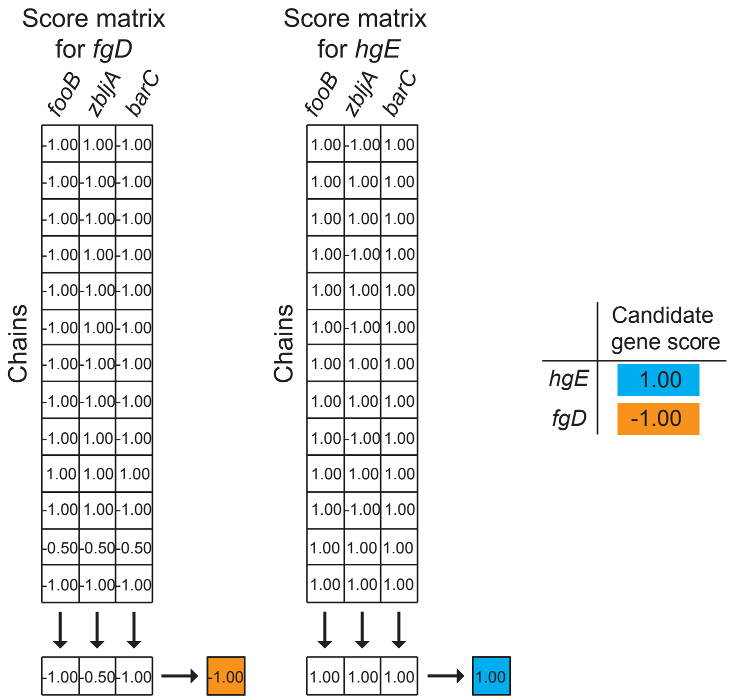

Figure 28 Collage summarizes similarity score matrix of each gene with a single value to estimate the final position of the gene in the prioritized list.

Considering the candidate gene scores, we conclude that *hgE* is a more promising candidate than *fgD* (Figure 28).

Finally, we can obtain nominal P-values by repeatedly randomizing the seed set of genes and re-scoring genes from the pool of candidates. The P-value of a gene position in the list is calculated as a proportion of randomizations with higher aggregated score than the score obtained from the unperturbed seed set.
