# Supplementary figures and images for: Gene Prioritization by Compressive Data Fusion and Chaining
Source: PLoS Comput Biol. 2015 Oct 14;11(10):e1004552. doi: 10.1371/journal.pcbi.1004552 (PMC4605714; doi:10.1371/journal.pcbi.1004552)

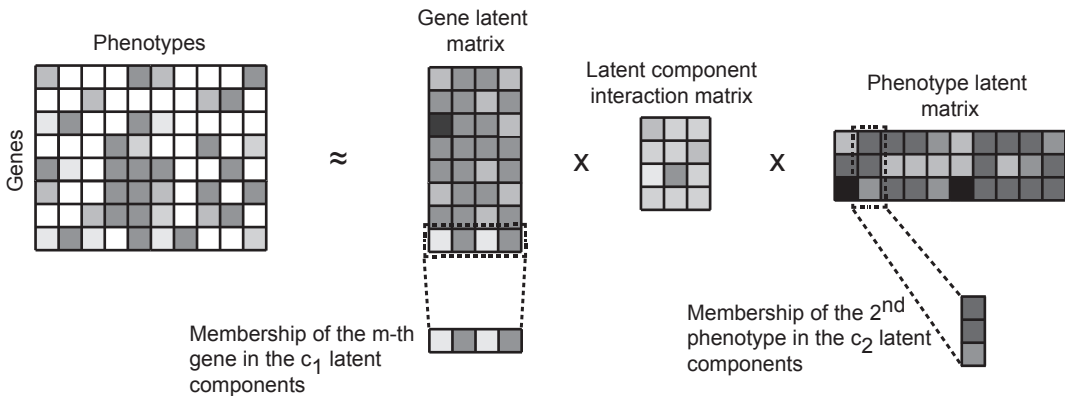
 $R^{m \times n}$ 
 $\approx$ 
 $F^{m \times c_1}$ 
 $\times$ 
 $S^{c_1 \times c_2}$ 
 $\times$ 
 $G^{c_2 \times n}$

Supplement: S1 Fig — The figure illustrates the decomposition of the m × n gene-to-phenotype data matrix R into a product of three low-rank latent matrices, F, S and G. The goal of tri-factorization is to approximate the large-scale gene-to-phenotype matrix with a product of much smaller latent matrices such that the approximation is as good as possible. The original m × n gene-to-phenotype data matrix R is compressed by factorization into a much smaller c 1 × c 2 matrix S of latent (meta) genes in rows and latent (meta) phenotypes in columns. Matrix S is asymmetric and models the interactions between the latent components. To map this compressed representation back to the original domain space, we need two additional matrices, F and G. The m × c 1 nonnegative matrix F maps the space of the meta genes to the space of genes. In each of the m rows, matrix F contains the memberships of a respective gene in each of the c 1 latent components (meta genes). Similarly, each column of the c 2 × n nonnegative matrix G contains the memberships of a respective phenotype in each of the c 2 latent components (meta phenotypes). (PDF) [file pcbi.1004552.s002.pdf]

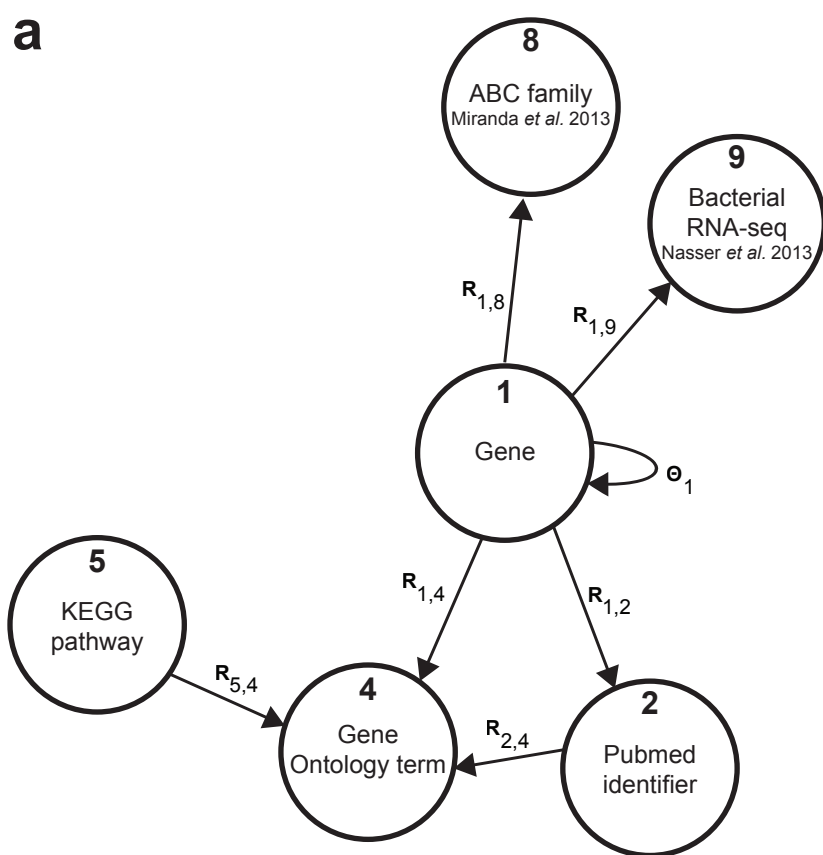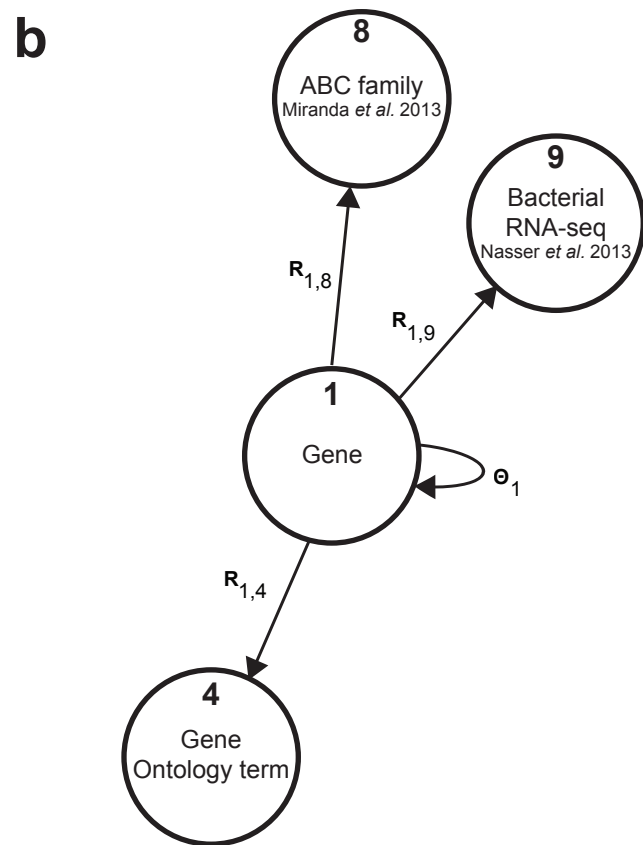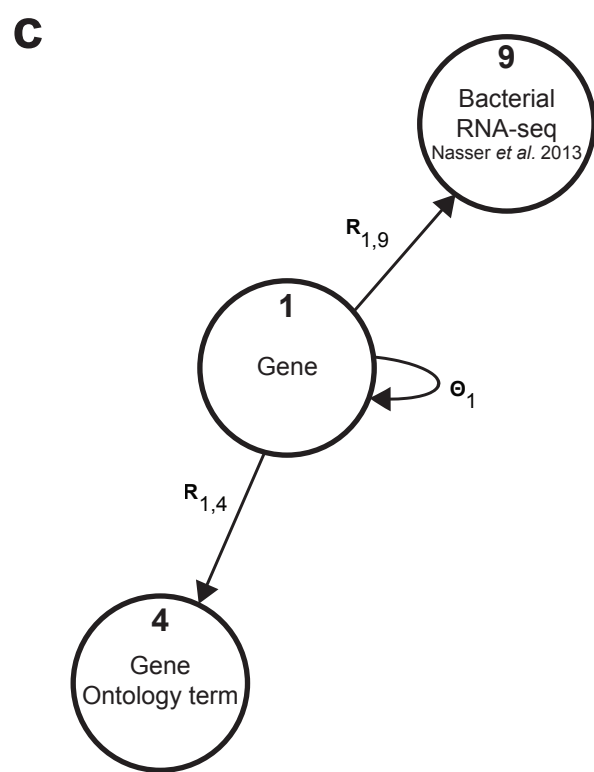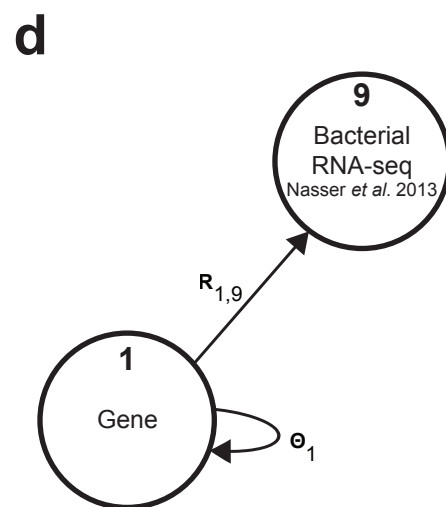

Supplement: S3 Fig — Besides the full collection of data sets (data fusion graph in Fig 2), we have considered data collections with a smaller number of data matrices and studied the impact of this reduction on gene prioritization (S3 Table). We ran gene prioritization analyses by considering subsets of (a) seven, (b) four, (c) three and (d) two data sets that were included in our original study. (PDF) [file pcbi.1004552.s004.pdf]

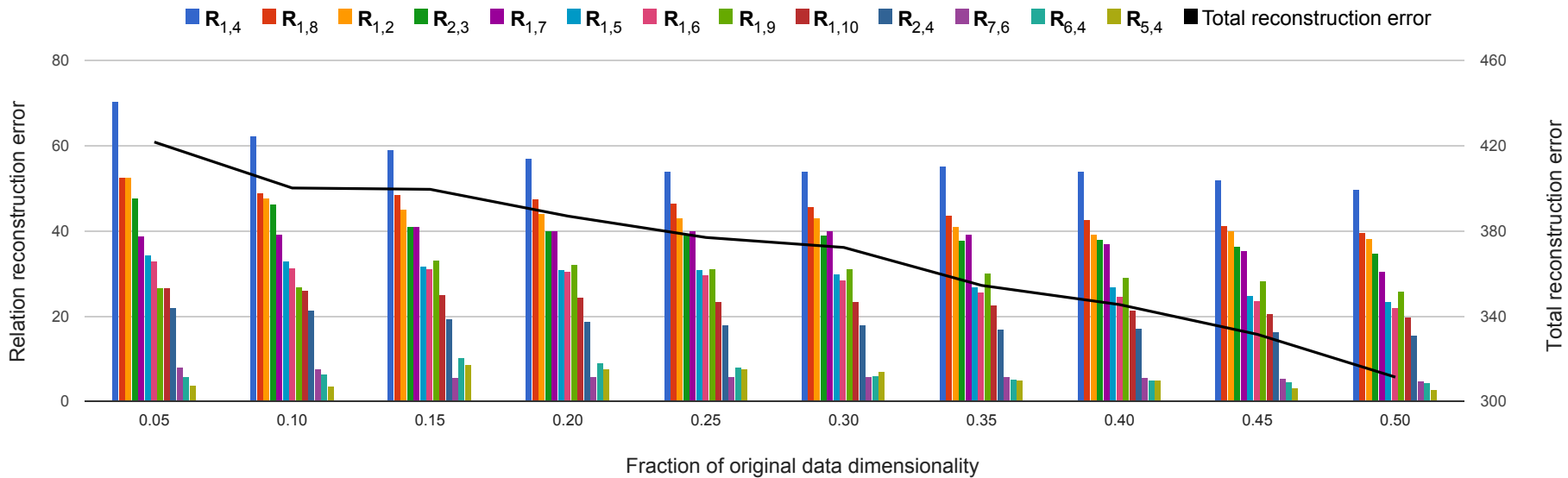

Supplement: S5 Fig — Collective matrix factorization requires specification of latent data dimensionality, that is, a factorization rank for each modeled object type. Factorization rank determines the degree of compression of relation matrices: compression is higher with latent matrices of lower dimensionality. The study of bacterial response gene prioritization in D. discoideum considered data sets describing relationships between objects of 10 different types. Factorization ranks were set through a single parameter k, where the factorization rank was set to kn i for each object type i with n i objects. The value of k was selected by observing the change of the “total reconstruction error” (black line), ∑Ri,j∈𝓡‖Ri,j−R^i,j‖Fro, when varying k between 0.05 and 0.5 (x-axis, “fraction of original data dimensionality”). The reconstruction error was estimated by 50 repetitions of collective matrix factorization, where each repetition was run with a different random initialization of latent matrices. The bars show reconstruction errors of individual data matrices, ‖Ri,j−R^i,j‖ (“relation reconstruction error”). See Fig 2 in the main text for description of the data matrices. We selected k = 0.1 where the maximum kink was attained. This choice resulted in latent data dimensionality (c 1, c 2, c 3, c 4, c 5, c 6, c 7, c 8, c 9, c 10 = (1287, 342, 280, 308, 9, 9, 5, 28, 5, 50) with a limitation on minimum factorization rank set to 5. (PDF) [file pcbi.1004552.s006.pdf]

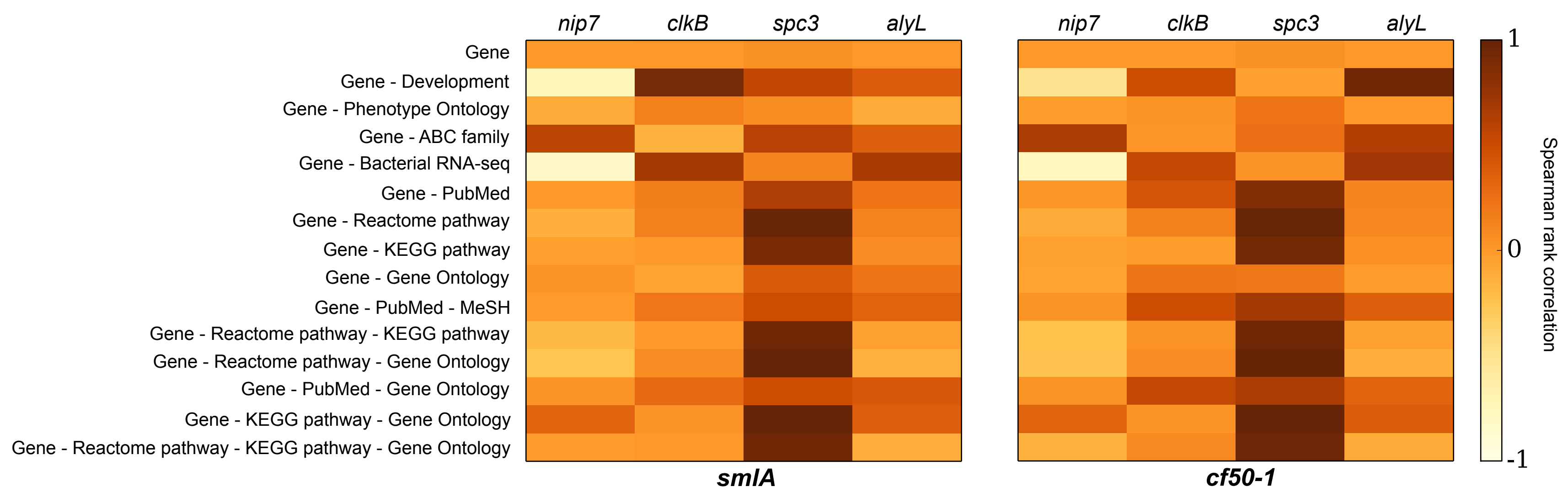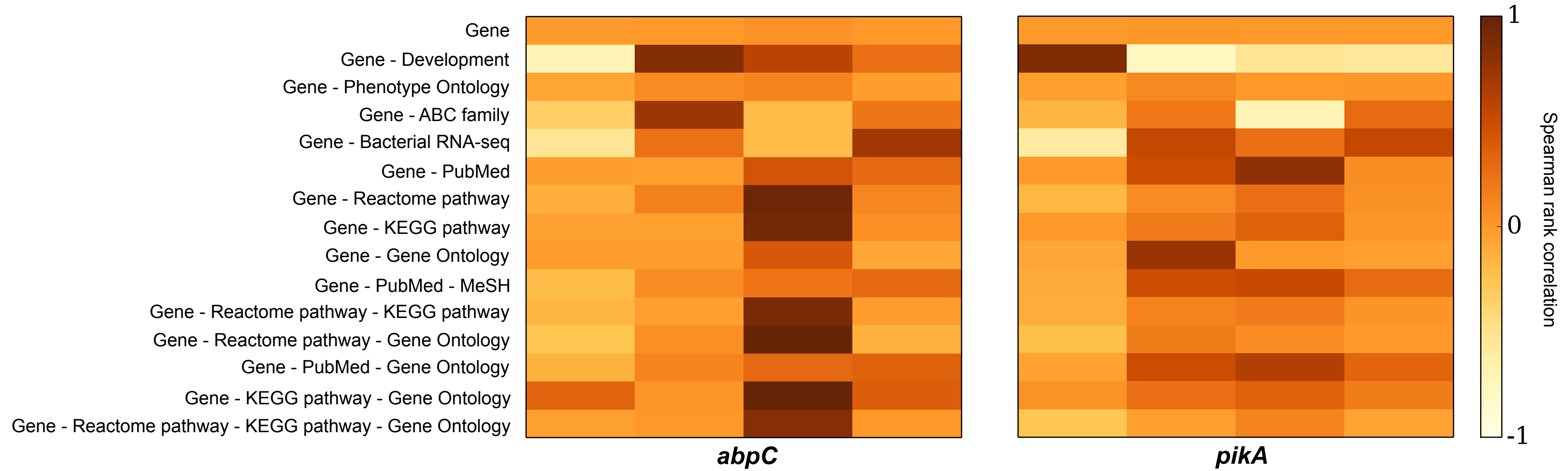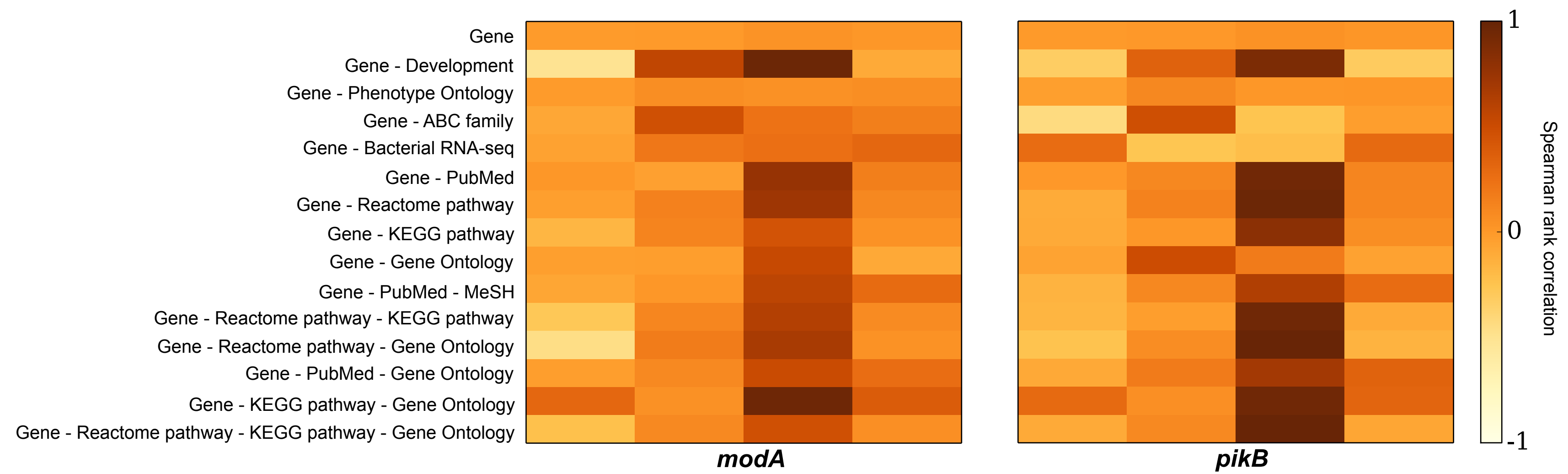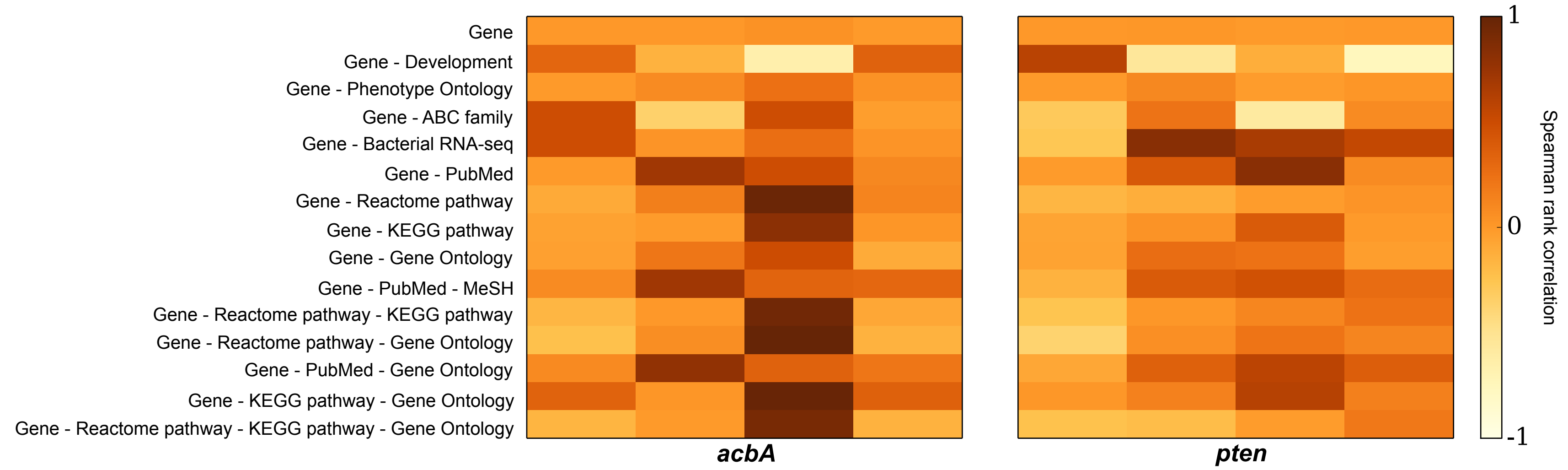

Supplement: S6 Fig — Collage profiles genes through chaining of latent matrices. For a given candidate gene, the profiling procedure yields as many gene profiles (i.e., data vectors corresponding the gene) as there are different chains of latent matrices. Collage then assesses similarity between the candidate gene and a particular seed gene by computing Spearman rank correlation between the respective gene profiles. The figure shows the resulting 15 × 4 (i.e., there were 15 chains and 4 seed genes in our study) similarity score matrix containing rank correlations for each candidate Dictyostelium gene that was validated in the wet laboratory. (PDF) [file pcbi.1004552.s007.pdf]

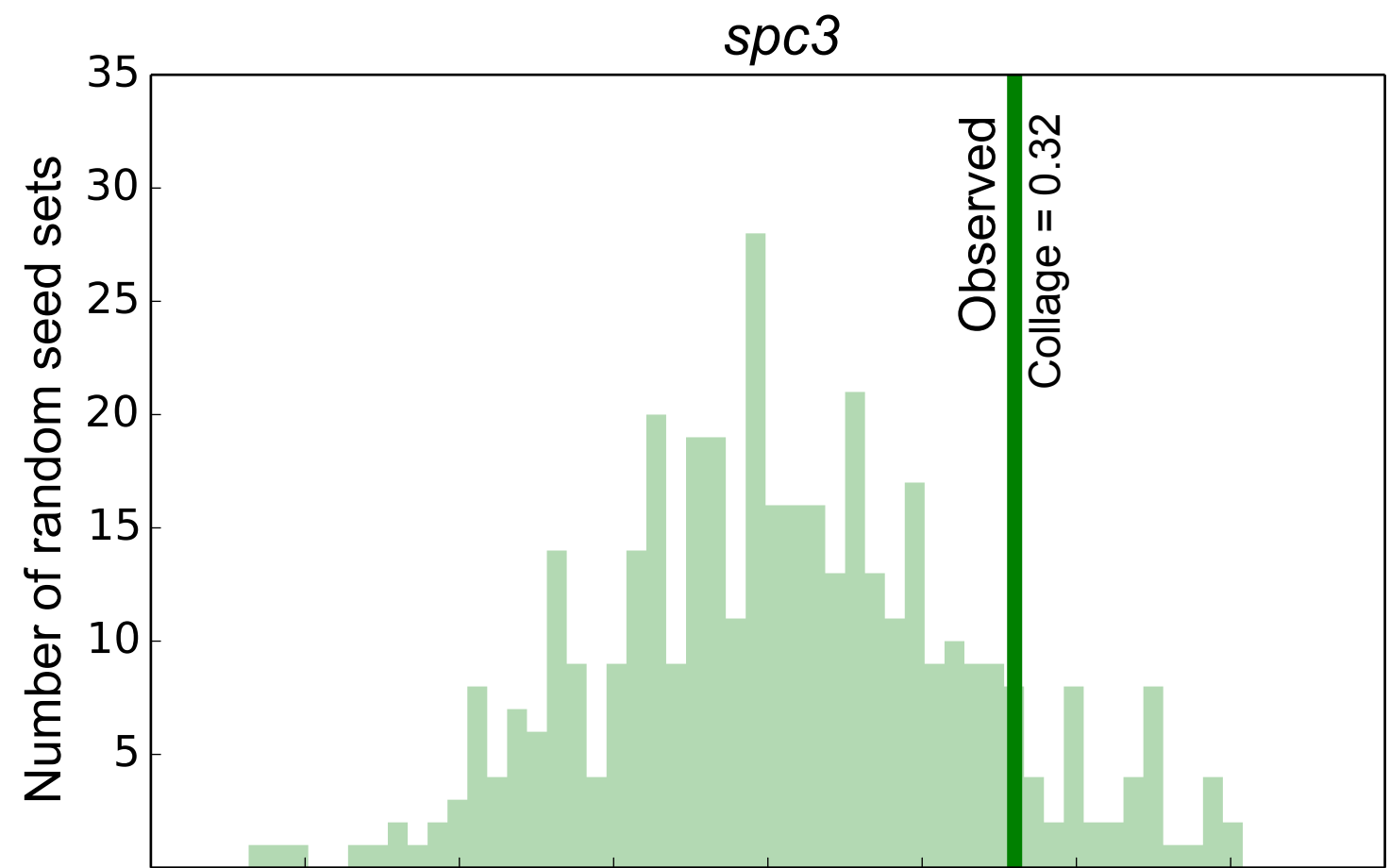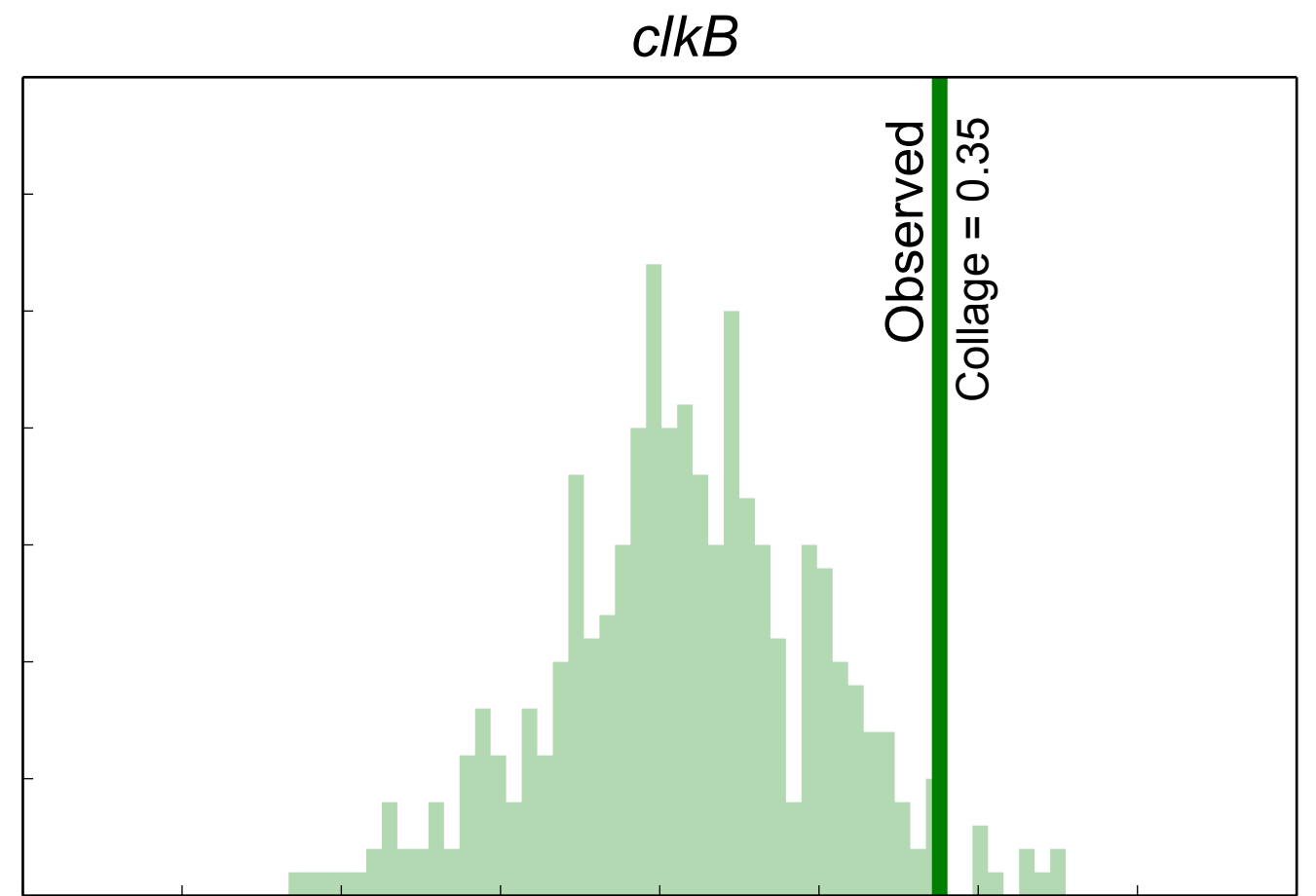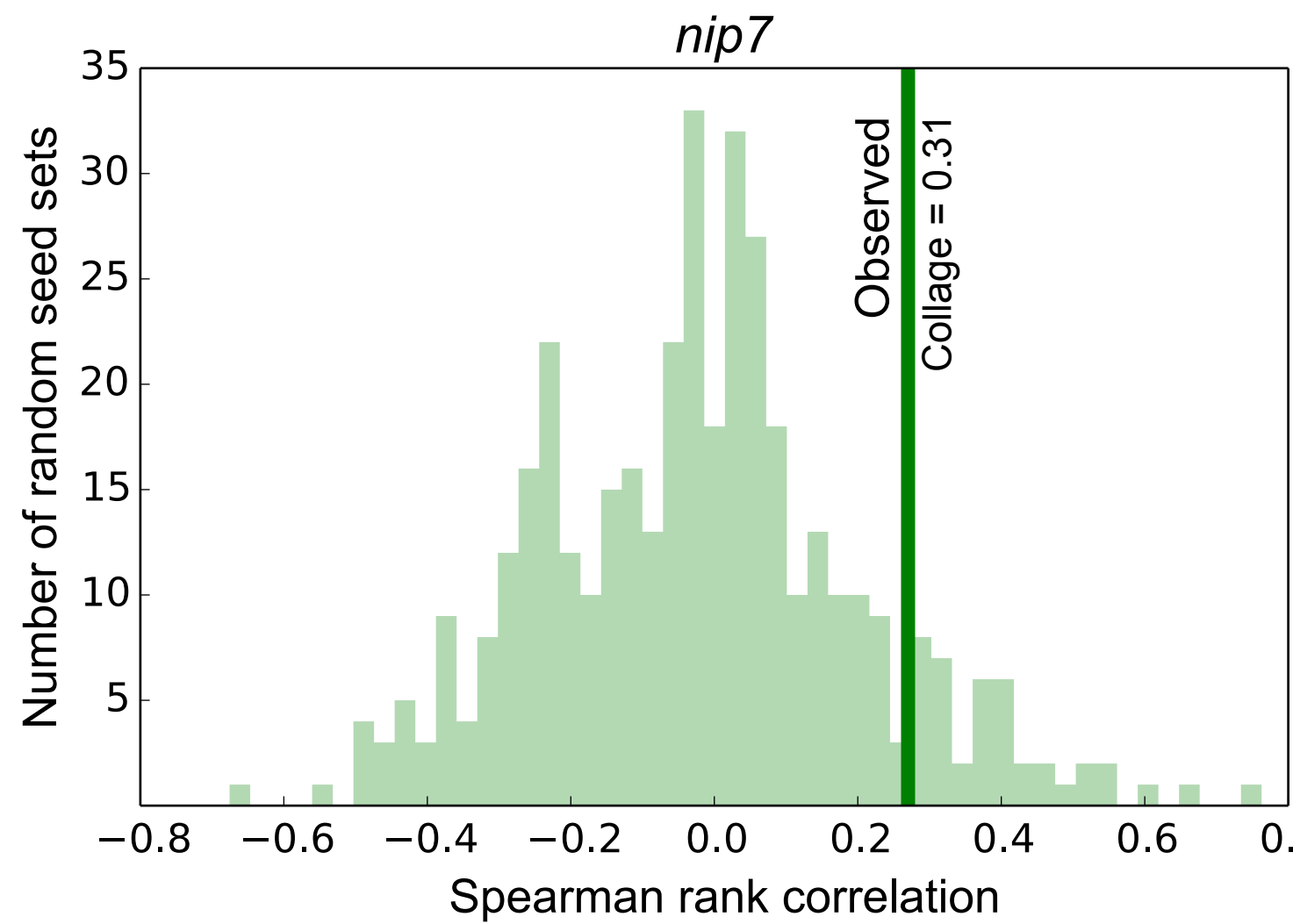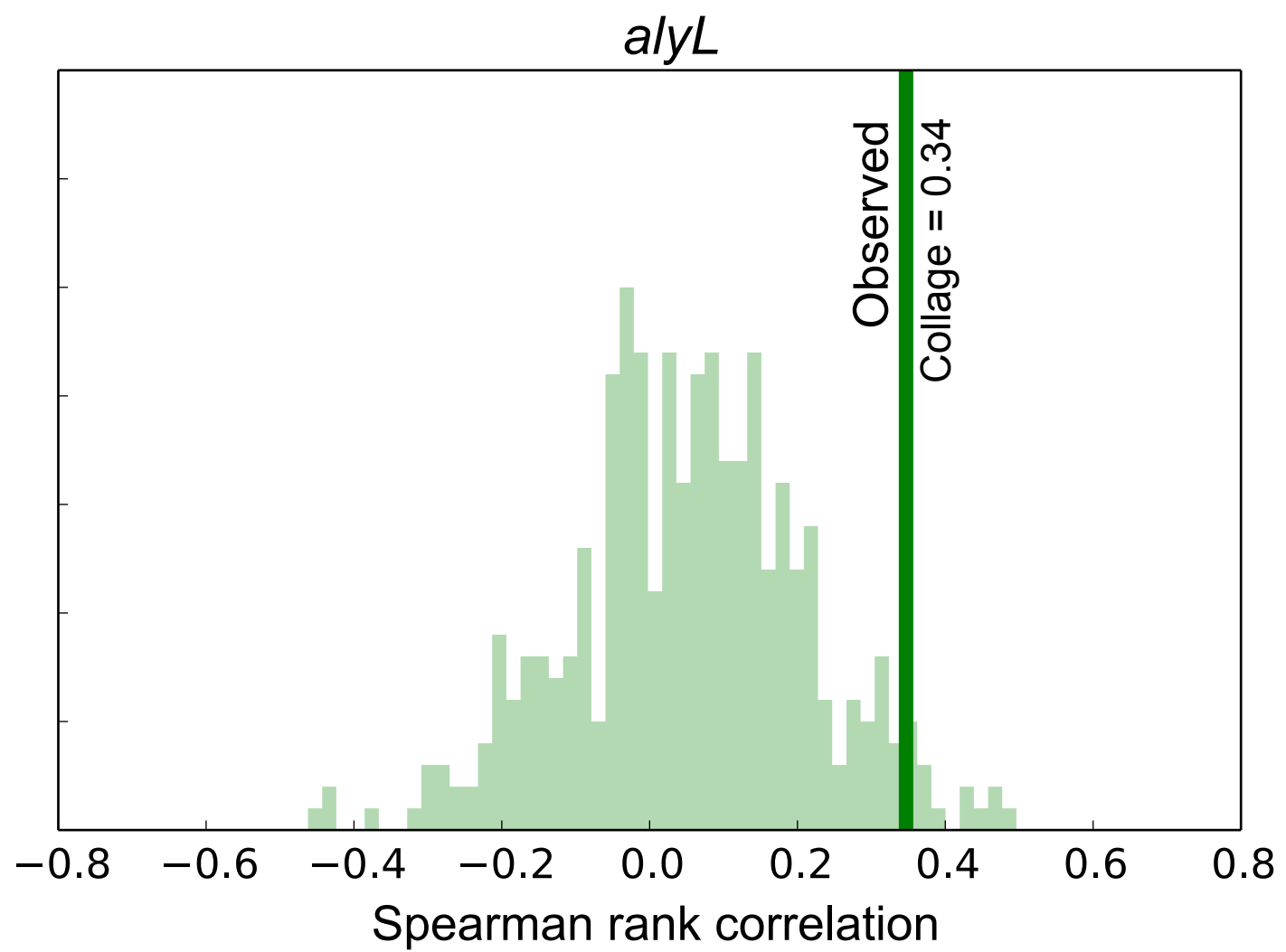

Supplement: S7 Fig — We assessed whether and to what degree the data on seed genes spc3, clkB, nip7 and alyL that were considered for bacterial response prioritization in Dictyostelium vary across individual seed gene. To determine how a given seed gene is different from other seeds, we randomized seed set 400 times and in each randomization used Collage to calculate the similarity score between the given gene and the random set of seed genes (shown in light green). For a given gene we also show its true score as estimated by Collage (e.g., see the vertical line corresponding to the value for spc3 in top left panel) when only the remaining three seed genes (e.g., clkB, nip7 and alyL in top left panel) were considered for scoring. One possible explanation for the substantial amount of variation across seed genes is that these genes were previously identified to be involved in bacterial response pathways using various genetic and genomic methods [22]. They might therefore participate in different aspects of bacterial recognition. Large heterogeneity of seed genes also indicates the difficulty of prioritization task considered here and suggests that consideration of all four seed genes for prioritization is important. (PDF) [file pcbi.1004552.s008.pdf]

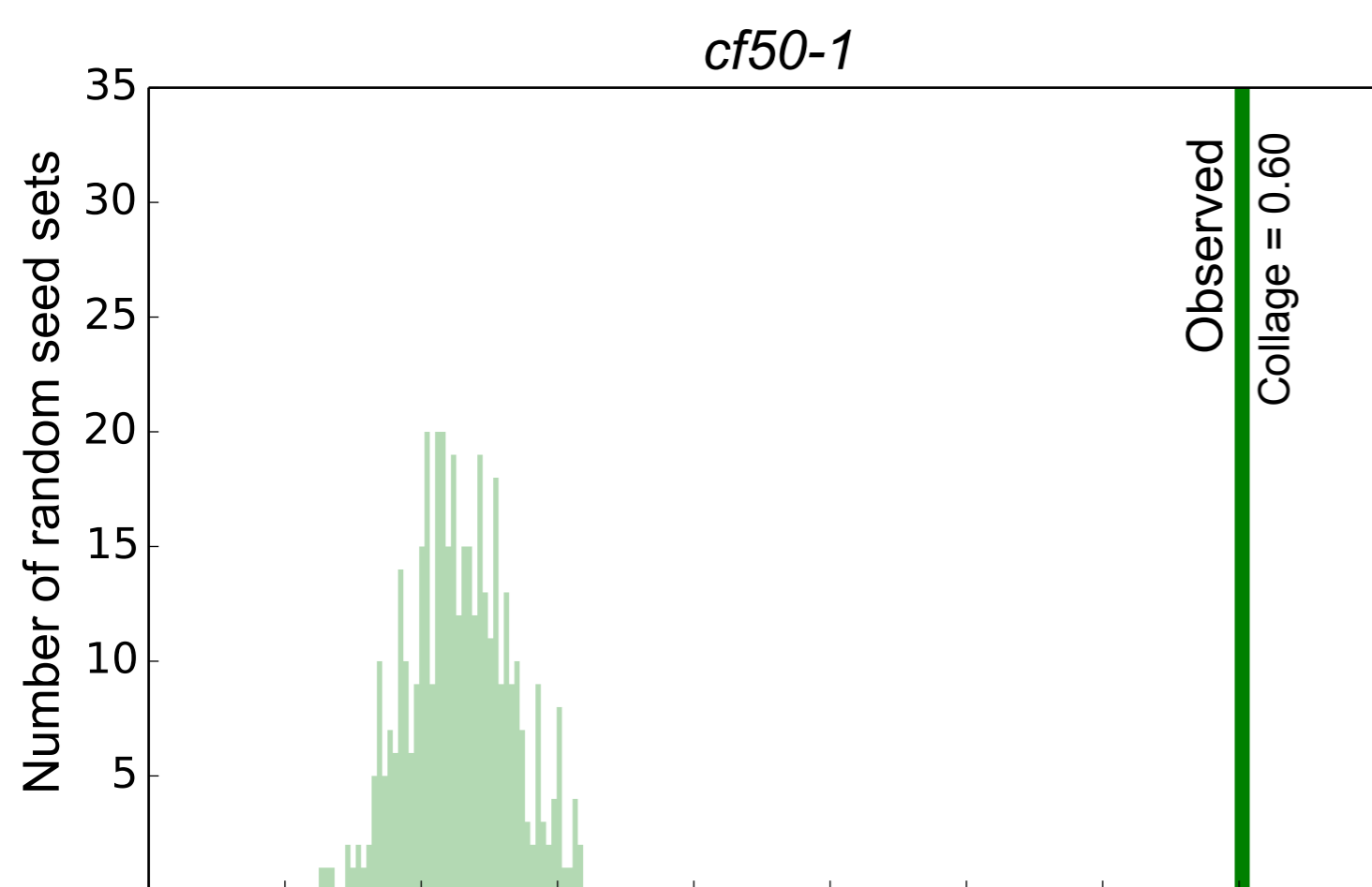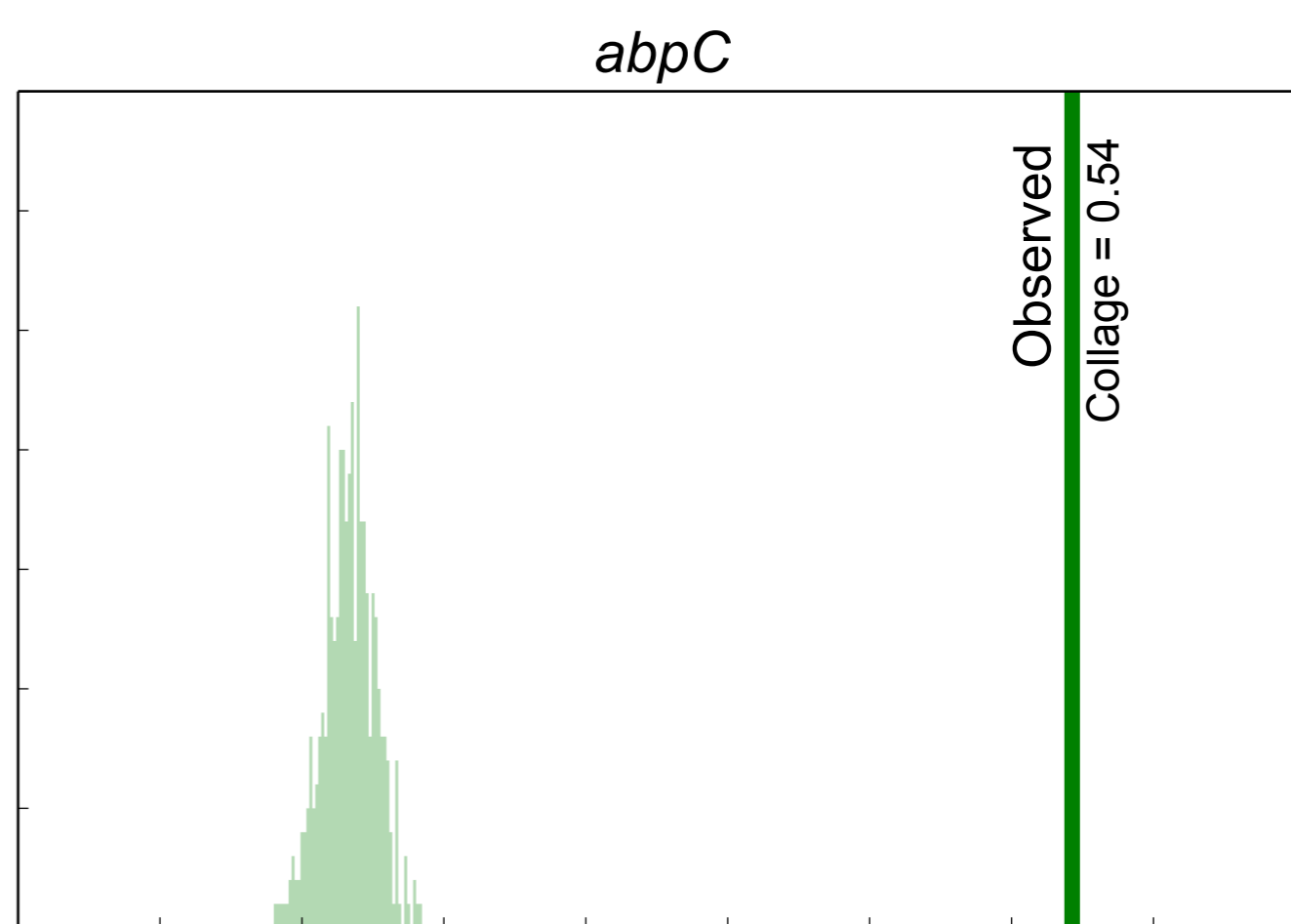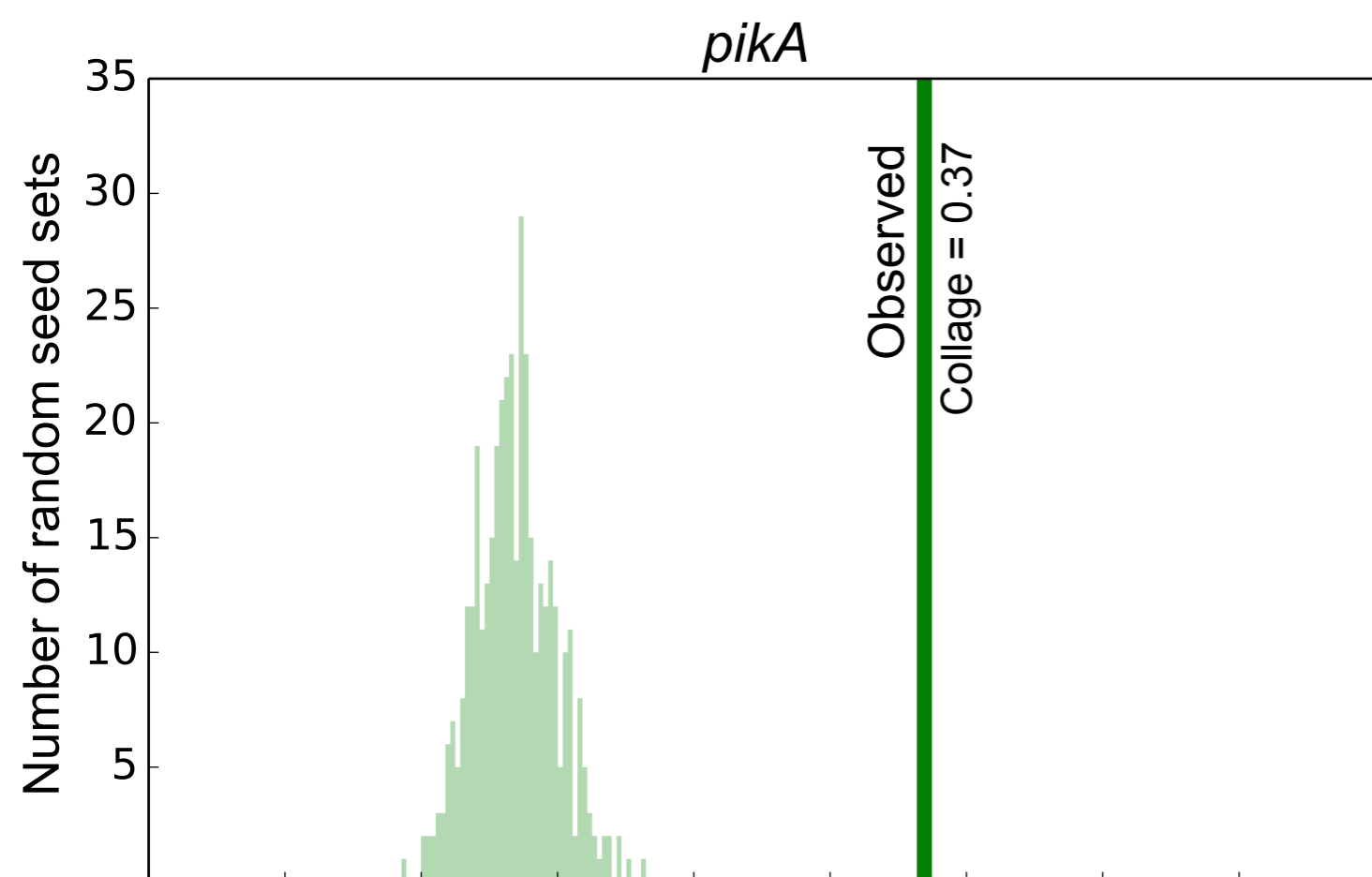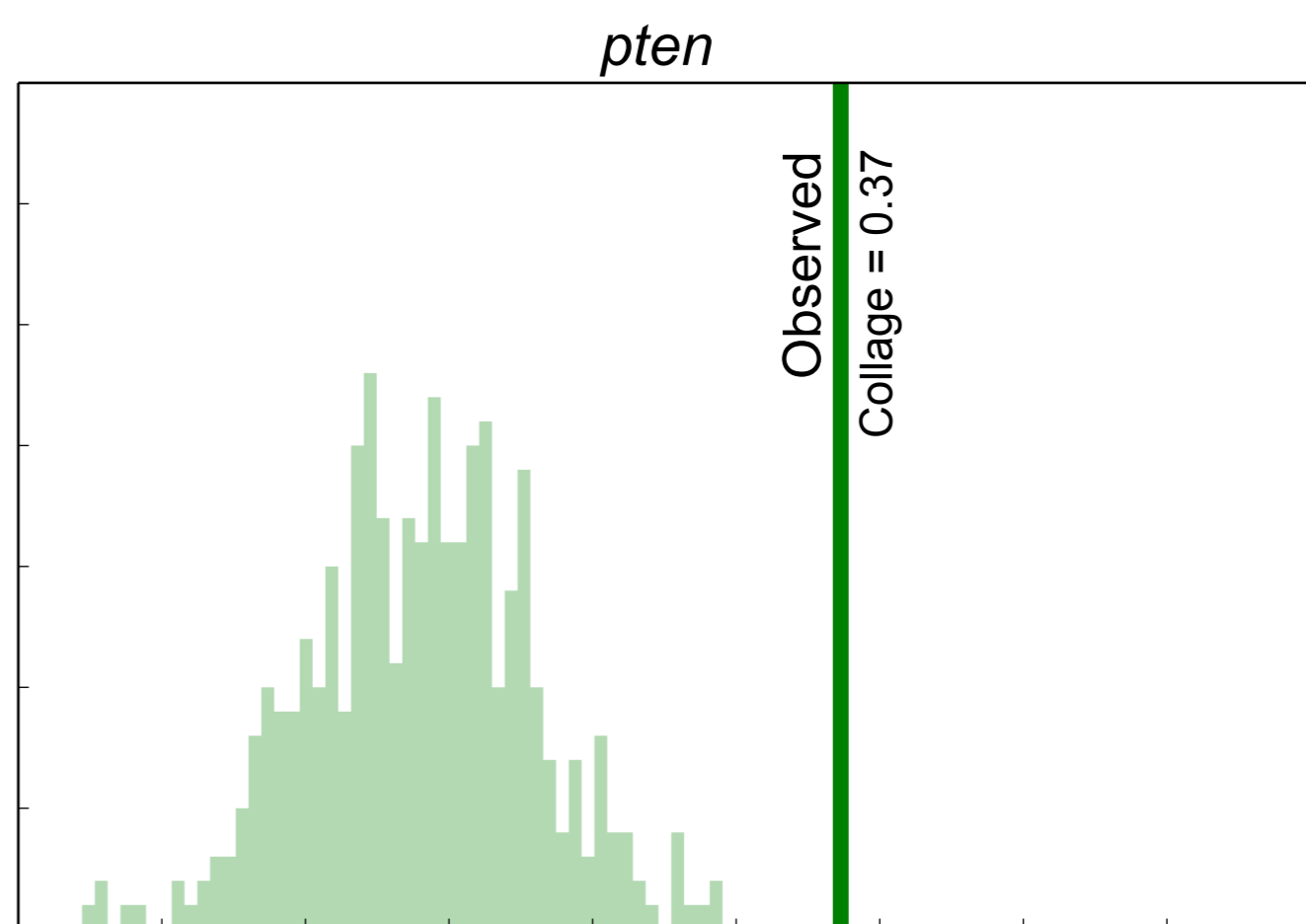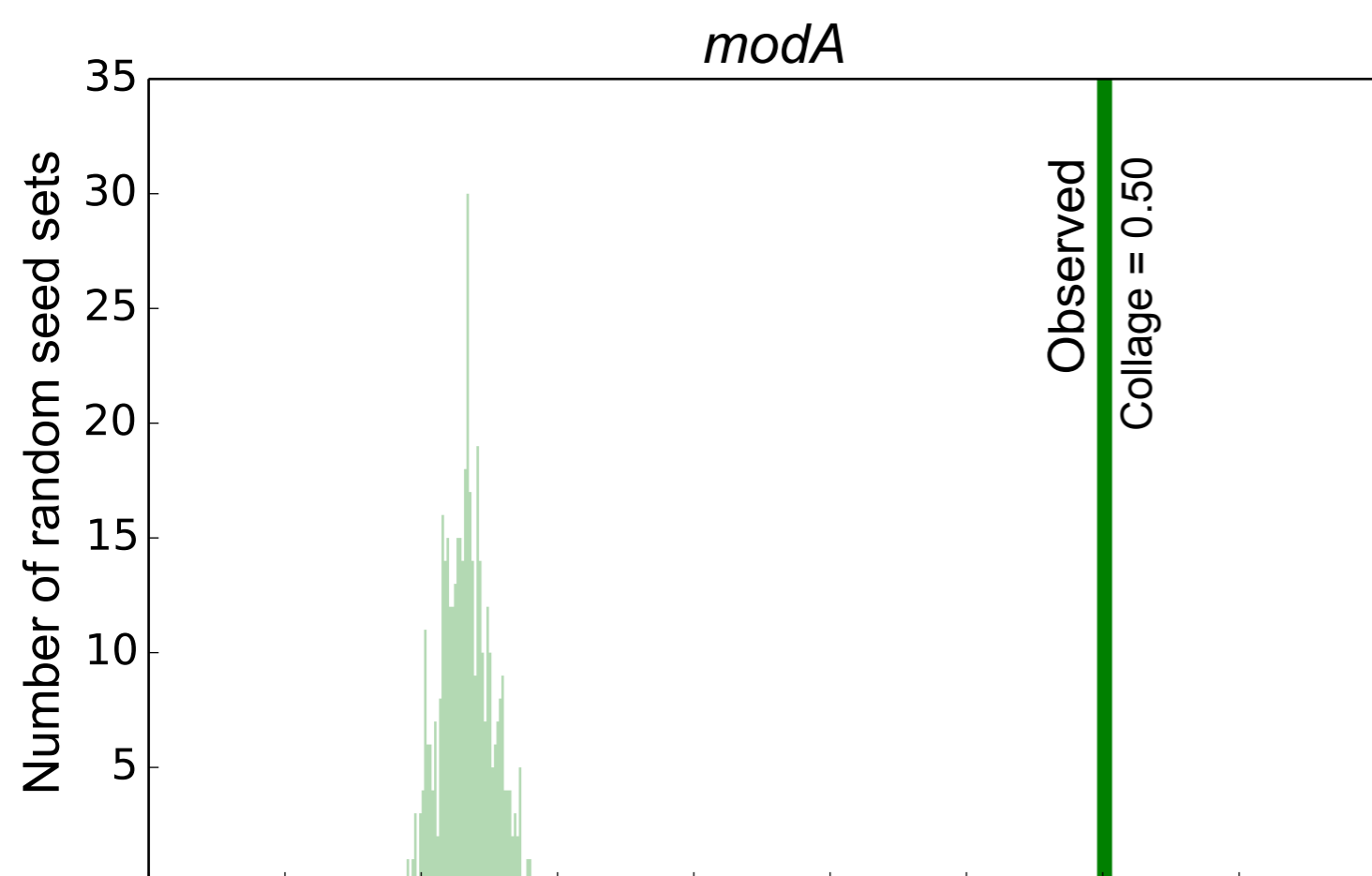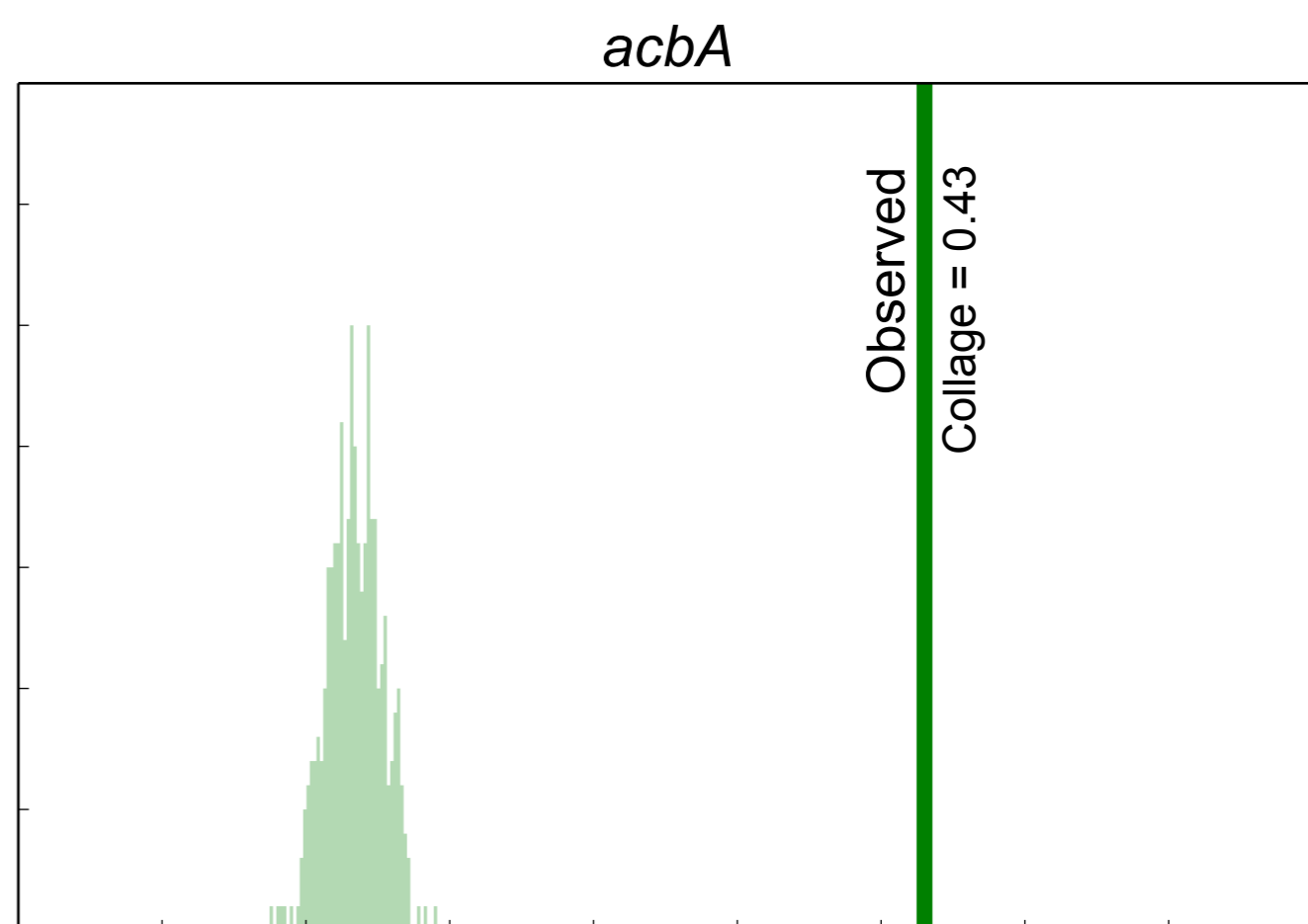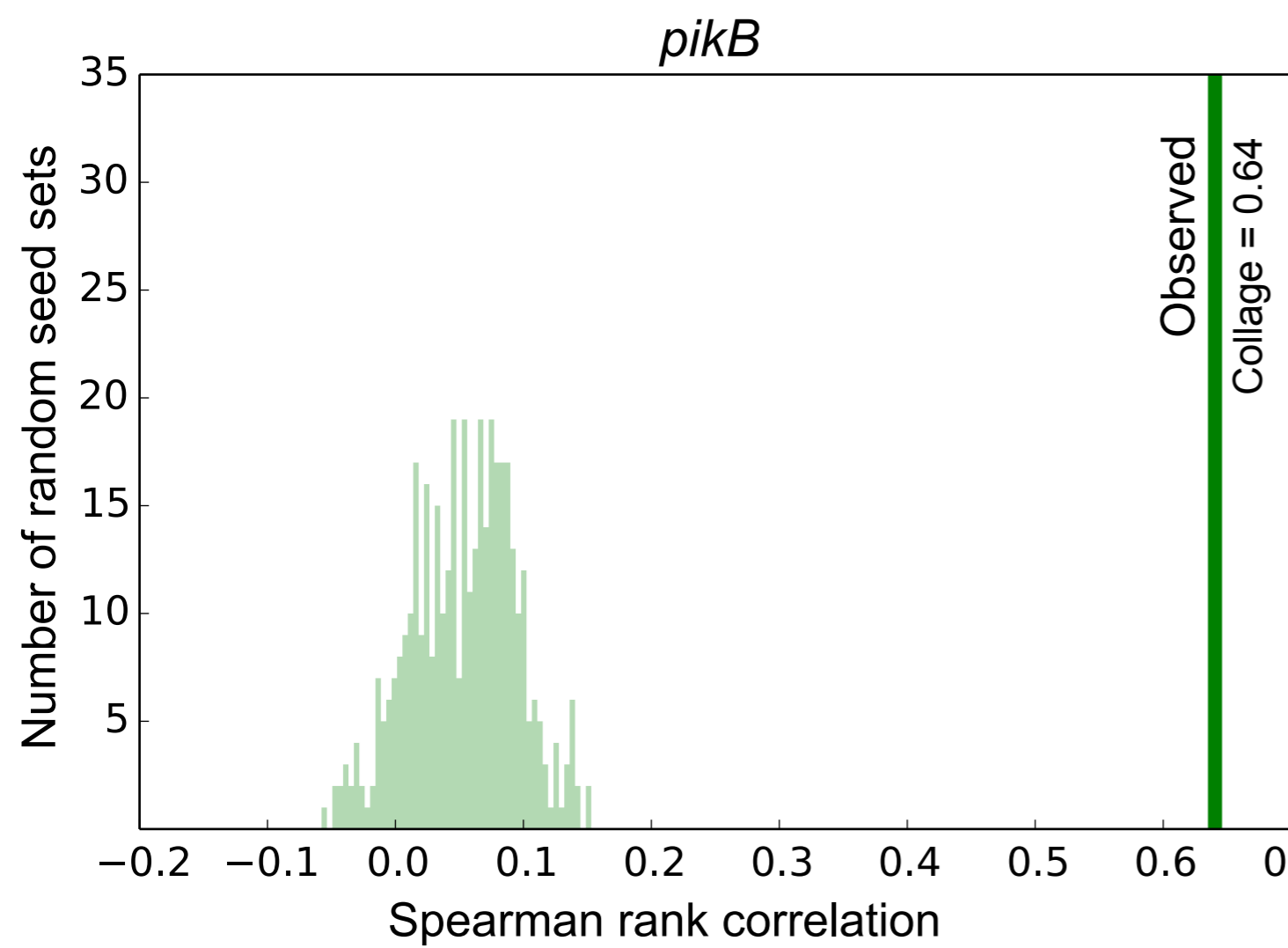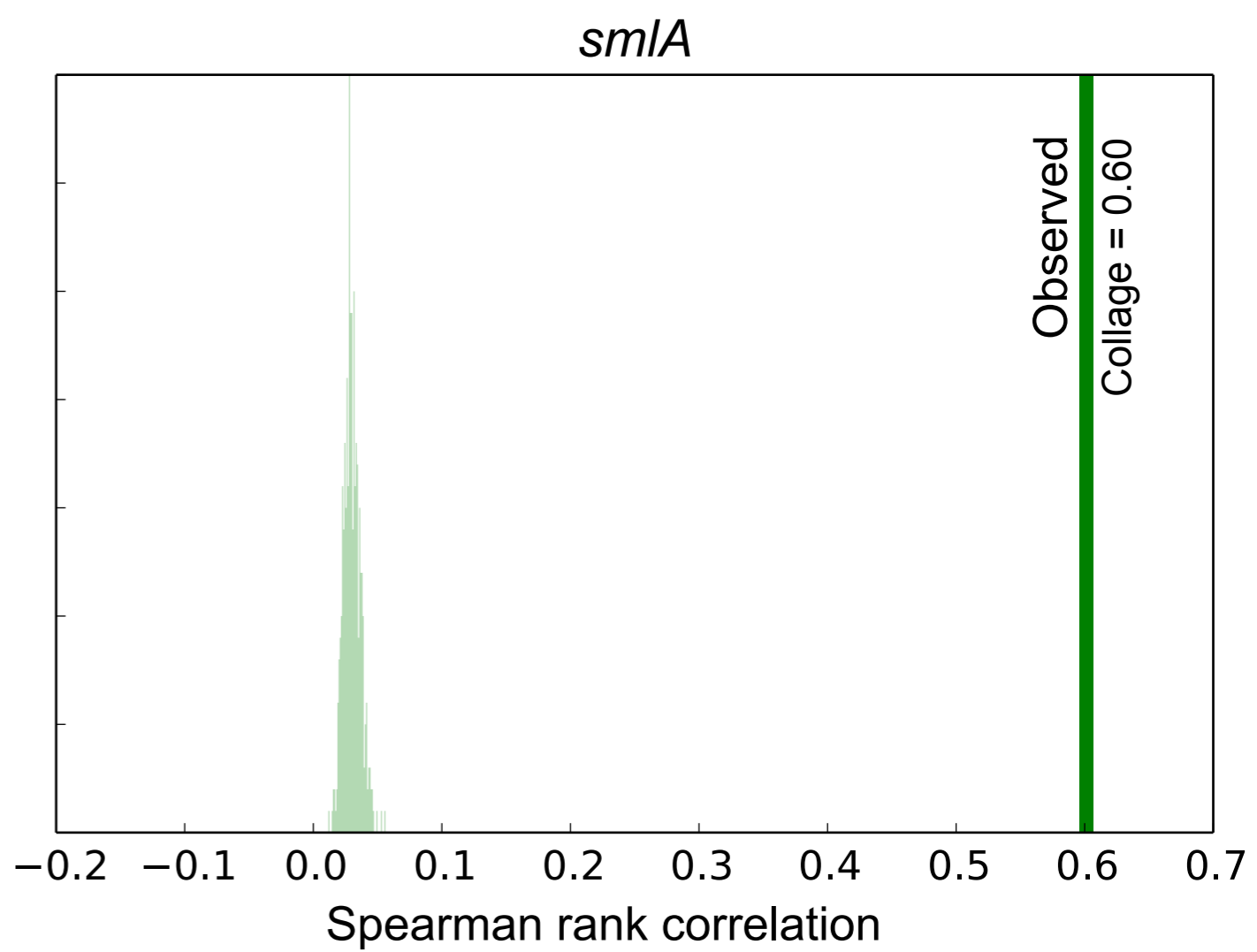

Supplement: S8 Fig — We assessed how alike are candidate genes that were validated in wet laboratory on the basis of their latent data representation estimated by Collage. To determine how a given candidate gene is different from other candidates, we randomized set of candidates considered for experimental validation 400 times and in each randomization used Collage to calculate the similarity score between the given gene and the random set of genes (shown in light green). For a given gene we also show its true score as estimated by Collage (e.g., see the vertical line corresponding to the value for cf50-1 in top left panel) when the remaining seven genes (e.g., abpC, pikA, pten, modA, acbA, pikB and smlaA in top left panel) were considered for scoring. (PDF) [file pcbi.1004552.s009.pdf]

**a**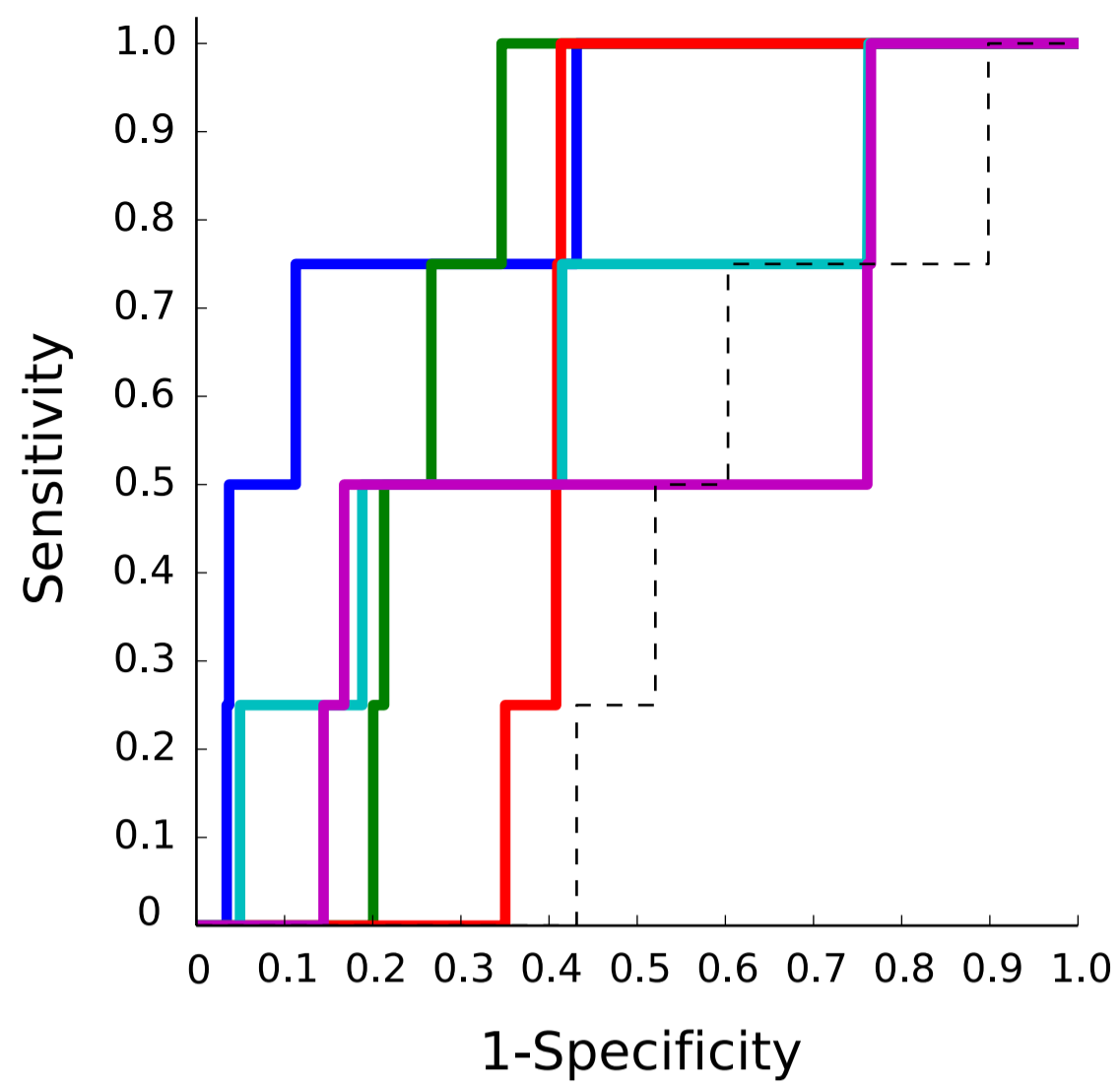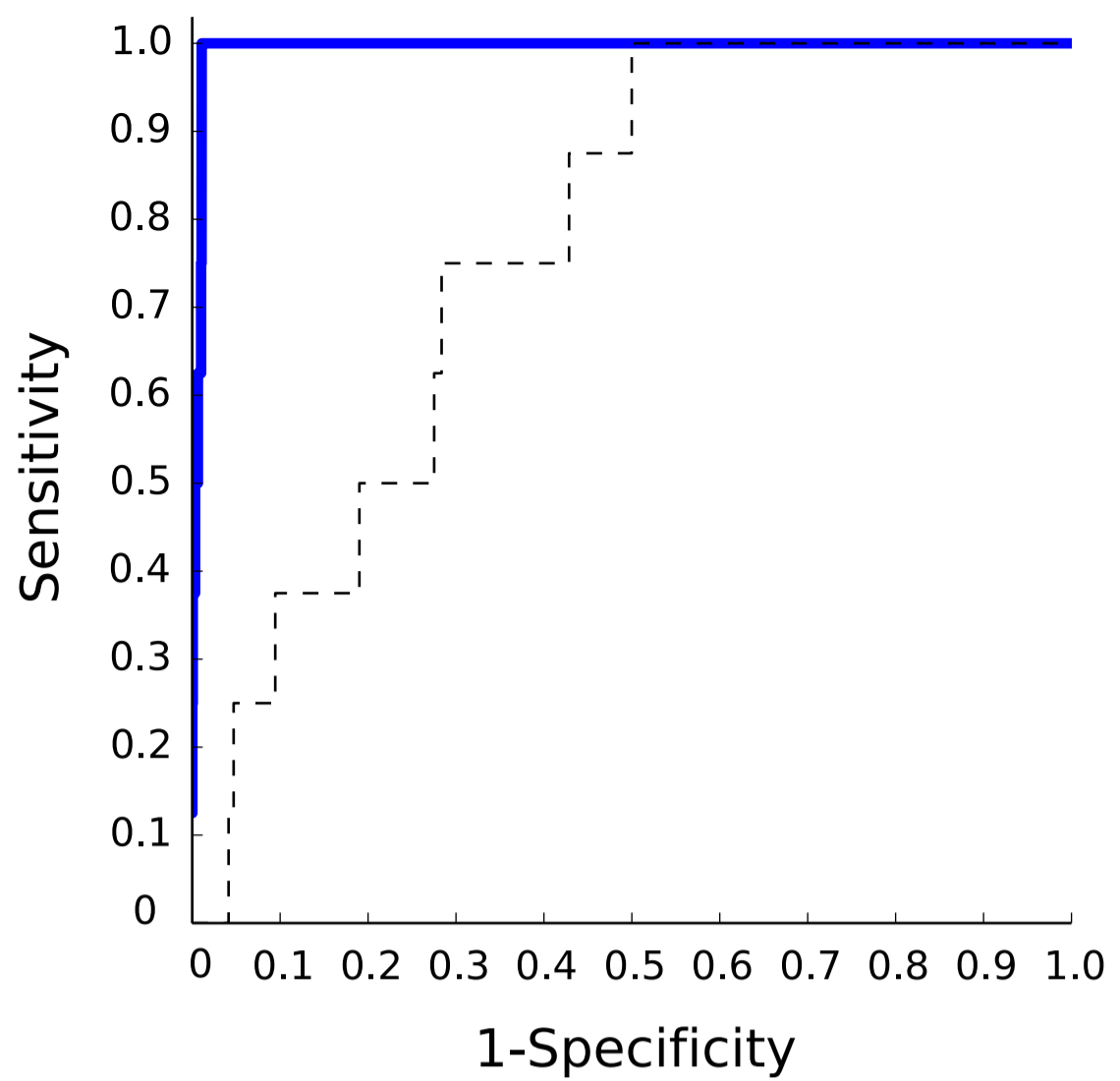**b**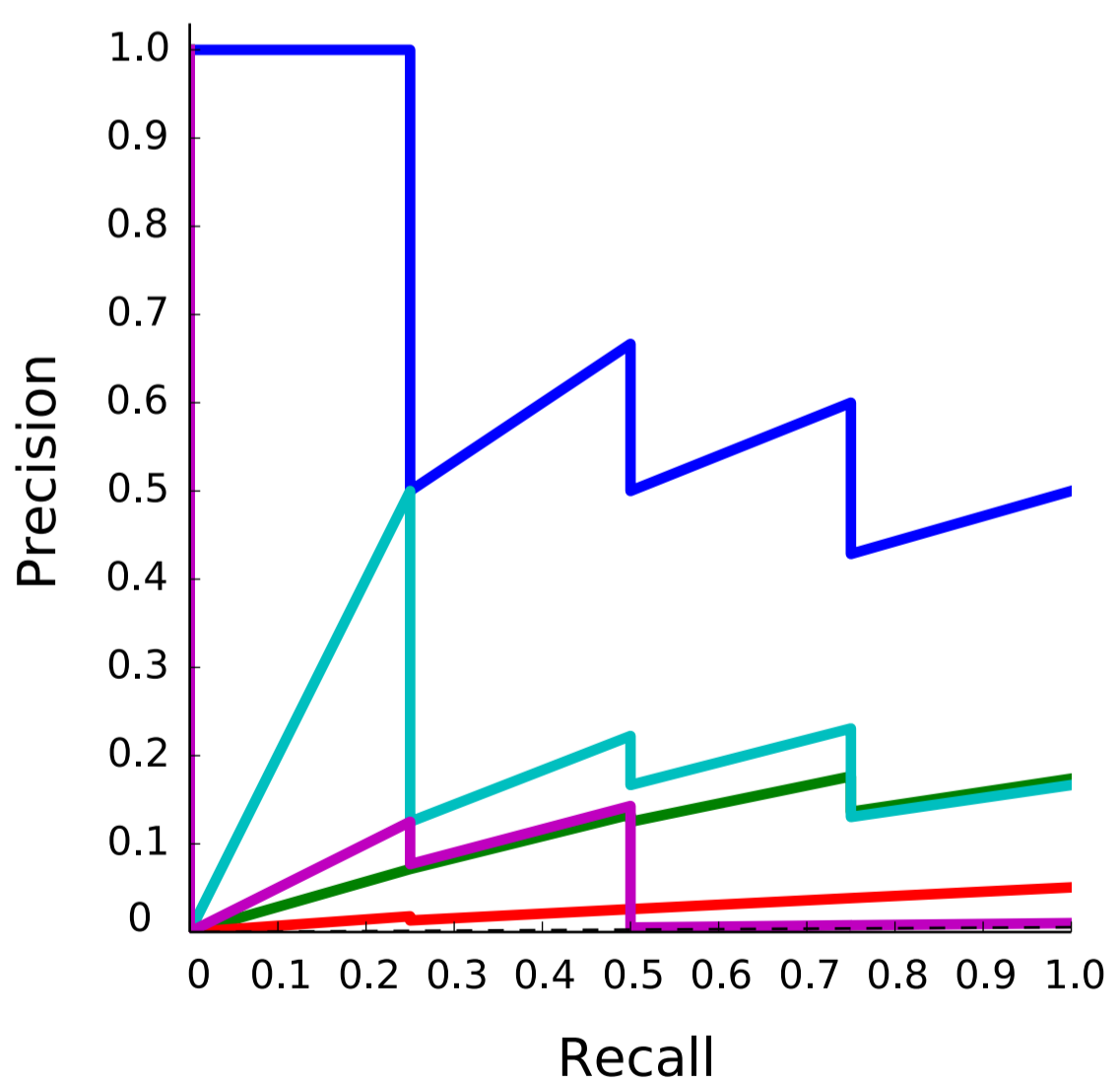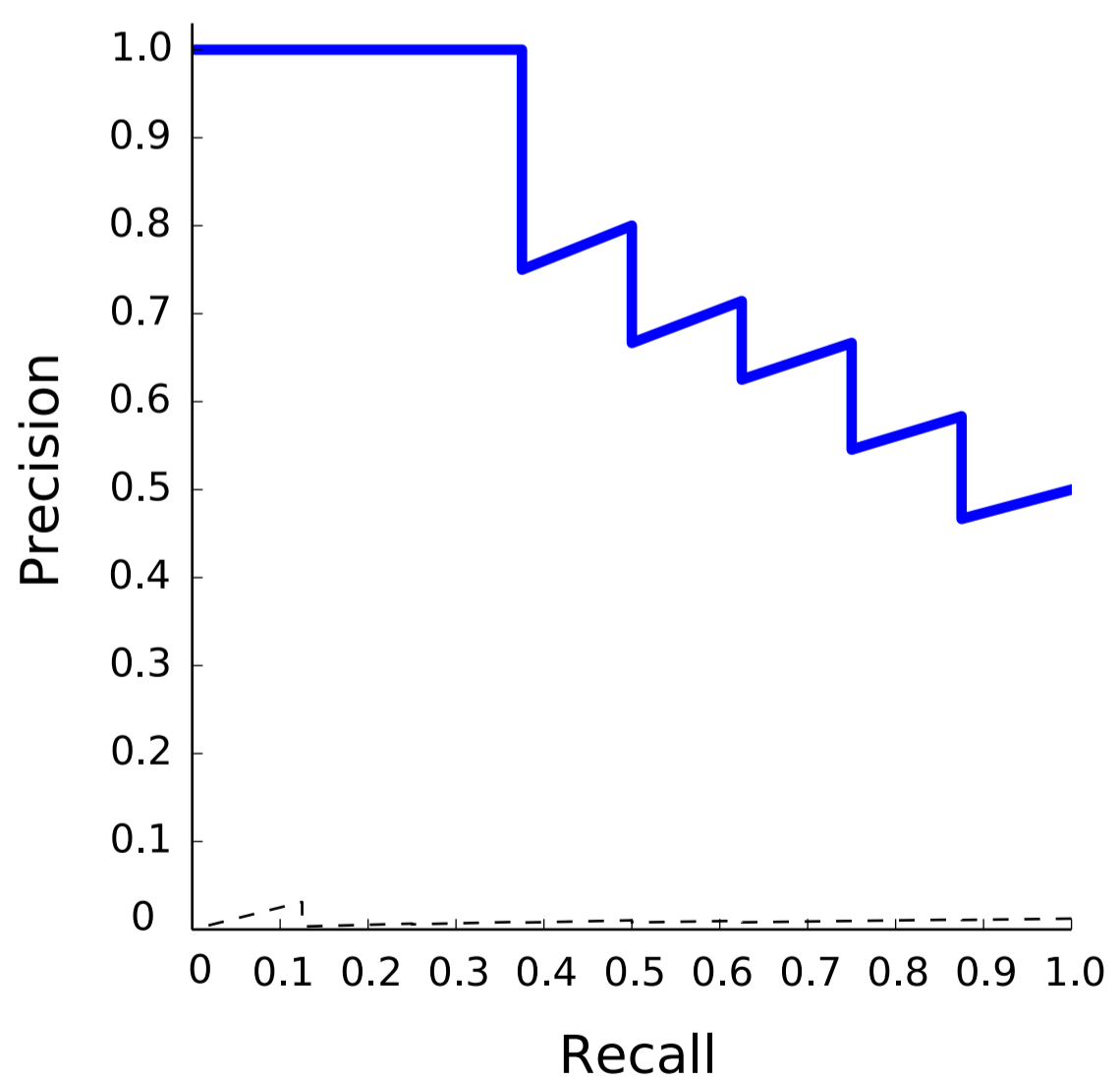

Supplement: S9 Fig — To estimate generalization performance of Collage for bacterial response prioritization in Dictyostelium, we performed cross-validation on seed genes in order to obtain sensitivity and specificity of our model. For this task, the leave-one-out cross-validation fitted well. (Left; a, b) We applied Collage once for each seed gene using all other seed genes as training genes and the left-out gene as a test gene (positive control). For the negative controls, we considered genes, whose mutants are available in the Dicty Stock Center (727 genes from S2 Table). Shown are the (a) ROC curves with the area under the ROC curve statistics, and (b) precision-recall curves with the area under the precision-recall curve statistics based on ranks of left-out genes. The removal of non-gene related data matrices decreased sensitivity and specificity of Collage, suggesting the important ability of Collage to link non-gene related data matrices. The data sources used to construct every performance curve are indicated in S3 Fig. (Right; a, b) (a) Rank ROC curves and (b) precision-recall curves obtained for the leave-one-out cross-validation performed on eight top ranked candidate genes, which were used for testing Collage and proven to be involved in bacterial response pathways. Notice that higher accuracy of results shown in the right panel relative to results in the left panel was expected as the eight top ranked candidate genes have all been predicted using the same seed set (S8 Fig). We would hence like to warn readers about possible confounding effects present in the experiment whose results are shown in the right panel. In both figures, the control ROC curve (black dashed line) was obtained after prioritization with randomly constructed seed sets and by using all data sources. (PDF) [file pcbi.1004552.s010.pdf]
