# Supplementary material for: Gene Prioritization by Compressive Data Fusion and Chaining
Source: PLoS Comput Biol. 2015 Oct 14;11(10):e1004552. doi: 10.1371/journal.pcbi.1004552 (PMC4605714; doi:10.1371/journal.pcbi.1004552)
Supplement: S2 Fig — Matrices in Collage describe relationships between objects of two types. Matrix rows correspond to objects of one type, columns correspond to objects of the other type and matrix elements express the degree of a relationship between the corresponding objects. The figure illustrates matrix representation of six distinct data sets. (a) Degrees of protein-protein interactions from the STRING database are represented in a gene-to-gene matrix. (b) Membership of genes in pathways are represented in binary matrices, one column for each pathway. Binary matrices are also used to associate (c) pathways with gene ontology terms and (d) research articles with Medical Subject Headings. (e) The structure of Gene Ontology can be represented with a real- valued matrix, whose elements report on distance or semantic similarity between the corresponding ontological terms. (f) Levels of gene expression, an experimental data set, are represented by a matrix of stacked gene expression profile vectors. (PDF) [file pcbi.1004552.s003.pdf]

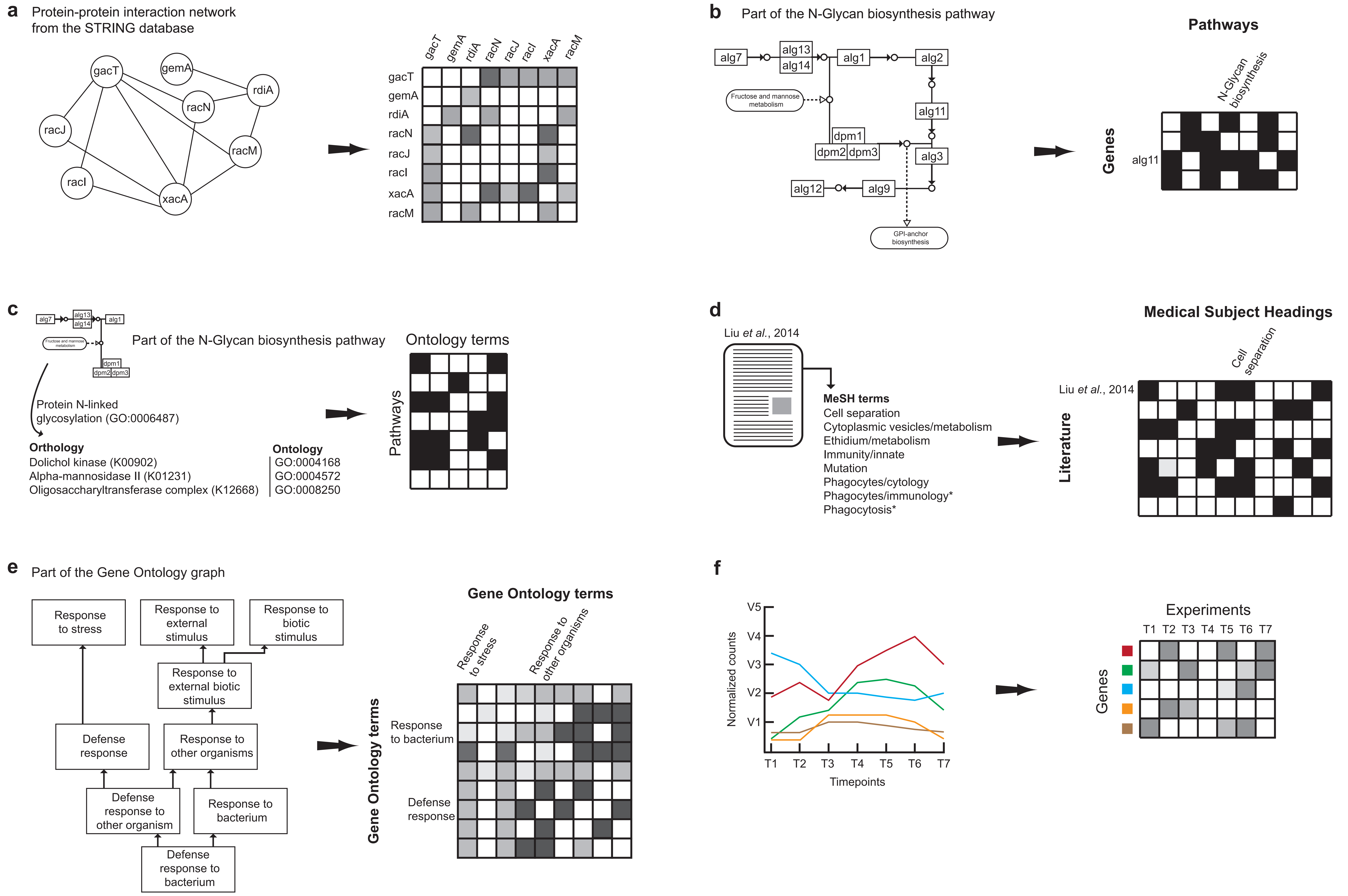
a
Protein-protein interaction network from the STRING database
gacT
gemA
rdiA
racN
racJ
racM
racI
xacA
b
Part of the N-Glycan biosynthesis pathway
alg7
alg13
alg14
alg1
alg2
Fructose and mannose metabolism
alg11
dpm1
dpm2
dpm3
alg3
alg12
alg9
GPI-anchor biosynthesis
Pathways
N-Glycan biosynthesis
Genes
c
Part of the N-Glycan biosynthesis pathway
Protein N-linked glycosylation (GO:0006487)
Orthology
Dolichol kinase (K00902)
Alpha-mannosidase II (K01231)
Oligosaccharyltransferase complex (K12668)
Ontology
GO:0004168
GO:0004572
GO:0008250
Ontology terms
Pathways
d
Liu et al., 2014
MeSH terms
Cell separation
Cytoplasmic vesicles/metabolism
Ethidium/metabolism
Immunity/innate
Mutation
Phagocytes/cytology
Phagocytes/immunology*
Phagocytosis*
Medical Subject Headings
Literature
e
Part of the Gene Ontology graph
Response to stress
Response to external stimulus
Response to biotic stimulus
Response to external biotic stimulus
Defense response
Response to other organisms
Defense response to other organism
Response to bacterium
Defense response to bacterium
Gene Ontology terms
Response to stress
Response to other organisms
Response to bacterium
Defense response
f
Normalized counts
V1
V2
V3
V4
V5
T1
T2
T3
T4
T5
T6
T7
Timepoints
Experiments
Genes
