# Supplementary material for: Gene Prioritization by Compressive Data Fusion and Chaining
Source: PLoS Comput Biol. 2015 Oct 14;11(10):e1004552. doi: 10.1371/journal.pcbi.1004552 (PMC4605714; doi:10.1371/journal.pcbi.1004552)
Supplement: S4 Fig — A prominent approach to approximate a matrix with a system of latent matrices is singular value decomposition (SVD). Factorized models inferred by SVD are prone to overfitting, they cannot guarantee conservation of the desired structural properties of the latent matrices, such as nonnegativity, and they are hard to interpret. These shortcomings of SVD and its variants have spurred the development of regularized learning approaches to matrix factorization. Penalized matrix tri-factorization introduces regularization to tri-factorized latent model. In the figure, the input data matrix is accompanied by two constraint matrices that express degrees of similarity between genes (matrix in yellow and orange) or phenotypes (matrix in blue and green). Constraint matrices guide the inference of latent matrices. In our implementation, elements of constraint matrices that have greater negative values represent must-link constraints, i.e., the corresponding genes (or phenotypes) should have more similar latent profiles. Elements with positive values have the opposite effect—they represent cannot-link constraints by penalizing the latent data model if the corresponding genes (or phenotypes) have similar latent profiles. The matrix factorization algorithm balances between good approximation and adherence to the constraints. (PDF) [file pcbi.1004552.s005.pdf]

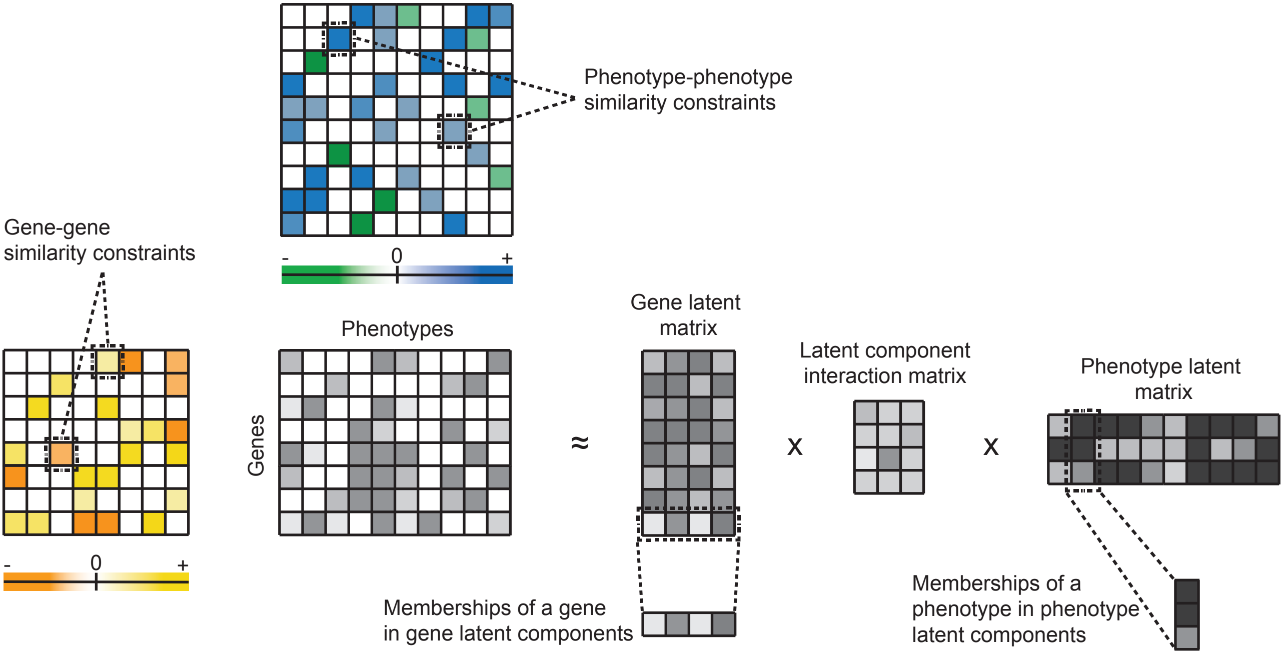
Phenotype-phenotype
similarity constraints
-
0
+
Gene-gene
similarity constraints
-
0
+
Phenotypes
Genes
≈
Gene latent
matrix
x
Latent component
interaction matrix
x
Phenotype latent
matrix
Memberships of a gene
in gene latent components
Memberships of a
phenotype in phenotype
latent components
