# Supplementary material for: Gene Prioritization by Compressive Data Fusion and Chaining
Source: PLoS Comput Biol. 2015 Oct 14;11(10):e1004552. doi: 10.1371/journal.pcbi.1004552 (PMC4605714; doi:10.1371/journal.pcbi.1004552)
Supplement: S3 Table — The table lists the top-30 candidate genes obtained by prioritization by data fusion of 14, 7, 4, 3 and 2 data sets from the data fusion graphs in S3 Fig. Genes in bold are the ones selected for the experimental study. (PDF) [file pcbi.1004552.s013.pdf]

Supplementary Table 3. The impact of modeling circumstantial data on the overall *D. discoideum* bacterial response gene prioritization. The table lists the top-30 candidate genes obtained by prioritization by data fusion of 14, 7, 4, 3 and 2 data sets from the data fusion graphs in **Supplementary Fig. 3**. Genes in bold are the ones selected for the experimental study.

14 data sets	7 data sets	4 data sets	3 data sets	2 data sets
**cf50-1**	shkA	rbsk	DDB_G0271348	arpE
**smlA**	DDB_G0288519	DDB_G0272614	DDB_G0268872	DDB_G0278663
**acbA**	**pten**	DDB_G0278163	DDB_G0287153	DDB_G0281091
pirA	**cf50-1**	qtrt1	yelA	DDB_G0267742
rps10	**acbA**	DDB_G0279263	sibD	**pten**
**abpC**	**smlA**	DDB_G0286079	DDB_G0272380	DDB_G0277937
tirA	DDB_G0288947	adprh	DDB_G0288519	DDB_G0271120
DDB_G0272184	DDB_G0275057	DDB_G0279939	dnaja1	yipf1
**pikB**	tra2	DDB_G0272382	rabT2	DDB_G0267494
vps46	sibC	gdt6	DDB_G0292920	DDB_G0272016
**pikA**	rbsk	ku80	sibB	eif2b1
swp1	DDB_G0281967	arpF	DDB_G0278163	empB
ggtA	**pikA**	cofD-1	adprh	DDB_G0291926
DDB_G0288519	DDB_G0272614	DDB_G0288551	lvsG	vps13l
**pten**	DG1112	empB	DDB_G0285403	cenB
DDB_G0288551	adprh	gacV	tpsB	ku80
tra2	DDB_G0288551	DDB_G0294629	ndm	DDB_G0288161
DDB_G0286429	DD_G0283989	swp1	DDB_G0281559	DDB_G0268232
dscA-1	dscA-1	gbqA	DDB_G0275671	rbsk
cinC	gdt6	DDB_G0291926	DDB_G0288963	atg12
udpB	piaA	DDB_G0273031	gbqA	vps46
sfbA	DDB_G0279145	DDB_G0287643	uduA1	DDB_G0290575
**modA**	DDB_G0290575	DDB_G0268876	acrA	DDB_G0267958
DDB_G0287399	abcA1	abkD	arpE	DDB_G0287153
prmt5	DDB_G0272380	DDB_G0268206	uduC	gacV
dpoA	DDB_G0272801	DDB_G0279145	DG1098	DDB_G0276509
DDB_G0278663	lipA	DDB_G0272380	DDB_G0273451	DDB_G0279971
psiP	cepG	plbG	adprt3	usp39
sibC	lvsG	cct3	DDB_G0288031	DDB_G0280477
DDB_G0291926	uduA1	psiP	yipf1	DDB_G0292098
