# Supplementary material for: Gene Prioritization by Compressive Data Fusion and Chaining
Source: PLoS Comput Biol. 2015 Oct 14;11(10):e1004552. doi: 10.1371/journal.pcbi.1004552 (PMC4605714; doi:10.1371/journal.pcbi.1004552)
Supplement: S4 Table — The notation of the data sets (“Data matrix” column) is the same as in the data fusion graph (Fig 2). All relation data matrices were normalized before data analysis such that the Frobenius norm of every row profile was equal to 1. This type of data normalization was also considered in our previous studies with collective matrix factorization. Preprocessed data sets are provided with the project related code and are available from GitHub repository (http://github.com/marinkaz/collage). (PDF) [file pcbi.1004552.s014.pdf]

Supplementary Table 4. Summary of data sets considered for bacterial response gene prioritization in *D. discoideum*. The notation of the data sets ("Data matrix" column) is the same as in the data fusion graph (**Fig. 2**). All relation data matrices were normalized before data analysis such that the Frobenius norm of every row profile was equal to 1. This type of data normalization was also considered in our previous studies with collective matrix factorization. Preprocessed data sets are provided with the project related code and are available from GitHub repository (http://github.com/marinkaz/collage).

Data matrix	Matrix size	Description
$\boldsymbol{R}_{1,4}$	12,873 x 3,083	The version of gene annotations from the Gene Ontology (Ashburner *et al.* 2000) maintained within the Orange system was obtained through Orange Bioinformatics add-on (http://github.com/biolab/orange-bio) in April 2014.
$\boldsymbol{R}_{1,8}$	12,873 x 282	RPKM-normalized RNA-seq transcriptional profiles of 35 *abc*-transporter mutant strains and wild-type AX4 strain in two replicates and at four different time points during development (Miranda *et al.* 2013).
$\boldsymbol{R}_{1,2}$	12,873 x 3,424	Associations of *D. discoideum* genes to research articles from PubMed accessed in August 2013 through the DictyBase website (http://dictybase.org/Downloads; "List of PubMed IDs and associated genes"). The list of relevant articles from PubMed and associated genes is updated monthly by the DictyBase.
$\boldsymbol{R}_{1,5}$	12,873 x 99	Memberships of *D. discoideum* genes in the Kyoto Encyclopedia of genes and genomes (KEGG) pathways (Kanehisa *et al.* 2014) accessed through Orange Bioinformatics add-on (http://github.com/biolab/orange-bio). We used metabolic pathway information that was

		maintained within the Orange system in April 2014.
$\boldsymbol{R}_{1,6}$	12,873 x 92	We downloaded *D. discoideum* pathways from the Reactome database (Croft *et al.* 2014). We considered all information from the Reactome database up to August 2013. For every pathway we obtained a list of associated proteins ("Molecules" view in the Reactome pathway browser) and then mapped the protein names to the *D. discoideum* gene identifiers (DDBGs) using mapping from the DictyBase website (http://dictybase.org/Downloads; "DDB-DDB_G-UniProt mapping").
$\boldsymbol{R}_{1,7}$	12,873 x 14	Normalized gene expression profiles analyzed with RNA-seq and measured at 4-hour intervals during 24-hour *D. discoideum* development in two replicates (Parikh *et al.* 2010).
$\boldsymbol{R}_{1,9}$	12,873 x 8	Normalized abundances of gene transcripts in two replicates and four different bacterial growth conditions analyzed with RNA-seq (Nasser *et al.* 2013).
$\boldsymbol{R}_{1,10}$	12,873 x 503	The version of gene annotations from the Phenotype Ontology was downloaded from the DictyBase website (http://dictybase.org/Downloads; "All curated mutants with phenotypes") in March 2014. The information about mutant phenotypes is updated monthly by the DictyBase.
$\boldsymbol{R}_{2,4}$	3,424 x 3,083	Cross-references of research articles from the PubMed and Gene Ontology terms. We counted the words from the Gene Ontology term names that occurred in the abstracts of articles from PubMed database. The set of considered PubMed articles was determined by the available list on the DictyBase website (see descriptions

		for $\boldsymbol{R}_{1,4}$ and $\boldsymbol{R}_{1,2}$).
$\boldsymbol{R}_{2,3}$	3,424 x 2,804	Assignments of Medical Subject Headings (MeSH) to research articles as maintained by the PubMed database. We considered research articles that were considered relevant according to the DictyBase (see description for $\boldsymbol{R}_{1,2}$). We queried the PubMed to obtain up-to-date MeSH annotations using the Biopython interface (http://biopython.org) in March 2013.
$\boldsymbol{R}_{5,4}$	99 x 3,083	Cross-references of the KEGG orthology groups and Gene Ontology terms. We mapped KEGG pathways (see description for $\boldsymbol{R}_{1,5}$) to KEGG orthology groups (fetched from the KEGG website, http://www.genome.jp/kegg/ko.html) in July 2013 and used the mapping between ortholog groups and Gene Ontology terms as specified by the KEGG pathway browser.
$\boldsymbol{R}_{6,4}$	92 x 3,083	Cross-references of the Reactome pathways and Gene Ontology terms as defined by the "Generic Gene Ontology Slim Subset" (http://www.geneontology.org/page/download-ontology; "goslim_generic.obo"). Generic GO slim subset was downloaded from Gene Ontology website in July 2013.
$\boldsymbol{R}_{6,5}$	92 x 99	Cross-references of the Reactome and KEGG pathways by counting common words in KEGG pathway names and Reactome pathways display names. See descriptions for $\boldsymbol{R}_{1,5}$ and $\boldsymbol{R}_{1,6}$ for information on which KEGG and Reactome pathways were considered.

Θ_1	12,873 x 12,873	Protein-protein interaction data from the STRING v.9 database (Franceschini *et al.* 2013) accessed through Orange Bioinformatics add-on (http://github.com/biolab/orange-bio). We used interaction data as was maintained within the Orange system in April 2014. Ortholog mapping of *Dictyostelium* genes onto interactions from other organisms is performed within STRING. STRING relies on manually curated orthology database, the Clusters of Orthologous Group (COGs), and an automatic method resembling the COG procedure to assign functional associations between genes across different genomes. For a detailed description about construction of the similarity matrix we refer readers to Zitnik & Zupan, 2015.
